# Acute and early-onset cardiotoxicity in children and adolescents with cancer: a systematic review

**DOI:** 10.1186/s12885-023-11353-9

**Published:** 2023-09-14

**Authors:** Theodorus W. Kouwenberg, Elvira C. van Dalen, Elizabeth A. M. Feijen, Stejara A. Netea, Melissa Bolier, Martijn G. Slieker, Firdaus A. A. Mohamed Hoesein, Leontien C. M. Kremer, Heynric B. Grotenhuis, Annelies M. C. Mavinkurve-Groothuis

**Affiliations:** 1 Princess Máxima Center for Pediatric Oncology, Heidelberglaan 25, 3584 CS Utrecht, The Netherlands; 2 Department of Pediatric Cardiology, Wilhelmina Children’s Hospital, Utrecht, The Netherlands; 3 Department of Radiology, Utrecht University Medical Center, Utrecht, The Netherlands

**Keywords:** Cardiotoxicity, Childhood cancer, Echocardiography, Biomarkers, Risk factors

## Abstract

**Background:**

Cardiotoxicity is among the most important adverse effects of childhood cancer treatment. Anthracyclines, mitoxantrone and radiotherapy involving the heart are its main causes. Subclinical cardiac dysfunction may over time progress to clinical heart failure. The majority of previous studies have focused on late-onset cardiotoxicity. In this systematic review, we discuss the prevalence and risk factors for acute and early-onset cardiotoxicity in children and adolescents with cancer treated with anthracyclines, mitoxantrone or radiotherapy involving the heart.

**Methods:**

A literature search was performed within PubMed and reference lists of relevant studies. Studies were eligible if they reported on cardiotoxicity measured by clinical, echocardiographic and biochemical parameters routinely used in clinical practice during or within one year after the start of cancer treatment in ≥ 25 children and adolescents with cancer. Information about study population, treatment, outcomes of diagnostic tests used for cardiotoxicity assessment and risk factors was extracted and risk of bias was assessed.

**Results:**

Our PubMed search yielded 3649 unique publications, 44 of which fulfilled the inclusion criteria. One additional study was identified by scanning the reference lists of relevant studies. In these 45 studies, acute and early-onset cardiotoxicity was studied in 7797 children and adolescents. Definitions of acute and early-onset cardiotoxicity prove to be highly heterogeneous. Prevalence rates varied for different cardiotoxicity definitions: systolic dysfunction (0.0–56.4%), diastolic dysfunction (30.0–100%), combinations of echocardiography and/or clinical parameters (0.0–38.1%), clinical symptoms (0.0–25.5%) and biomarker levels (0.0–37.5%). Shortening fraction and ejection fraction significantly decreased during treatment. Cumulative anthracycline dose proves to be an important risk factor.

**Conclusions:**

Various definitions have been used to describe acute and early-onset cardiotoxicity due to childhood cancer treatment, complicating the establishment of its exact prevalence. Our findings underscore the importance of uniform international guidelines for the monitoring of cardiac function during and shortly after childhood cancer treatment.

**Supplementary Information:**

The online version contains supplementary material available at 10.1186/s12885-023-11353-9.

## Background

In the last few decades, treatment of childhood cancer has improved substantially, with five-year survival rates in high-income countries exceeding 80% [1]. However, childhood cancer survivors (CCS) are subject to adverse effects of cancer treatment, such as cardiotoxicity. Treatment with anthracyclines (e.g., doxorubicin, daunorubicin, epirubicin), anthraquinones (i.e., mitoxantrone) and radiotherapy involving the heart are major causes of cardiotoxicity [2–4]. Through mechanisms not yet fully understood, cardiotoxic changes lead to reduced cardiomyocyte contractility, progressing to myocardial fibrosis, arrhythmia and clinical heart failure [5, 6]. Some of these changes are irreversible: even decades after cancer treatment, morbidity and mortality due to cardiac disease remain significantly higher in CCS compared to the normal population [7, 8].

Cardiotoxicity may be characterized as acute, early-onset or late-onset, which is defined as myocardial damage occurring within a week, within a year, or more than a year after start of treatment, respectively [9]. This subdivision is somewhat arbitrary, and likely represents different stages of damage to cardiomyocytes, myocardial remodeling, partial recovery of cardiomyocytes, and functional adaptation. This process may be accelerated by accumulation of cardiotoxic therapy effects, ageing and lifestyle factors (such as smoking and obesity). The timing of, at first, subclinical cardiac dysfunction and, later on, clinical heart failure differs between individual patients, and is probably subject to risk factors that, at the moment, remain largely unknown [10–12].

The majority of previous studies have focused on late-onset cardiotoxicity and have reported subclinical cardiotoxicity in 0–57% [13] and heart failure in 0–16% [14] of CCS after cancer treatment with anthracyclines. Because of the high risk of late-onset cardiotoxicity, international guidelines (including the International Late Effects of Childhood Cancer Guideline Harmonization Group Cardiomyopathy guideline) recommend life-long echocardiographic screening every three to five years in CCS treated with anthracyclines or radiotherapy involving the heart [15–19]. However, early detection of subclinical cardiac damage in children and adolescents is crucial to initiate treatment at an early stage, provide optimal circumstances for cardiac recovery and remodeling, and hopefully prevent progression of myocardial disease into clinical heart failure [10, 20, 21]. In 1992, the Cardiology Committee of the Children’s Cancer Study Group formulated recommendations for cardiac monitoring of children *during*cancer treatment with anthracyclines [22]. Despite these recommendations, we saw a wide variation in recommendations for cardiac monitoring used in European pediatric oncology protocols, possibly explained by the lack of evidence from clinical research [23]. Until now, systematic reviews on acute and early-onset cardiotoxicity are lacking.

In this systematic review, we evaluated the existing evidence on acute and early-onset cardiotoxicity in children and adolescents (between 0 and 21 years of age, hereafter simply denoted as ‘children’) with cancer treated with anthracyclines, mitoxantrone and/or radiotherapy involving the heart. We report its prevalence according to the different cardiotoxicity definitions in use, as well as the changes of echocardiographic parameters and biomarker levels during and up to one year after childhood cancer treatment. Finally, we assess the evidence on risk factors associated with acute and early-onset cardiotoxicity.

## Methods

### Search strategy

We searched PubMed/Medline with a combination of terms for “children”, “childhood cancer”, “cancer”, “anthracyclines”, “mitoxantrone”, “radiotherapy involving the heart”, “cardiomyopathy/heart failure”, “biomarkers” and “ECG” (Additional file 1). The search was executed until May 12^th^, 2022. In addition, we explored the reference lists of included studies and reviews. Experts in the field provided information on additional studies.

### Study selection

All abstracts were screened by two researchers independently. For studies presumably meeting inclusion criteria, full-text screening was performed by two researchers independently. Disagreements about study inclusion or exclusion were settled by consensus and if that was not possible by third party arbitration.

Studies were included or excluded using an a priori defined study protocol. Criteria for inclusion were: (1) original studies involving at least 25 eligible participants; (2) ≥ 90% of which were younger than 21 years at childhood cancer diagnosis; (3) treated with anthracyclines, mitoxantrone and/or radiotherapy involving the heart; (4) published in English or Dutch from 2000 onwards; (5) that reported *the occurrence of* and/or *risk factors (identified by a multivariate analysis) associated with* acute and early-onset cardiotoxicity measured by clinical, echocardiographic and biochemical parameters routinely used in clinical practice during or within one year after the start of cancer treatment. Both clinical and subclinical abnormalities were considered outcomes of interest: clinical signs and symptoms of heart failure, biomarker levels (troponin (Tn), brain natriuretic peptide (BNP) and is prohormone (proBNP, NT-proBNP) and creatine kinase (CK)) and several left ventricular (LV) echocardiographic parameters (fractional shortening (FS), ejection fraction (EF), global longitudinal strain (GLS), E/A ratio and Tei index). Definitions of cardiotoxicity were used as defined in the included studies.

Criteria for exclusion were as follows: (1) case reports and case series with a description of non-consecutive participants; (2) studies in which the number of children with comorbidity of cardiac disease (like ejection fraction < 50%, valvular heart disease or severe hypertension) at the time of cancer diagnosis exceeded 10%; (3) studies in which the number of children with recurrent malignant disease exceeded 10%; and (4) studies in which the number of children who received cardioprotective interventions (such as dexrazoxane) exceeded 10%. Cut-off values used in the in- and exclusion criteria were chosen with the aim to prevent missing important data whilst not allowing an excess of data that does not pertain to our population of interest.

Studies also including participants that were not eligible for inclusion in this review were only included if separate data were available for eligible participants. From studies reporting on acute and early-onset cardiotoxicity as well as late-onset cardiotoxicity, we only included the results that were obtained no more than one year after start of treatment. For studies with a randomized controlled trial design comparing arms receiving cardioprotective interventions with arms not receiving cardioprotective interventions, the arms not receiving cardioprotective interventions were considered as prospective cohort studies. When multiple studies were published on the same cohort, the study including the largest eligible patient population was included.

### Data extraction

Data extraction was performed by one researcher using a fixed data extraction form and reviewed independently by another researcher. In case of discrepancies that could not be settled by consensus, third-party arbitration was applied.

### Risk of bias assessment

Risk of bias was assessed by one researcher and reviewed independently by another researcher. In case of discrepancies that could not be settled by consensus, third-party arbitration was applied. Assessment was based on previously described checklists according to evidence-based medicine criteria [24, 25] as recommended by Cochrane Childhood Cancer (https://childhoodcancer.cochrane.org/). The different risk of bias criteria are defined in Additional file 2.

### Statistical analysis

We calculated the prevalence rate (hereafter simply denoted as ‘prevalence’) of acute and early-onset cardiotoxicity as the number of children with cancer with acute and/or early-onset cardiotoxicity divided by the total number of children with cancer treated with anthracyclines, mitoxantrone and/or radiotherapy involving the heart. The accompanying 95% confidence interval (CI) was calculated using the Wilson score interval method [26]. We would have performed pooling of results if studies had been sufficiently homogeneous with regard to for example study design, patient and treatment characteristics and outcome definitions; this was not the case and therefore we provide descriptive results.

## Results

### Included studies

**Fig. 1:**
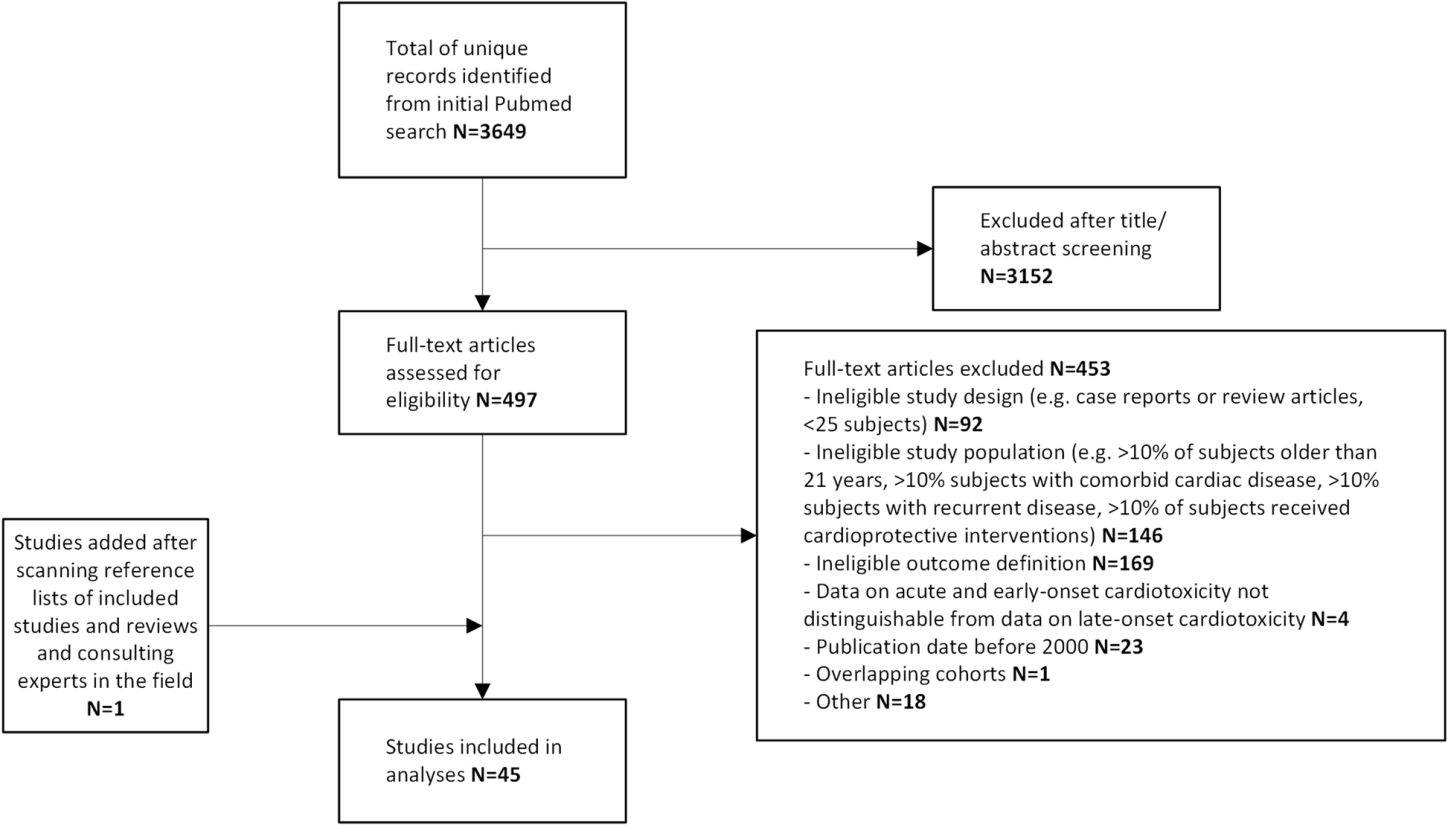
Flowchart of Study Inclusion

 The study selection flowchart is presented in Fig. 1. The search yielded 3649 unique reports, of which 3152 were excluded after title and abstract screening. Full-text screening was performed for the remaining 497 reports, of which 44 fulfilled the inclusion criteria for this review [27–70]. One additional study [71] was identified after scanning the reference lists of the included studies and of review articles addressing our research question. No additional studies were identified by experts in the field, leading to a total number of included studies of 45. Of these studies, 19 described retrospective cohorts and 20 described prospective cohorts. The remaining six studies were randomized controlled trials comparing arms receiving cardioprotective interventions with arms not receiving cardioprotective interventions. A study by Pourier et al. [72] was excluded since the study group was a subset of the larger cohort reported in the included study by Mavinkurve-Groothuis et al. [49] We presume that the publications of Al-Biltagi et al. [28] and El-Shitany et al. [37] comprise the same patient cohort. As the outcomes differed, we decided to present the results of both publications. An overview of the included studies is presented in Table 1, data extraction forms for the included studies are provided in Additional file 3.

**Table 1:** Overview of included studies

Study	Study design^a^	Number of participants	Cancer diagnosis	Age at cancer diagnosis (years)^b^	Timing of cardiotoxicity assessment^d^	Type and cumulative dose of anthracyclines (mg/m^2^)^b,e^	Cumulative dose of mitoxantrone (mg/m^2^)^b^ [number of patients]	Radiotherapy involving the heart (Gy)^b^ [number of patients]	Diagnostic test used for cardiotoxicity assessment
Agha 2016 [27]	S-PC	30	Hematologic malignancies	9.24 ± 4.14	B + /D-/A +	DOX 50-75^c^	nm	nm	Echocardiography
Al-Biltagi 2012 [28]	S-PC	25	Acute lymphoblastic leukemia	9 ± 2.6	B + /D + /A-	DOX 120^c^	0	nm	Echocardiography, biomarker levels
Asselin 2016 [29]	M-RCT	264	Acute lymphoblastic leukemia, lymphoblastic lymphoma	9.7 ± 4.58	B + /D + /A +	DOX 360^c^	nm	nm	Echocardiography, biomarker levels
Berrak 2001 [30]	S-RC	97	Solid tumors	Median 13	B + /D + /A +	DOX 360 ± 98	nm	0	Echocardiography
Brown 2013 [31]	S-RC	71	Ewing sarcoma	11, 2.2–16.1	B + /D + /A +	DOX 365, 113–1,025	nm	Median 50 [6/71 (8.5%)]	Echocardiography
Burke 2021 [61]	M-PC	46	Primary mediastinal large B-cell lymphoma	15.4, 7–17	nm	DOX < 300: 13/46 (28.3%) DOX 300- < 350: 22/49 (44.9%) DOX ≥ 350: 11/46 (23.9%)	0	nm	Echocardiography
Chen 2009 [32]	S-RC	168	Hematologic malignancies, solid tumors	8.1 ± 5.3	B + /D + /A +	DOX 0-360^c^ DNR 0-144^c^ IDA 0-64^c^	20^c^ [9/168 (5.4%)]	22 ± 4.26 [18/168 (10.7%)]	Echocardiography
Cheung 2020	S-PC	39	Acute lymphoblastic leukemia, acute myeloid leukemia	7.5 ± 4.8	B + /D + /A +	nm	nm	nm	Echocardiography, clinical assessment, biomarker levels
Choi 2010 [34]	S-RC	42	Neuroblastoma, peripheral primitive neuro-ectodermal tumor	2.5, 0.3–10.6	B + /D + /A +	DOX 266.1 ± 75.0;294, 87–388	0	Dose nm [3/42 (7.1%)]	Echocardiography
Creutzig 2007 [35]	M-RC	885	Acute myeloid leukemia	nm	B-/D + /A +	ANT 300-450^c^	0-20^c^ [number of patients nm]	nm	Echocardiography, clinical assessment
De Matos Neto 2006 [36]	M-PC	37	Osteosarcoma	Mean 15.4	B + /D + /A +	DOX 345.19 ± 20.3	0	0	Echocardiography
El Amrousy 2022 [62]	S-RCT	30	Acute lymphoblastic leukemia	8.5 ± 1.9	B + /D-/A +	DOX 50^c^ (induction phase, dose in further phases nm)	0	nm	Echocardiography
El-Shitany 2012 [37]	S-RCT	25	Acute lymphoblastic leukemia	9.5 ± 2.6	B + /D-/A +	DOX 120^c^	0	nm	Echocardiography, biomarker levels
Erkus 2007 [38]	S-PC	29	Acute lymphoblastic leukemia	6.65 ± 0.625	B + /D-/A +	ANT 181.6 ± 64.9	0	0	Echocardiography, biomarker levels
Fukumi 2002 [39]	S-PC	29	Hematologic malignancies, solid tumors	Range 0.4–15.2	B-/D + /A-	DOX 120, 50–400 [13/29 (44.8%)] ACLA 120, 50–180 [4/29 (13.8%)] DNR 80, 25–180 [14/29 (48.3%)] EPI 460 [1/29 (3.4%)] PIR 100, 60–360 [13/29 (44.8%)] IDA 49.5, 24–100 [4/29 (13.8%)]	45, 20–120 [9/29 (31.0%)]	0	Echocardiography
Getz 2019 [40]	M-RC	1022	Acute myeloid leukemia	0–1 years: 207/2022 (20.3%) 2–10 years: 354/1022 (34.6%) ≥ 11 years: 461/1022 (45.1%)	B + /D + /A +	DNR 300^c^	48^c^ [number of patients nm]	nm	Echocardiography
Gupta 2018 [41]	S-RCT	40	Acute lymphoblastic leukemia, lymphoma	8.77 ± 2.86	B + /D + /A-	ANT 263.64 ± 80.90	nm	nm	Echocardiography, biomarker levels
Hagag 2019 [42]	S-RCT	40	Acute lymphoblastic leukemia	7.75 ± 3.05; 7.25, 3–13.5	B + /D + /A-	DOX 150^c^	0	nm	Echocardiography, biomarker levels
Hu 2018 (1) [43]	S-RC	36	Hepatoblastoma, rhabdomyosarcoma	3.6 ± 2.2	B-/D-/A +	PIR 293.7 ± 35.4	nm	nm	Clinical assessment
Hu 2018 (2) [44]	S-RC	131	Solid tumors	2, 0.08–12	B-/D-/A +	PIR 154.66 ± 127.04; 120.5, 12–697	40^c^ [5/131 (3.8%)]	0	Echocardiography, clinical assessment, biomarker levels
Ishii 2000 [45]	S-PC	65	nm	nm	nm	ANT < 200: 35/65 (53.8%) ANT ≥ 200: 30/65 (46.2%)	nm	nm	Echocardiography
Kang 2012 [46]	S-RC	123	Hematologic malignancies, solid tumors	6, 0.2–15.0	B + /D + /A-	nm	nm	nm	Echocardiography, clinical assessment
Katzenstein 2022 [63]	M-PC	102	Hepatoblastoma	1.3, 0–15.8	B + /D + /A-	DOX 360‡	0	nm	Echocardiography
Khairat 2019 [47]	M-PC	100	Osteosarcoma	11.85 ± 2.09	B + /D + /A +	DOX 450‡	0	nm	Echocardiography
Kremer 2002 [13]	S-PC	38	Hematologic malignancies, solid tumors	9.9 ± 4.7	B + /D + /A +	ANT 255 ± 118.9	106 ± 13.7 [5/38 (13.2%)]	nm	Echocardiography, biomarker levels
Krischke 2016 [48]	M-PC	101	Hematologic malignancies, solid tumors	5.3, 0.2–17.7	B + /D + /A-	DOX 28.7, 10.4–57.7	nm	nm	Echocardiography
Linares Ballesteros 2021 [64]	S-PC	112	Acute lymphoblastic leukemia, acute myeloid leukemia	6.35, 1.0–17.7	B + /D + /A +	ANT 170-298^c^	nm	nm	Echocardiography, biomarker levels
Mavinkurve-Groothuis 2013 [49]	S-PC	60	Acute lymphoblastic leukemia	Mean 6	B + /D + /A +	ANT 120-300^c^	Range 26.25–52.5 [5/60 (8.3%)]	TBI, dose nm [2/60 (3.3%)]	Echocardiography, biomarker levels
Moke 2018 [50]	S-RC	368	Hematologic malignancies, solid tumors	5.3, 0–18.3	nm	ANT 200, 25–515	nm^f^	19.5, 10–45 [47/368 (12.8%)]	Echocardiography, clinical assessment
Moussa 2017 [51]	S-RC	149	Ewing sarcoma	11, 1–18	B + /D + /A +	DOX 375, 150–375	0	Range 15–55.8 [15/149 (10.1%)]	Echocardiography
Moyo 2021 [65]	S-PC	92	Hematologic malignancies, solid tumors	7.4 ± 3.78	B + /D + /A +	DOX < 100: 33/92 (35.9%) DOX 100–200: 48/92 (52.2%) DOX 200–300: 8/92 (8.7%) DOX > 300: 3/92 (3.3%)	nm	nm	Echocardiography
Oztarhan 2011 [52]	S-PC	276	Acute lymphoblastic leukemia	5.85 ± 2.87	B + /D + /A +	ANT range 30–240	nm	nm	Echocardiography, biomarker levels
Radu 2019 [53]	S-PC	48	Acute lymphoblastic leukemia	1–5 years: 24/48 (50.0%) 6–10 years: 11/48 (22.9%) > 10 years: 13/48 (27.1%)	B + /D + /A +	ANT < 200: 7/48 (14.6%) ANT 200- < 240: 25/48 (52.1%) ANT ≥ 240: 16/48 (33.3%)	nm	nm	Echocardiography, biomarker levels
Sági 2018	M-RC	661	Acute lymphoblastic leukemia, osteosarcoma	6.6 ± 4.3	B + /D + /A +	ANT range 60–840	nm	nm	Echocardiography
Samosir 2021 [66]	S-RC	49	Acute lymphoblastic leukemia	Mean 9.18	nm	DNR median 143.69	nm	nm	Echocardiography
Schramm 2019 [55]	M-RCT	307	Acute lymphoblastic leukemia, lymphoblastic lymphoma	< 10 years: 232/307 (75.6%) > 10 years: 75/307 (24.4%)	nm	DNR: 153/307 (49.8%) DOX: 154/307 (50.2%)	nm	nm	Echocardiography
Shaikh 2013 [59]	S-PC	110	Hematologic malignancies, solid tumors	6.2 ± 3.7; median 5.2	B + /D + /A +	ANT < 100: 40/110 (36.4%) ANT 100–300: 55/110 (50.0%) > 300: 15/110 (13.6%)	nm	nm	Clinical assessment
Stöhr 2006 [57]	M-PC	172	Soft tissue sarcoma	x̄ 8.3 ± 5.5	B + /D + /A +	EPI 450, 150–450 [64/172 (37.2%)] DOX 240, 80–400 [112/172 (65.1%)]	nm	45, 45–51 [10/172 (5.8%)]	Echocardiography, clinical assessment
Tan 2021 [67]	S-RC	458	Hematologic malignancies, solid tumors	M 5.8	nm	ANT median 200	nm^f^	Dose nm [33/458 (7.2%)]	Echocardiography
Tang 2020	S-RC	102	Acute myeloid leukemia	M 4.75, 0.5–15.6	nm	DNR 120^c^	0	nm	Clinical assessment
Tantawy 2011 [58]	S-RC	39	Hodgkin lymphoma	Range 4–14	B-/D + /A-	DOX group A: 75 ± 27.3 [10/39 (25.6%)] DOX group B: 328 ± 64 [29/39 (74.4%)]	nm	0	Echocardiography, clinical assessment
Temming 2011 [59]	S-RC	128	Acute myeloid leukemia	M 2.9, 0.1–12.9	B + /D-/A +	ANT 550-610^cf^	50-122^c^	TBI, dose nm [8/128 (6.3%)]	Echocardiography, clinical assessment
Tringale 2022 [69]	S-RC	50	Hodgkin lymphoma	nm	B-/D-/A +	ANT (dose nm)	nm	4.3, 0–19.1 [47/50 (94.0%)]	Echocardiography
Van Dalen 2006 [23]	S-RC	830	Hematologic malignancies, solid tumors	x̄ 8.8 M 8.7, 0.1–18.0	nm	ANT mean 288; 280, 15–900	Mean 21.8; 12, 12–108 [34/830 (4.1%)]	Dose nm [176/830 (21.2%)]	Clinical assessment
Yu 2021 [70]	S-RC	171	Acute lymphoblastic leukemia	M 5.07, 0.6–14	B + /D + /A-	DNR ≤ 75: 84/171 (49.1%) DNR > 75: 87/171 (50.9%)	nm	nm	Echocardiography

*Abbreviations*: *ACLA* Aclarubicin, *ANT* Anthracycline compound not specified, *DNR* Daunorubicin, *DOX* Doxorubicin, *EPI* Epirubicin, *IDA* Idarubicin, *M* Multicenter, *nm* Not mentioned, *PC* Prospective cohort, *PIR* Pirarubicin, *RC* Retrospective cohort, *RCT* Randomized controlled trial, *S* Single-center, *TBI* Total body irradiation

^a^studies with a randomized controlled trial design compared arms receiving cardioprotective interventions with arms not receiving cardioprotective interventions: for this review we considered arms not receiving cardioprotective interventions as observational studies

^b^unless otherwise specified, mean is provided with standard deviation (x ± y) and median with range (x, y–z)

^c^per protocol dose, actual received dose not reported

^d^B, before cardiotoxic cancer treatment; D, during cardiotoxic cancer treatment; A, after cardiotoxic cancer treatment (‘ + ’ indicating ‘assessment performed’, ‘- ‘ indicating ‘assessment not performed’)

^e^all patients in all studies received anthracycline compounds; ^f^mitoxantrone dose converted to anthracycline equivalent, not reported separately

In total, 7797 subjects in 45 studies were eligible for this review, with the number of participants per study ranging from 25 to 1022. An overview of the oncologic diagnoses is provided in Additional file 4. Age at cancer diagnosis ranged from 0 to 19.6 years. Mean and median age at cancer diagnosis ranged from 3.6 to 15.4 years and from 2 to 15.4 years, respectively. Six studies did not provide exact information on age.

Follow-up duration was reported in 15 publications [29–32, 40, 50, 51, 57, 59–61, 63, 67–69], either as mean, median or range. In these studies, follow-up duration ranged from 0.0 to 28.4 years; mean and median follow-up duration ranged from 5.6 to 8.5 years and from 1.7 to 8.9 years, respectively. Baseline (before treatment with anthracyclines, mitoxantrone and/or radiotherapy involving the heart) was defined as the start of follow-up in 30 publications [27, 28, 30–42, 46–49, 51–54, 57, 61, 62, 64, 65, 70, 71]. Two publications defined the first dose of anthracyclines as the start of follow-up [56, 60], two other publications defined the end of therapy as the start of follow-up [50, 69]. The start of follow-up was not defined in the remaining 11 publications [29, 43–45, 55, 58, 59, 63, 66–68]. In the following sections, we have reported only results obtained no more than one year after the start of treatment.

All participants of all studies received anthracyclines. As reported in Table 1, different anthracycline compounds and sometimes combinations of anthracycline compounds were used. Actual received cumulative doses of anthracyclines were reported in 21 studies, including 3960 children, either as mean, median or range. Of these 21 studies, 13 studies reported cumulative doses of specific anthracycline compounds. Three studies did not specify which anthracycline compounds were administered but provided equivalence ratios for the reported anthracycline doses; five studies reported anthracycline doses without providing equivalence ratios. Cumulative doses ranged from 10.4 to 1025 mg/m^2^ (mean 28.7 to 365 mg/m^2^, median 120.5 to 375 mg/m^2^).

In 11 studies, mitoxantrone administration was reported; 12 studies reported that no mitoxantrone had been administered. Actual received cumulative doses were reported separately in four studies, including 53 children treated with mitoxantrone, either as mean, median or range. Cumulative doses ranged from 12 to 120 mg/m^2^ (mean 21.8 to 106 mg/m^2^, median 12 to 45 mg/m^2^).

Radiotherapy involving the heart was reported in 11 studies; six studies reported that no patients had received radiotherapy involving the heart. Six studies, including 143 children treated with radiotherapy involving the heart, reported on dosing. Doses ranged from 0 to 55.8 Gy (median 4.3 to 50 Gy). The reported doses refer to the whole radiation field, only one of the studies specifically reported on heart dosimetry [69].

### Risk of bias in included studies

Scores for the different risk of bias categories per study are summarized in Additional file 5; see Additional file 3 for substantiation of the assessment. With regards to internal validity, the risk of selection bias was low in eight studies (17.8%), high in ten studies (22.2%), and unclear in 27 studies (60.0%), as the original cohort from which the study group was selected was often not described. The risk of attrition bias for all diagnostic tests used for cardiotoxicity assessment was low in 21 studies (46.7%), high in ten studies (22.2%), and unclear in eight studies (17.8%). In the remaining six studies (13.3%), the risk of attrition bias was either different for the separate diagnostic tests used, or different for the same test at separate time points (see Additional file 3). The risk of detection bias in the 41 studies describing echocardiography was low in seven studies (17.1%), high in one study (2.4%), and unclear in the remaining 33 (80.5%). The risk of detection bias was considered low for biomarker levels, as blinding was deemed irrelevant for outcomes diagnosed by laboratory tests. In the 12 studies describing clinical assessment, only one study (8.3%) reported blinding of outcome assessors, therefore, the risk of detection bias was unclear in the remaining 11 studies (91.7%). Only three studies (6.7%) performed a multivariate analysis focused on risk factors for acute and early-onset cardiotoxicity, but as several prognostic factors for cardiac function were not taken into account, the risk of confounding was regarded high in all three studies.

Regarding external validity, the study group was well-defined in 18 studies (40.0%) and not well-defined in 27 studies (60.0%); the main reason being the reporting of per protocol rather than actual received cumulative anthracycline doses. Follow-up was well-defined in 20 studies (44.4%) and not well-defined in 25 studies (55.6%) as often the duration of follow-up was not reported and the starting and end points of follow-up were unclear. The outcome was well-defined in 28 studies (62.2%) – meaning a definition of abnormal outcome was provided for all of the performed diagnostic tests – and not well-defined in 17 studies (37.8%). In all three studies that performed a multivariate risk factor analysis, a clear definition of risk estimation analysis was provided.

### Reporting of cardiotoxicity in included studies

The included studies reported cardiotoxicity based on echocardiography, clinical symptoms, biomarker levels or combinations of these modalities. Most studies did not separate acute from early-onset cardiotoxicity. Pooling of results was not feasible, due to the heterogeneity among the included studies regarding treatment, age at diagnosis, and the cardiotoxicity definitions that were used. Reporting of cardiotoxicity proved to be highly heterogeneous. In 20 studies [30–32, 34, 35, 39, 40, 50, 51, 55, 59–61, 63, 65–70], only dichotomous outcomes were reported, i.e. a definition of cardiotoxicity was provided with a prevalence. In seven studies [28, 37, 44, 47, 53, 54, 62], only continuous results were reported, i.e. results of diagnostic tests were provided without a definition of abnormal values. The remaining 18 studies [27, 29, 33, 36, 38, 41–43, 45, 46, 48, 49, 52, 56–58, 64, 71] reported dichotomous as well as continuous results.

### Prevalence of cardiotoxicity

An overview of the prevalence of cardiotoxicity for all used outcome definitions, as well as the timing of onset, is presented in Table 2.

**Table 2:** Prevalence of acute and early-onset cardiotoxicity in children with cancer treated with anthracyclines, mitoxantrone and/or radiotherapy involving the heart

Study	Definition of cardiotoxicity	Timing of onset^a^	Prevalence (n/N)	Prevalence (%; 95% CI)
*Echocardiography – systolic dysfunction*				
Fractional shortening				
Chen 2009 [32]	< 28%	Acute/early	2/168	1.2; 0.3–4.2
Mavinkurve-Groothuis 2013 [49]		Early	0/60	0.0; 0.0–6.0
Tan 2021 [67]		Acute/early	15/458	3.3; 2.0–5.3
Agha 2016 [27]		Acute/early	2/30	6.7; 1.8–21.3
	Decrease > 10% of baseline		12/30	40.0; 24.6–57.7
De Matos Neto 2006 [36]	≤ 29% or decrease ≥ 10% of baseline	Acute/early	7/37	18.9; 9.5–34.2
Kremer 2002 [13]	< 30% or decrease ≥ 15% of baseline	Early	7/32	21.9; 11.0–38.8
Krischke 2016 [48]	Once < 28%	Acute/early	6/101	5.9; 2.8–12.4
	Once or more ≤ 30%		20/101	19.8; 13.2–28.6
Ejection fraction				
Fukumi 2002 [39]	< 50%	Acute/early	0/29	0.0; 0.0–11.7
Hagag 2019 [42]		Acute/early	0/40	0.0; 0.0–8.8
Linares Ballesteros 2021 [64]	< 53%	Acute	0/112	0.0; 0.0–3.3
		Early	20/112	17.9; 11.9–26.0
Moyo 2021 [65]	Decrease ≥ 10% to a final value of < 50%	Acute/early	0/92	0.0; 0.0–4.0
Samosir 2021 [66]	< 50% or decrease > 10% of baseline	Acute/early	5/49	10.2; 4.4–21.8
Brown 2013 [31]	Decrease > 10% of baseline	Acute/early	10/71	14.1; 7.8–24.0
Berrak 2001 [30]	Decrease > 15% of baseline	Early	1/97	1.0; 0.2–5.6
Gupta 2018 [41]	Decrease ≥ 20% of baseline	Early	3/40	7.5; 2.6–19.9
Fractional shortening or ejection fraction				
Erkus 2007 [38]	FS < 29% or EF < 55%	Early	0/29	0.0; 0.0–11.7
Moussa 2017 [51]	FS ≤ 28% or EF ≤ 58%	Acute/early	31/149	20.8; 15.1–28.0
Global longitudinal strain				
Cheung 2020	Decrease ≥ 20%	Early	22/39	56.4; 41.0–70.7
*Echocardiography – diastolic dysfunction*				
Tei index				
Ishii 2000 [45]	> 0.35	Acute/early	ANT < 200 mg/m2: 9/30	30.0; 16.7–47.9
			ANT ≥ 200/ < 400 mg/m2: 25/30	83.3; 66.4–92.7
			ANT ≥ 400 mg/m2: 12/12	100; 75.8–100
E/A ratio				
Oztarhan 2011 [52]	< 1	Acute/early	91/251	36.3; 30.6–42.4
*Combination of echocardiographic and/or clinical parameters*				
National Cancer Institute – Common Terminology Criteria for Adverse Events				
Creutzig 2007 [35]	Version nm	Acute/early	LVSD/FS, grade 1: 11/885	1.2; 0.7–2.2
			LVSD/FS, grade 2: 9/885	1.0; 0.5–1.9
Asselin 2016 [29]	Version 2.0	Acute/early	LVSD/FS, grade 3 or 4: 3/624	0.5; 0.2–1.4
Schramm 2019 [55]		Acute/early	LVSD/EF, grade 1: 3/229	1.3; 0.4–3.8
			LVSD/EF, grade 3: 2/229	0.9; 0.2–3.1
			LVSD/FS, grade 1: 3/175	1.7; 0.6–4.9
Getz 2019 [40]	Version 3.0	Acute/early	LVSD/FS or EF, grade 2 or higher: 118/795	14.8; 12.5–17.5
Brown 2013 [31]	Version 3.0/4.0	Acute/early	LVSD/EF, grade 1: 25/71	35.2; 25.1–46.8
			LVSD/EF, grade 2: 10/71^b^	14.1; 7.8–24.0
			LVSD/EF, grade 3: 5/71	7.0; 3.0–15.4
Katzenstein 2022 [63]		Acute/early	LVSD, grade 3 or higher: 1/102	1.0; 0.2–5.3
			RVD, grade 3 or higher: 1/102	1.0; 0.2–5.3
			Cardiopulmonary arrest, grade 4: 2/102	2.0; 0.5–6.9
Burke 2021 [61]	Version 4.0	Acute/early	LVSD/FS or EF, grade 2: 1/46	2.2; 0.4–11.3
Tringale 2022 [69]	Version 5.0	Acute/early	Grade 2 ‘left-ventricular strain’: 1/50	2.0; 0.4–10.5
Yu 2021 [70]		Acute/early	LVSD/EF: 0/171^c^	0.0; 0.0–2.2
Combination of echocardiographic parameters				
Choi 2010 [34]	FS < 28% or increased ventricular diastolic or systolic diameter for age	Acute/early	16/42	38.1; 25.0–53.2
Combination of echocardiographic and clinical parameters				
Cheung 2020	Clinical heart failure or reduced EF	Acute	0/39	0.0; 0.0–9.0
		Early	0/39	0.0; 0.0–9.0
Hu 2018 (2) [44]	EF < 53% and heart failure symptoms	Early	2/131	1.5; 0.4–5.4
Kang 2012 [46]	EF < 45% or evidence of clinical congestive heart failure	Acute/early	9/123	7.3; 3.9–13.3
Moke 2018 [50]	FS < 25% or decrease of > 10% of baseline; EF < 50% or decrease of > 10% of baseline; ventricular dilation; cardiomyopathy; E/A reversal; valve abnormality or arrhythmia leading to cardiology referral or cardiac medication initiation	Early	8/368	2.2; 1.1–4.2
Stöhr 2006 [57]	FS < 29% without clinical symptoms (subclinical cardiomyopathy)	Acute/early	2/172	1.2; 0.3–4.1
	FS < 29% with clinical symptoms (clinical cardiomyopathy)		1/172	0.6; 0.1–3.2
Tantawy 2011 [58]	EF < 50%	Acute/early	12/39	30.8; 18.6–46.4
	Clinical heart failure		0/39	0.0; 0.0–9.0
Temming 2011 [59]	FS < 28% without clinical symptoms (subclinical cardiomyopathy)	Acute/early	6/95	6.3; 2.9–13.1
	FS < 28% with clinical symptoms (clinical cardiomyopathy)		7/95	7.4; 3.6–14.4
*Clinical symptoms*				
Creutzig 2007 [35]	Clinical signs and symptoms of cardiomyopathy not attributable to other known causes such as sepsis or renal failure	Acute/early	14/885	1.6; 0.9–2.6
Hu 2018 (1) [43]	Heart failure	Early	0/36	0.0; 0.0–9.6
Shaikh 2013 [59]	Cardiac dysfunction	Acute/early	28/110	25.5; 18.2–34.3
Tang 2020	Chemotherapy-related severe myocardial damage or impaired cardiac function	Acute/early	0/102	0.0; 0.0–3.6
Van Dalen 2006 [23]	Congestive heart failure defined as the presence of following clinical signs: dyspnea, pulmonary oedema, peripheral oedema and/or exercise intolerance, which were treated with anticongestive therapy	Acute/early	16/830	1.9; 1.2–3.1
*Biomarker levels*				
Gupta 2018 [41]	proBNP ≥ 100 pg/mL	Early	15/40	37.5; 24.2–53.0
Linares Ballesteros 2021 [64]	BNP > 100 pg/mL	Early	26/112	23.2; 16.4–31.8
	TnI > 0.05 ng/L		0/112	0.0; 0.0–3.3
	TnT > 0.04 ng/mL		0/112	0.0; 0.0–3.3
Mavinkurve-Groothuis 2013 [49]	NT-proBNP > 97.5th percentile	Early	8/41	19.5; 10.2–34.0
	cTnT > 0.01 ng/mL		1/41	2.4; 0.4–12.6
Erkus 2007 [38]	cTnI > 0.04 ng/mL	Early	2/29	6.9; 1.9–22.0
Asselin 2016 [29]	cTnT > 0.01 ng/mL	Acute/early	10/114	8.8; 4.8–15.4
Kremer 2002 [13]	cTnT > 0.040 ng/mL	Acute	3/38	7.9; 2.7–20.8
		Early	1/31	3.2; 0.6–16.2

*Abbreviations*: *ANT* Cumulative anthracycline dose, *BNP* Brain natriuretic peptide, *CI* Confidence interval, *cTnI* Cardiac troponin I, *cTnT* Cardiac troponin T, *EF* Ejection fraction, *FS* Fractional shortening, *LVSD* Left ventricular systolic dysfunction, *NCI CTCAE* National Cancer Institute – Common Terminology Criteria for Adverse Events, *NT-proBNP* N-terminal prohormone of brain natriuretic peptide, *proBNP* Prohormone of brain natriuretic peptide, *RVD* Right ventricular dysfunction, *TnI* Troponin I, *TnT* Troponin T

^a^acute (within one week after a treatment); early (within one year after start of treatment); acute/early (acute and early not separable)

^b^ an additional 10/71 (14.1%; 95% CI 7.8–24.0) had 10–19% decrease from baseline ejection fraction (version 4.0, grade 2)

^c^31/171 (18.1%; 95% CI 13.1–24.6) showed echo abnormalities including pericardial effusion (*N* = 14), left ventricular hypertrophy (*N* = 11), widened pulmonary artery (*N* = 5) and valve disease (*N* = 5), not graded according to NCI CTCAE

### Echocardiography – systolic dysfunction

For echocardiographic outcomes, the definition of abnormal was based either on various absolute values, or decreases relative to the baseline, or on combinations of both.

In seven studies including 886 patients, with the number of patients per study ranging from 30 to 458 patients, cardiotoxicity was defined as a decrease in LV FS. The prevalence of abnormal FS ranged from 0.0 to 40.0%.

Another eight studies including 530 patients (29 to 112 patients per study) defined cardiotoxicity as a decrease in LV EF. Abnormal values were found in 0.0 to 17.9% of patients.

Two studies including 178 patients (29 and 149 patients, respectively) used cardiotoxicity definitions combining absolute values of FS and EF; reported prevalences were 0.0% and 20.8%, respectively.

Only one study, including 39 patients, considered GLS in defining cardiotoxicity. Abnormal values were found in 56.4% of the study group.

### Echocardiography – diastolic dysfunction

Two studies based their definition of cardiotoxicity on diastolic parameters. One study including 251 patients defined cardiotoxicity as an E/A ratio < 1, and described a prevalence of 36.3%. Another study including 30 patients considered a Tei index of > 0.35 to be indicative of diastolic dysfunction. The prevalence of diastolic dysfunction in this cohort ranged from 30.0% in patients who received < 200 mg/m^2^ of anthracyclines to 100% in patients who received ≥ 400 mg/m^2^.

### Combination of echocardiographic and/or clinical parameters

A total of nine studies including 2973 patients (46 to 885 patients per study) used different versions of the National Cancer Institute – Common Terminology Criteria for Adverse Events (NCI CTCAE) to define cardiotoxicity. Prevalence of cardiotoxicity ranged from 0.0 to 35.2%.

One study including 42 patients defined cardiotoxicity as FS < 28% or increased LV diastolic or systolic diameter for age, and found a prevalence of 38.1%.

In seven studies, including 967 patients (39 to 368 patients per study), the definition of cardiotoxicity was based on both abnormal echocardiography parameters and clinical symptoms. Between these studies, the echocardiographic parameters varied highly and included FS, EF, ventricular dilation and E/A reversal. Clinical symptoms were, by some authors, further elaborated as ‘clinical heart failure’, ‘heart failure symptoms’, ‘evidence of clinical congestive heart failure’ and ‘cardiomyopathy, valve abnormality or arrhythmia leading to cardiology referral or cardiac medication initiation’. Prevalence of cardiotoxicity in these studies ranged from 0.0 to 30.8%.

### Clinical symptoms

In five studies, including 1963 patients (36 to 885 patients per study), cardiotoxicity was based only on clinical symptoms. Of these studies, four did not further define those symptoms. Prevalence of cardiotoxicity ranged from 0.0% to 25.5%. In the final study, including 830 patients, congestive heart failure was defined as ‘the presence of following clinical signs: dyspnea, pulmonary oedema, peripheral oedema and/or exercise intolerance, which were treated with anticongestive therapy’. A prevalence of 1.9% was found.

### Biomarker levels

Six studies including 374 patients (29 to 114 patients per study) defined cardiotoxicity by means of various elevated biomarker levels. In four studies, including 305 patients, (cardiac) troponin T was used. Abnormal results were found in 0.0 to 8.8% of participants.

In two studies, including 141 patients, (cardiac) troponin I was measured. The prevalence of abnormal results was 6.9 and 0.0%, respectively.

Three studies, including 193 patients, defined cardiotoxicity based on brain natriuretic peptide and its prohormone; the prevalence of abnormal results ranged from 19.5 to 37.5%.

### Echocardiography – continuous results

See Table 3 for a detailed overview of continuous echocardiographic outcomes before and after cardiotoxic childhood cancer treatment.

**Table 3:** Left ventricular echocardiographic parameters before and after therapy in children with cancer treated with anthracyclines, mitoxantrone and/or radiotherapy involving the heart

Study	Number of participants	Parameter value before therapy^a^	Timing of measurement after therapy^b^	Parameter value after therapy^a^	*P* value
Fractional shortening (%)					
Agha 2016 [27]	30	38.70 ± 3.93	Acute/early	36.00 ± 5.00	< 0.01
Al-Biltagi 2012 [28]	25	40 ± 4.87	Acute	33.5 ± 6.58	0.02
De Matos Neto 2006 [36]	37	37.03 ± 3.66	Acute/early	35.26 ± 3.91	nm
El Amrousy 2022 [62]	30	40.7 ± 5.2	Acute/early	36 ± 3.8	nm
El-Shitany 2012 [37]	25	40 ± 4.62	Acute/early	33.5 ± 6.24	< 0.05
Erkus 2007 [38]	29	39.6 ± 2.9	Early	36.6 ± 2.9	< 0.05
Hagag 2019 [42]	40	35.6 ± 1.93	Acute/early	29.35 ± 1.63	< 0.001
		36, 32–38		29, 27–33	nm
Hu 2018 (2) [44]	131	nm	Acute/early	Cumulative PIR dose < 100 mg/m^2^: 37.22 ± 3.56	-
				Cumulative PIR dose ≥ 100/ < 200 mg/m^2^: 37.37 ± 5.01	0.874 ‡
				Cumulative PIR dose ≥ 200 mg/m^2^: 36.54 ± 4.8	0.002 ‡
Kang 2012 [46]	123	41.0 ± 5.2	Acute/early	Cumulative ANT dose 100 mg/m^2^: 38.6 ± 6.2	nm
				Cumulative ANT dose 200 mg/m^2^: 37.2 ± 4.8	nm
				Cumulative ANT dose 300 mg/m^2^: 35.2 ± 6.0	nm
				Cumulative ANT dose 400 mg/m^2^: 34.2 ± 6.5	nm
				Cumulative ANT dose 500 mg/m^2^: 33.5 ± 8.0	nm
Kremer 2002 [13]	38	40.5 ± 3.7	Acute/early	36.4 ± 4.6	nm
Krischke 2016 [48]	101	39 ± 8.1	Early	37.6 ± 6.6	nm
Linares Ballesteros 2021 [64]	112	ALL SR: 34.5, 19–43	Acute/early	ALL SR: 33.0, 31–40	nm
		ALL IR: 37.0, 27–46		ALL IR: 34.1, 22–42	nm
		ALL HR: 36.0, 23–46		ALL HR: 34.5, 25–43	nm
		AML: 37.0, 34–48		AML: 34.0, 21–42	nm
Mavinkurve-Groothuis 2013 [49]	60	40 ± 5	Early	35 ± 3	< 0.0001
Oztarhan 2011 [52]	276	43.19 ± 4.19	Acute/early	Cumulative ANT dose 30-90 mg/m^2^: 43.19 ± 5.28	nm
				Cumulative ANT dose 120-180 mg/m^2^: 41.31 ± 7.01	nm
				Cumulative ANT dose ≥ 210 mg/m^2^: 40.15 ± 6.11	nm
Sági 2018	661	41.4 ± 6.0	Acute/early	40.4 ± 6.1	nm
Shaikh 2013 [59]	110	36.6 ± 2.6	Acute/early	32.9 ± 5.0	< 0.001
Stöhr 2006 [57]	172	nm	Acute/early	35.6 ± 4.9	nm
Tantawy 2011 [58]	39	nm	Acute/early	Cumulative DOX dose 50-100 mg/m^2^: 41.5 ± 8.8	-
				Cumulative DOX dose 210–485 mg/m^2^: 40 ± 4	0.562 ‡
Fractional shortening (z-score)					
Asselin 2016 [29]	264	0.37	Acute/early	-1.68	nm
Ejection fraction (%)					
Agha 2016 [27]	30	70.60 ± 5.70	Acute/early	66.00 ± 7.18	< 0.01
Erkus 2007 [38]	29	75.20 ± 0.90	Early	68.4 ± 4.8	< 0.05
Gupta 2018 [41]	40	64.85 ± 4.94	Acute/early	56.15 ± 4.79	nm
Hagag 2019 [42]	40	68.25 ± 3.91	Acute/early	54.9 ± 5.35	< 0.001
		67, 62–74		53, 50–69	nm
Hu 2018 (1) [43]	36	nm	Acute/early	Healthy controls: 66.6 ± 3.4	-
				Patients: 65.7 ± 5.1	0.52 §
Hu 2018 (2) [44]	131	nm	Acute/early	Cumulative PIR dose < 100 mg/m^2^: 68.88 ± 6.79	-
				Cumulative PIR dose 100-200 mg/m^2^: 69.35 ± 2.73	0.689 ‡
				Cumulative PIR dose ≥ 200 mg/m^2^: 65.95 ± 7.94	0.034 ‡
Kang 2012 [46]	123	72.7 ± 5.9	Acute/early	Cumulative ANT dose 100 mg/m^2^: 69.3 ± 7.8	nm
				Cumulative ANT dose 200 mg/m^2^: 67.7 ± 5.7	nm
				Cumulative ANT dose 300 mg/m^2^: 65.8 ± 8.5	nm
				Cumulative ANT dose 400 mg/m^2^: 63.7 ± 8.6	nm
				Cumulative ANT dose 500 mg/m^2^: 63.0 ± 10.6	nm
Khairat 2019 [47]	100	67.7 ± 3.62	Acute/early	Normal RV GLS: 64.31 ± 7.31	0.697 ¶
				Decreased RV GLS: 65.75 ± 1.50	
Linares Ballesteros 2021 [64]	112	ALL SR (Teichholz method): 65.5, 40–74	Acute/early	ALL SR (Teichholz method): 63.0, 60–72	nm
		ALL SR (Simpson method): 60.2, 38–65		ALL SR (Simpson method): 59.0, 55.7–67.2	nm
		ALL IR (Teichholz method): 68.5, 54–78		ALL IR (Teichholz method): 64.5, 46–74	nm
		ALL IR (Simpson method): 62.9, 53.8–74.0		ALL IR (Simpson method): 62.1, 48–75	nm
		ALL HR (Teichholz method): 66.0, 48–77		ALL HR (Teichholz method): 64.5, 50–75	nm
		ALL HR (Simpson method): 63.5, 46–70		ALL HR (Simpson method): 64.7, 53–67	nm
		AML (Teichholz method): 68.0, 64–77		AML (Teichholz method): 63.0, 43–73	nm
		AML (Simpson method): 62.2, 55.5–74.3		AML (Simpson method): 59.9, 41.0–64.4	nm
Oztarhan 2011 [52]	276	81.90 ± 4.13	Acute/early	Cumulative ANT dose 30-90 mg/m^2^: 81.11 ± 5.51	nm
				Cumulative ANT dose 120-180 mg/m^2^: 78.48 ± 6.88	nm
				Cumulative ANT dose ≥ 210 mg/m^2^: 75.97 ± 6.69	nm
Radu 2019 [53]	48	Median 63, IQR 60.5–65	Acute/early	Median 62, IQR 60–65	0.833
Shaikh 2013 [59]	110	69.9 ± 4.3	Acute/early	62.6 ± 9.6	< 0.001
Tantawy 2011 [58]	39	nm	Acute/early	Cumulative DOX dose 50-100 mg/m^2^: 58.7 ± 7.3	-
				Cumulative DOX dose 210–485 mg/m^2^: 52 ± 4.4	0.043 ‡
Global longitudinal strain (%)					
Agha 2016 [27]	30	-21.58 ± 2.54	Acute/early	-19.18 ± 3.59	0.001
Al-Biltagi 2012 [28]	25	-18.65 ± 4.52	Acute	-15.10 ± 2.45	0.04
Cheung 2020	39	-17.9 ± 1.6	Acute/early	-15.4 ± 1.7	nm
El Amrousy 2022 [62]	30	-19.8 ± 1.2	Acute/early	-15.8 ± 1.6	< 0.05
El-Shitany 2012 [37]	25	-18.65 ± 2.9	Acute/early	-15.1 ± 1.769	< 0.05
Hu 2018 (1) [43]	36	nm	Acute/early	Healthy controls: -22.2 ± 1.9	-
				Patients: -17.9 ± 1.9	< 0.01 §
Khairat 2019 [47]	100	-23.77 ± 0.93	Acute/early	Normal RV GLS: LV GLS 23.84 ± 0.88	0.339 ¶
				Decreased RV GLS: LV GLS 23.40 ± 1.61	
Linares Ballesteros 2021 [64]	112	ALL SR: -23.4, -26.6–16.8	Acute/early	ALL SR: -23.2, -31–19	nm
		ALL IR: -24.4, -30–18		ALL IR: -22.7, -28.0–17.4	nm
		ALL HR: -22.0, -30–18		ALL HR: M -24.2,-26.3–18.2	nm
		AML: -21, -30–15		AML: -22.6, -26.1–16.1	nm
Mavinkurve-Groothuis 2013 [49]	60	-18.2 ± 3.1	Early	-16.7 ± 5.2	0.5
E/A ratio					
Agha 2016 [27]	30	1.29 ± 0.27	Acute/early	1.03 ± 0.37	< 0.01
Al-Biltagi 2012 [28]	25	1.60 ± 0.42	Acute	1.5 ± 0.37	nm
El Amrousy 2022 [62]	30	1.49 ± 1.4	Acute/early	1.45 ± 1.6	nm
El-Shitany 2012 [37]	25	1.904 ± 0.403	Acute/early	1.966 ± 0.389	nm
Hagag 2019 [42]	40	1.31 ± 0.16	Acute/early	1.28 ± 0.04	0.368
		1.3, 1.1–1.6		1.3, 1–1.3	nm
Mavinkurve-Groothuis 2013 [49]	60	1.8 ± 0.6	Early	1.8 ± 0.6	0.8
Oztarhan 2011 [52]	276	1.34 ± 0.28	Acute/early	Cumulative ANT dose 30-90 mg/m^2^: 1.23 ± 0.27	nm
				Cumulative ANT dose 120-180 mg/m^2^: 1.21 ± 0.23	nm
				Cumulative ANT dose ≥ 210 mg/m^2^: 1.21 ± 0.28	nm
Radu 2019 [53]	48	Median 1.5, IQR 1.2–1.9	Acute/early	Median 1.4, IQR 1.2–1.9	0.031
Shaikh 2013 [59]	110	1.6 ± 1.8	Acute/early	1.3 ± 0.33	< 0.001
Tantawy 2011 [58]	39	nm	Acute/early	Cumulative DOX dose 50-100 mg/m^2^: 1.7 ± 0.4	-
				Cumulative DOX dose 210–485 mg/m^2^: 1.7 ± 0.5	0.907 ‡
Tei index					
Agha 2016 [27]	30	0.32 ± 0.06	Acute/early	0.36 ± 0.08	< 0.01
Ishii 2000 [45]	65	nm	Acute/early	Healthy controls: 0.33 ± 0.02	-
				Cumulative ANT dose < 200 mg/m^2^: 0.34 ± 0.09	-
				Cumulative ANT dose ≥ 200 mg/m^2^: 0.45 ± 0.06	< 0.05 ‡ §
Shaikh 2013 [59]	110	0.3 ± 0.05	Acute/early	0.4 ± 0.07	< 0.001

*Abbreviations ALL* Acute lymphoblastic leukemia, *AML* Acute myeloid leukemia, *ANT* Anthracycline, *GLS* Global longitudinal strain, *HR* High risk, *IQR* Interquartile range, *IR* Intermediate risk, *LV* Left ventricle, *nm* Not mentioned, *PIR* Pirarubicin, *RV* Right ventricle, *SR* Standard risk

^a^unless otherwise specified, mean is provided with standard deviation (x ± y) and median with range (x, y–z)

^b^acute (within one week after a treatment); early (within one year after start of treatment); acute/early (acute and early not separable)

^‡^ *P* values in comparison to the lowest dose group are presented

^§^ *P* values in comparison to healthy controls are presented

^¶^ *P* values comparing subgroups with normal and decreased right ventricular global longitudinal strain are presented

### Fractional shortening

A total of 19 studies presented FS values. In 13 studies comparing FS before and up to a year after cardiotoxic cancer treatment, including 1298 patients (25 to 661 patients per study), mean baseline values varied between 34.5 and 41.4%, whilst mean post-treatment values varied between 29.4 and 40.4%. All studies found significantly lower post-treatment than baseline FS values. One study in 172 patients reported a mean post-treatment FS of 35.6%, but did not report baseline values.

Four studies including 569 patients (39 to 276 patients per study) reported on FS in relation to received cumulative anthracycline dose. These studies all found lower FS in patients who received higher cumulative anthracycline doses, although the difference was not always significant.

One last study including 264 patients did not report absolute FS values, but rather expressed measurements as z scores in relation to measurements in 285 healthy children. They found a z score of 0.37 at baseline, and a z score of -1.68 at the end of doxorubicin treatment. The authors did not report on the significance of the change.

### Ejection fraction

Thirteen studies reported on EF values. Mean EF before and up to a year after cardiotoxic cancer treatment were compared in six studies, including 361 patients (29 to 112 patients per study). Mean baseline values varied between 60.2 and 75.2%, mean post-treatment values varied between 54.9 and 68.4%. Four studies tested the significance of these differences and all found significantly lower post-treatment than baseline EF values.

One study including 36 patients compared mean EF after cardiotoxic cancer treatment (65.7%) with mean EF in healthy controls (66.6%); the difference was not significant.

One study including 48 patients reported a median EF value of 63% at baseline and 62% at one year after diagnosis; the difference was not significant.

Four studies, including 569 patients (39 to 276 patients per study), related EF values to received cumulative anthracycline dose. Patients who received higher doses of anthracyclines had lower EF values in all studies, although the difference was not always significant.

Finally, a study in 100 patients reported a mean EF of 67.7% at baseline, and reported mean EF values separately for subgroups with normal and decreased right ventricular GLS (64.3 and 65.8%, respectively). The difference between both subgroups was not statistically significant.

### Global longitudinal strain

In nine studies, GLS was measured. Seven publications, including 321 patients (25 to 112 patients per study), reported mean values before and up to one year after cardiotoxic cancer treatment. In those studies, mean baseline values varied between -17.9 and -24.4%, mean post-treatment values varied between -15.1 and -24.2%. All studies found lower post-treatment than baseline GLS values. Significance of these differences was found in some, but not all studies.

One study including 36 patients compared mean GLS after cardiotoxic cancer treatment (-17.9%) with mean GLS in healthy controls (-22.2%), the difference was statistically significant.

A final study, including 100 patients, found a mean LV GLS of -23.8% at baseline, and reported mean LV GLS values separately for subgroups with normal and decreased right ventricular GLS (-23.8 and -23.4%, respectively). The difference between both subgroups was not statistically significant.

### E/A ratio

E/A ratio was measured in ten studies. Seven studies including 320 patients (25 to 110 patients per study) compared mean E/A ratios at baseline (ranging between 1.29 and 1.904) and post-treatment (ranging between 1.03 and 1.966). When compared with baseline measurements, mean post-treatment E/A ratios were decreased in five studies, the same in one study and increased in the final study. Significance of these differences was found in some, but not all studies.

In one study including 48 patients, the median E/A ratio was 1.5 at baseline and 1.4 at one year after diagnosis; the difference was statistically significant.

One study, including 276 patients, reported mean E/A ratio at baseline and in subgroups of patients who received 30 to 90, 120 to 180 and ≥ 210 mg/m^2^ of anthracyclines, they found progressively decreasing values from 1.34 to 1.21; whether these changes were significant was not reported. A final study, including 39 patients, reported a mean E/A ratio of 1.7 both in patients who received 50 to 100 mg/m^2^ and in patients who received 210 to 485 mg/m^2^ of anthracyclines.

### Tei index

Three studies reported on Tei index. Two studies, including 140 patients (30 and 110 patients, respectively), compared mean Tei index at baseline (0.32 and 0.3, respectively) and post-treatment (0.36 and 0.4, respectively). Both studies found the difference to be significant.

A final study, including 65 patients, measured a mean LV Tei index of 0.33 in healthy controls, 0.34 in patients who received < 200 mg/m^2^, and 0.45 in patients who received ≥ 200 mg/m^2^ of anthracyclines. Both the difference between the lowest dose group and the highest dose group and the difference between the highest dose group and the control group were statistically significant.

### Biomarker levels – continuous results

See Table 4 for a detailed overview of biomarker levels before and after cardiotoxic childhood cancer treatment.

**Table 4:** Cardiac biomarker levels before and after therapy in children with cancer treated with anthracyclines, mitoxantrone and/or radiotherapy involving the heart

Study	Number of participants	Biomarker level before therapy^a^	Timing of measurement after therapy^b^	Biomarker level after therapy^a^	*P* value
*Troponin*					
Troponin I (ng/mL)					
Al-Biltagi 2012 [28]	25	0.055 ± 0.003	Acute	0.061 ± 0.005	0.002
El Amrousy 2022 [62]	30	Mean < 0.01	Acute/early	0.050 ± 0.012	< 0.05
El-Shitany 2012 [37]	25	nm	Acute/early	0.061 ± 0.005	nm
Troponin I (pg/mL)					
Hagag 2019 [42]	40	37.3 ± 8.71	Acute/early	75.5 ± 7.71	0.027
Radu 2019 [53]	48	Median 0.1, IQR 0.1–0.1	Acute	Median 0.1, IQR 0.1–0.1	nm
			Early	Median 0.1, IQR 0.1–0.2	< 0.001
Cardiac troponin I (ng/mL)					
Erkus 2007 [38]	29	0.020 ± 0.006	Early	0.024 ± 0.009	nm
Gupta 2018 [41]	40	0.01 ± 0.00	Early	0.011 ± 0.003	nm
Oztarhan 2011 [52]	276	0.02 ± 0.22	Acute/early	Cumulative ANT dose 30-90 mg/m^2^: 0.02 ± 0.01	nm
				Cumulative ANT dose 120-180 mg/m^2^: 0.02 ± 0.02	nm
				Cumulative ANT dose ≥ 210 mg/m^2^: 0.04 ± 0.14	nm
Cardiac troponin C (ng/mL)					
Hu 2018 (2) [44]	131	nm	Acute	Cumulative PIR dose < 100 mg/m^2^: mean < 0.05	nm
				Cumulative PIR dose 100-200 mg/m^2^: mean < 0.05	nm
				Cumulative PIR dose ≥ 200 mg/m^2^: mean < 0.05	nm
Mavinkurve-Groothuis 2013 [49]	60	Median 0.01	Early	0.01, 0.01–0.02	nm
High-sensitive cardiac troponin T (ng/L)					
Cheung 2020	39	3.50 ± 5.44	Early	11.53 ± 8.52	< 0.001
*Brain natriuretic peptide (pg/mL)*					
Erkus 2007 [38]	29	4.09 ± 2.26	Early	7.47 ± 3.16	< 0.05
N-terminal prohormone of brain natriuretic peptide (pg/mL)					
El Amrousy 2022 [62]	30	37.1 ± 7.7	Acute/early	88.8 ± 13.6	< 0.05
Gupta 2018 [41]	40	5.00 ± 0.00	Early	98.60 ± 54.24	nm
Hu 2018 (2) [44]	131	nm	Acute	Cumulative PIR dose < 100 mg/m^2^: 142.93 ± 104.43	-
				Cumulative PIR dose 100-200 mg/m^2^: 158.27 ± 78.18	0.978 ‡
				Cumulative PIR dose ≥ 200 mg/m^2^: 1725.90 ± 5634.78	0.013 ‡
N-terminal prohormone of brain natriuretic peptide (pmol/L)					
Mavinkurve-Groothuis 2013 [49]	60	13, 2–185	Early	11, 1–68	nm
*Creatine kinase (U/L)*					
Al-Biltagi 2012 [28]	25	50.60 ± 8.55	Acute	48.61 ± 6.56	0.62
El-Shitany 2012 [37]	25	50.6 ± 25.67	Acute/early	47.6 ± 20.45	nm
Creatine kinase, myocardial band (U/L)					
El Amrousy 2022 [62]	30	11.6 ± 3.6	Acute/early	46 ± 7.2	< 0.05
Hu 2018 (2) [44]	131	nm	Acute	Cumulative PIR dose < 100 mg/m^2^: 30.06 ± 11.43	-
				Cumulative PIR dose 100-200 mg/m^2^: 27.53 ± 8.80	nm
				Cumulative PIR dose ≥ 200 mg/m^2^: 25.67 ± 12.31	nm
Creatine kinase, myocardial band (ng/mL)					
Gupta 2018 [41]	40	1.00 ± 0.00	Early	1.21 ± 0.44	nm

*Abbreviations*: *ANT* Anthracycline, *IQR* Interquartile range, *PIR* Pirarubicin

^a^unless otherwise specified, mean is provided with standard deviation (x ± y) and median with range (x, y–z)

^b^acute (within one week after a treatment); early (within one year after start of treatment); acute/early (acute and early not separable)

^‡^ *P* values in comparison to the lowest dose group are presented

### Troponin

Tn levels were measured in 11 studies. In five of these studies, troponin I (TnI) was measured. Three publications, including 95 patients (25 to 40 patients per study), presented mean TnI levels at baseline (ranging from < 0.01 ng/mL to 0.055 ng/mL) and post-treatment (ranging from 0.050 ng/mL to 0.0755 ng/mL). All three studies found higher post-treatment than baseline TnI levels, and all these changes were significant. One publication including 25 patients only reported a mean post-treatment TnI value of 0.061 ng/mL. Another study including 48 patients reported median pre- and post-treatment levels; both were 0.1 pg/mL, but a statistically significant difference was established nonetheless.

In three studies, cardiac troponin I (cTnI) was measured. Two studies, including 69 patients (29 and 40 patients, respectively), measured mean pre-treatment values of 0.020 and 0.01 ng/mL; mean post-treatment values were 0.024 and 0.011 ng/mL, respectively. Another study including 276 patients performed cTnI measurements at baseline and in subgroups of patients who received 30 to 90, 120 to 180 and ≥ 210 mg/m^2^ of anthracyclines; cTnI levels after treatment were 0.02, 0.02, 0.02 and 0.04 ng/mL, respectively. Significance of these results was reported for none of the three studies.

Cardiac troponin C (cTnT) was measured in two studies. In one study including 60 patients, median values of 0.01 ng/mL at baseline as well as after treatment were measured. In the other study, including 131 patients, baseline measurements were not performed, but levels were < 0.05 ng/mL in all cumulative pirarubicin dose categories (< 100, 100 to 200, and ≥ 200 mg/m^2^). Neither of these publications reported on significance.

A final study including 39 patients measured high-sensitive cardiac troponin T (hs-cTnT): mean pre-treatment level was 0.0035 ng/mL and mean post-treatment level was 0.01153 ng/mL.

### Brain natriuretic peptide

Measurements of forms of BNP were done in five studies. One publication including 29 patients measured a mean pre-treatment BNP value of 4.09 pg/mL and a significantly increased mean post-treatment value of 7.47 pg/mL.

The remaining four studies measured NT-proBNP levels. One study, including 30 patients, reported a mean pre-treatment values of 37.1 pg/mL and a significantly higher post-treatment value of 88.8 pg/mL; these values were 5.00 and 98.60 pg/mL, respectively, in another study including 40 patients (significance not reported). A study including 60 patients reported median pre- and post-treatment levels of 13 and 11 pmol/L (significance not reported). A final study, including 131 patients, reported post-treatment measurements in different cumulative pirarubicin dose categories and found the following mean values: < 100 mg/m^2^, 142.93 pg/mL; 100 to 200 mg/m^2^, 158.27 pg/mL; ≥ 200 mg/m^2^, 1725.90 pg/mL. The difference between the highest dose group and the lowest dose group was significant, the difference between the middle dose group and the lowest dose group was not.

### Creatine kinase

CK was measured in five studies. In two publications describing mean pre- and post-treatment CK levels, presumably in the same cohort of 25 patients, slightly different results were reported (50.6U/L and 50.6U/L pre-treatment and 48.61U/L and 47.6U/L post-treatment, respectively. The first publication reported the difference not to be significant, the second publication did not report on significance.

Two studies, including 70 patients, measured pre- and post-treatment values of the myocardial band of creatine kinase (CK-MB); mean pre- and post-treatment values were 11.6U/L and 46U/L (significantly higher) and 1.00 ng/mL and 1.21 ng/mL (significance not reported), respectively. Finally, a study including 131 patients found levels of 30.06, 27.53 and 25.67U/L in patients who received < 100, 100 to 200, and ≥ 200 mg/m^2^ of anthracyclines, respectively (significance not reported).

### Risk factors

Multivariate risk factor analyses for acute and early-onset cardiotoxicity were performed in three studies including 1202 patients. Getz et al. [40] found that, regarding age at diagnosis, the risk of cardiotoxicity was significantly lower in children below two years of age when compared with children aged between two and ten years (hazard ratio 0.21, 95% CI 0.06–0.69, *P* < 0.05). Black children had a significantly higher risk of cardiotoxicity compared with white children (hazard ratio 2.18, 95% CI 1.27–3.75, *P* < 0.05). The other factors that were assessed (i.e. sex, ethnicity, weight category, cytogenetic risk group, initial white blood count, randomized treatment arm, microbiologically documented bloodstream infection during treatment, age ≥ 11 years category and other race) did not prove to influence the risk of cardiotoxicity (for more detailed information see Additional file 3).

Samosir et al. [66] found that children older than four years had a significantly higher risk of cardiotoxicity compared with children younger than four years (prevalence ratio 1.128, 95% CI 1.015–1.254, *P* < 0.001). Children treated in the high risk group (containing more anthracyclines) had a significantly higher risk than children treated in the standard risk group (prevalence ratio 1.135, 95% CI 1.016–1.269, *P* < 0.001). Finally, a cumulative daunorubicin dose > 120 mg/m^2^ was associated with a higher risk than a cumulative dose ≤ 120 mg/m^2^ (prevalence ratio 1.161, 95% CI 1.019–1.324, *P* = 0.001).

Hu et al. [43] performed a multiple linear regression to predict post-treatment NT-proBNP, age and corrected QT interval (QTc) based on cumulative anthracycline dose. They found *F* = 11.359 (*P* < 0.001); the standard coefficient of NT-proBNP was 0.423 (*P* = 0), that of age was 0.184 (*P* = 0.021) and that of QTc was 0.191 (*P* = 0.018). The cumulative dose of anthracyclines had the most significant impact on the post-treatment serum NT-proBNP.

## Discussion

Acute and early-onset cardiotoxicity was studied in 45 studies including 7797 children with cancer. The characteristics of the studied cohorts (for example treatment and age at diagnosis), as well as the diagnostic modalities and the outcome definitions used in the different studies, proved to be highly heterogeneous, which might explain part of the large differences in cardiotoxicity prevalence. Based on the cardiotoxicity definitions used, highly variable prevalence rates were established: 0.0–56.4% for systolic dysfunction, 30.0–100% for diastolic dysfunction, 0.0–38.1% for combinations of echocardiography and/or clinical parameters, 0.0–25.5% for clinical symptoms and 0.0–37.5% for biomarker levels. Most studies did not separate acute from early-onset cardiotoxicity. FS and EF significantly decreased during treatment; GLS decreased during treatment, but not all studies found the changes to be significant. Tei ratio significantly increased during treatment, changes in E/A ratio were inconsistent. TnI increased significantly during treatment; changes in TnT, BNP and CK were inconsistent. Cumulative anthracycline dose was found to be a risk factor for acute and early-onset cardiotoxicity. In most of the studies, the presence of bias (especially selection bias, detection bias (for non-biomarker outcomes) and confounding, but in many studies also attrition bias) could not be ruled out, often due to lack of reporting. As a result there is a risk of either over- or underestimation of the identified results, but as the studies in general did not report the reasons for the presence of the different types of bias unfortunately we cannot be more specific.

### Echocardiography

In pediatric cardio-oncology, traditionally, FS and EF have been used to express systolic cardiac function. Among the studies included in this review, a wide range of prevalence of cardiotoxicity based on FS and EF was found (0.0 to 40.0%). This may partly be due to the variation in cut-off values, and the fact that some studies defined cardiotoxicity based on absolute values, while other studies reported on changes compared to baseline values. A pitfall of defining cardiotoxicity based on these parameters may be that changes only occur after a significant degree of cardiac damage and remodeling. In addition, interpretation of FS and EF during treatment can be complicated by the influence of treatment-related factors such as infections and differences in loading conditions such as hyperhydration. These results seem comparable to late-onset cardiotoxicity: in a recent systematic review on asymptomatic systolic dysfunction in anthracycline-treated childhood cancer survivors [73], the prevalence of abnormal EF was 1–6% and the prevalence of abnormal FS was 0.3–30%.

In the adult setting, decrease in GLS has been proven to be a sensitive predictor of cardiac injury [74, 75]. The one study included in this review defining cardiotoxicity using GLS found a prevalence of 56.4%, which may suggest a higher sensitivity than the conventional echocardiographic parameters. As only 39 patients were included in this study, data are insufficient to draw solid conclusions.

Diastolic dysfunction has been found to be present in 11% of childhood cancer survivors and in 8.7% of childhood cancer survivors with normal EF, suggesting it might be more sensitive in detecting cardiotoxicity [76]. In only two studies in our review, cardiotoxicity was defined based on diastolic parameters. As one study used E/A ratio and the other study used Tei index, the two are not comparable. A high prevalence of abnormal diastolic function (30.0 to 100%), however, stands out. Unfortunately, it was not possible to compare systolic and diastolic dysfunction.

The studies in our review consistently show a significant decrease of FS, EF and GLS post-treatment when compared with baseline. In some studies, the decrease is significantly greater in subgroups that received higher cumulative doses of anthracyclines, suggesting (but not proving) a dose–effect relationship. With regard to diastolic function, it is more difficult to draw conclusions. Changes in E/A ratio are inconsistent. The studies in our review do report a significant increase in Tei index post-treatment compared to baseline, but the number of patients in which this was measured is limited.

### Combination of echocardiographic and/or clinical parameters

The studies in this review using the NCI CTCAE found wide ranges of prevalence for the different grades of cardiac dysfunction (0.0 to 35.2%); as would be expected, higher grades of dysfunction were less common than lower grades. Intuitively, a standardized approach to grade adverse events (such as the NCI CTCAE) leads to a high reproducibility of reported prevalence. In practice, however, inter-observer variability proves to be considerable [77, 78], this might have played a role in the variance in prevalence.

The studies in which combinations of echocardiographic and/or clinical parameters other than the NCI CTCAE were used had widely differing definitions of cardiotoxicity; cardiotoxicity in these studies varied between 0.0 and 38.1%.

### Clinical symptoms

Interpretation of the prevalence numbers found in the studies defining cardiotoxicity based on clinical symptoms was hampered by the great heterogeneity of definitions used, and the fact that the clinical outcomes were often not clearly defined. Looking at the two studies that were deemed to have well-defined clinical outcomes, prevalence ranged from 1.6 to 1.9%. Defining cardiotoxicity using only clinical symptoms implicitly means measuring clinically relevant myocardial damage. The striking difference between prevalence based on echocardiography and prevalence based on clinical symptoms probably reflects the evolution from subclinical myocardial damage to clinically relevant impaired cardiac function, even in the acute and early-onset setting. Taking into account that the cumulative incidence of heart failure increases to 4.8–10.6% in long-term childhood cancer survivors [79], this probably represents an ongoing process.

### Biomarker levels

A recent review has shown that biomarkers are of limited value in detecting LV dysfunction in childhood cancer survivors [80], In the studies in this review, abnormal levels of biomarkers were more common for BNP (19.5 to 37.5%) than for troponin T (TnT, 0.0 to 8.8%) and TnI (0.0 to 6.9%). With regard to absolute biomarker levels, TnI showed a consistent significant increase post-treatment when compared to baseline. Results were less consistent for TnT, BNP and CK, with some studies showing increased levels and some studies showing equal levels after treatment. Correlation to echocardiographic parameters and clinical outcomes will be required to determine the clinical relevance of these findings.

### Risk factors

Multivariate analyses, which were conducted in only three studies in this review, allow us to compare risk factors for acute and early-onset cardiotoxicity with known risk factors for late-onset cardiotoxicity, such as higher cumulative anthracycline dose, younger age and female sex [11, 81, 82]. Consistent with late-onset cardiotoxicity, higher cumulative anthracycline dose was found to be a risk factor for acute and early-onset cardiotoxicity. Interestingly, older age (defined as between two and ten years in one study, and as four years or older in another study) was identified as a risk factor for acute and early-onset cardiotoxicity when compared with younger age (defined as below two years or below four years, respectively). Sex did not prove to influence the risk of acute and early-onset cardiotoxicity. Furthermore, black race was identified as a risk factor for acute and early-onset cardiotoxicity.

## Conclusion

Various definitions, based on (combinations of) echocardiography, clinical symptoms and biomarker levels, have been used to describe acute and early-onset cardiotoxicity due to childhood cancer treatment, complicating the establishment of its exact prevalence. Damage to cardiomyocytes probably predisposes for and predates clinical symptoms. While FS and EF significantly decrease during treatment, GLS may be a more sensitive marker of cardiotoxicity, although data in children are still too limited to confirm this assumption based on adult data. Cumulative anthracycline dose proves to be an important risk factor for acute and early-onset cardiotoxicity, similar to late-onset cardiotoxicity. Uniform international guidelines to prospectively monitor acute and early-onset cardiotoxicity in children will provide more reliable information on prevalence, risk factors and possible preventive measures.

### Supplementary Information

**Additional file 1. **Search strategy.

**Additional file 2. **Risk of bias assessment criteria for observational studies.

**Additional file 3. **Data extraction forms.

**Additional file 4. **Overview of oncologic diagnoses.

**Additional file 5. **Risk of bias in included studies.

## Data Availability

All data extraction forms generated during this study are included in this published articles supplementary information files.

## Abbreviations

BNP: Brain natriuretic peptide
CCS: Childhood cancer survivors
CI: Confidence interval
CK: Creatine kinase
CK-MB: Myocardial band of creatine kinase
cTnI: Cardiac troponin I
cTnT: Cardiac troponin T
EF: Ejection fraction
FS: Fractional shortening
GLS: Global longitudinal strain
hs-cTnT: High-sensitive cardiac troponin T
LV: Left ventricular
NCI CTCAE: National Cancer Institute Common Terminology Criteria for Adverse Events
NT-proBNP: N-terminal prohormone of brain natriuretic peptide
proBNP: Prohormone of brain natriuretic peptide
QTc: Corrected QT interval
Tn: Troponin
TnI: Troponin I
TnT: Troponin T

## References

[1] Siegel RL, Miller KD, Jemal A. Cancer statistics, 2019. CA Cancer J Clin 2019;69(1):7–34 10.3322/caac.21551. [published Online First: Epub Date]. 10.3322/caac.21551 30620402

[2] Feijen E, Font-Gonzalez A, Van der Pal HJH, et al. Risk and Temporal Changes of Heart Failure Among 5-Year Childhood Cancer Survivors: a DCOG-LATER Study. J Am Heart Assoc 2019;8(1):e009122. 10.1161/JAHA.118.009122. [published Online First: Epub Date]. 10.1161/JAHA.118.009122 PMC6405698 30595059

[3] Fidler MM, Reulen RC, Henson K, et al. Population-Based Long-Term Cardiac-Specific Mortality Among 34 489 Five-Year Survivors of Childhood Cancer in Great Britain. Circulation 2017;135(10):951–63. 10.1161/CIRCULATIONAHA.116.024811. [published Online First: Epub Date]. 10.1161/CIRCULATIONAHA.116.024811 PMC5338891 28082386

[4] Chow EJ, Leger KJ, Bhatt NS, et al. Paediatric cardio-oncology: epidemiology, screening, prevention, and treatment. Cardiovasc Res 2019;115(5):922–34. 10.1093/cvr/cvz031 . [published Online First: Epub Date]. 10.1093/cvr/cvz031 PMC6452306 30768157

[5] Armenian SH, Armstrong GT, Aune G, et al. Cardiovascular Disease in Survivors of Childhood Cancer: Insights Into Epidemiology, Pathophysiology, and Prevention. J Clin Oncol 2018;36(21):2135–44. 10.1200/JCO.2017.76.3920. [published Online First: Epub Date]. 10.1200/JCO.2017.76.3920 PMC6804893 29874141

[6] Bansal N, Amdani S, Lipshultz ER, Lipshultz SE. Chemotherapy-induced cardiotoxicity in children. Expert Opin Drug Metab Toxicol 2017;13(8):817–32. 10.1080/17425255.2017.1351547. [published Online First: Epub Date]. 10.1080/17425255.2017.1351547 28679288

[7] Moller TR, Garwicz S, Barlow L, et al. Decreasing late mortality among five-year survivors of cancer in childhood and adolescence: a population-based study in the Nordic countries. J Clin Oncol 2001;19(13):3173–81. 10.1200/JCO.2001.19.13.3173. [published Online First: Epub Date]. 10.1200/JCO.2001.19.13.3173 11432883

[8] Mertens AC, Yasui Y, Neglia JP, et al. Late mortality experience in five-year survivors of childhood and adolescent cancer: the Childhood Cancer Survivor Study. J Clin Oncol 2001;19(13):3163–72. 10.1200/JCO.2001.19.13.3163. [published Online First: Epub Date]. 10.1200/JCO.2001.19.13.3163 11432882

[9] Grenier MA, Lipshultz SE (1998). Epidemiology of anthracycline cardiotoxicity in children and adults. Semin Oncol.

[10] Lipshultz SE, Alvarez JA, Scully RE. Anthracycline associated cardiotoxicity in survivors of childhood cancer. Heart 2008;94(4):525–33. 10.1136/hrt.2007.136093. [published Online First: Epub Date]|. 10.1136/hrt.2007.136093 18347383

[11] Lipshultz SE, Colan SD, Gelber RD, Perez-Atayde AR, Sallan SE, Sanders SP. Late cardiac effects of doxorubicin therapy for acute lymphoblastic leukemia in childhood. N Engl J Med 1991;324(12):808–15. 10.1056/NEJM199103213241205. [published Online First: Epub Date]. 10.1056/NEJM199103213241205 1997853

[12] Barry E, Alvarez JA, Scully RE, Miller TL, Lipshultz SE. Anthracycline-induced cardiotoxicity: course, pathophysiology, prevention and management. Expert Opin Pharmacother 2007;8(8):1039–58. 10.1517/14656566.8.8.1039. [published Online First: Epub Date]|. 10.1517/14656566.8.8.1039 17516870

[13] Kremer LC, van der Pal HJ, Offringa M, van Dalen EC, Voute PA. Frequency and risk factors of subclinical cardiotoxicity after anthracycline therapy in children: a systematic review. Ann Oncol 2002;13(6):819–29. 10.1093/annonc/mdf167. [published Online First: Epub Date]|. 10.1093/annonc/mdf167 12123328

[14] Kremer LC, van Dalen EC, Offringa M, Voute PA. Frequency and risk factors of anthracycline-induced clinical heart failure in children: a systematic review. Ann Oncol 2002;13(4):503–12. 10.1093/annonc/mdf118. [published Online First: Epub Date]. 10.1093/annonc/mdf118 12056699

[15] Shankar SM, Marina N, Hudson MM, et al. Monitoring for cardiovascular disease in survivors of childhood cancer: report from the Cardiovascular Disease Task Force of the Children's Oncology Group. Pediatrics 2008;121(2):e387–96. 10.1542/peds.2007-0575. [published Online First: Epub Date]. 10.1542/peds.2007-0575 18187811

[16] Landier W, Bhatia S, Eshelman DA, et al. Development of risk-based guidelines for pediatric cancer survivors: the Children's Oncology Group Long-Term Follow-Up Guidelines from the Children's Oncology Group Late Effects Committee and Nursing Discipline. J Clin Oncol 2004;22(24):4979–90. 10.1200/JCO.2004.11.032. [published Online First: Epub Date]. 10.1200/JCO.2004.11.032 15576413

[17] Dutch Childhood Oncology Group. Guidelines for follow-up in survivors of childhood cancer 5 years after diagnosis. English translation. 2014. Available at: https://www.skion.nl/workspace/uploads/vertaling-richtlijn-LATER-versie-final-okt-2014_2.pdf.

[18] Armenian SH, Hudson MM, Mulder RL, et al. Recommendations for cardiomyopathy surveillance for survivors of childhood cancer: a report from the International Late Effects of Childhood Cancer Guideline Harmonization Group. Lancet Oncol 2015;16(3):e123–36. 10.1016/S1470-2045(14)70409-7. [published Online First: Epub Date]. 10.1016/S1470-2045(14)70409-7 PMC4485458 25752563

[19] Ehrhardt MJ, Leerink JM, Mulder RL, et al. Systematic review and updated recommendations for cardiomyopathy surveillance for survivors of childhood, adolescent, and young adult cancer from the International Late Effects of Childhood Cancer Guideline Harmonization Group. The Lancet Oncology 2023;24(3):e108-e20. 10.1016/s1470-2045(23)00012-8. [published Online First: Epub Date]|. 10.1016/S1470-2045(23)00012-8 37052966

[20] Armenian SH, Gelehrter SK, Chow EJ. Strategies to prevent anthracycline-related congestive heart failure in survivors of childhood cancer. Cardiol Res Pract 2012;2012:713294. 10.1155/2012/713294. [published Online First: Epub Date]|. 10.1155/2012/713294 PMC3426199 22928146

[21] Lipshultz SE. Dexrazoxane for protection against cardiotoxic effects of anthracyclines in children. J Clin Oncol 1996;14(2):328–31. 10.1200/JCO.1996.14.2.328. [published Online First: Epub Date]. 10.1200/JCO.1996.14.2.328 8636739

[22] Steinherz LJ, Graham T, Hurwitz R (1992). Guidelines for cardiac monitoring of children during and after anthracycline therapy: report of the Cardiology Committee of the Childrens Cancer Study Group. Pediatrics.

[23] van Dalen EC, van den Brug M, Caron HN, Kremer LC. Anthracycline-induced cardiotoxicity: comparison of recommendations for monitoring cardiac function during therapy in paediatric oncology trials. Eur J Cancer 2006;42(18):3199–205. 10.1016/j.ejca.2006.08.002. [published Online First: Epub Date]|. 10.1016/j.ejca.2006.08.002 17011186

[24] Grimes DA, Schulz KF. Cohort studies: marching towards outcomes. Lancet 2002;359(9303):341–5. 10.1016/S0140-6736(02)07500-1. [published Online First: Epub Date]. 10.1016/S0140-6736(02)07500-1 11830217

[25] Laupacis A, Wells G, Richardson WS, Tugwell P. Users' guides to the medical literature. V. How to use an article about prognosis. Evidence-Based Medicine Working Group. JAMA 1994;272(3):234–7. 10.1001/jama.272.3.234. [published Online First: Epub Date]. 10.1001/jama.272.3.234 8022043

[26] Brown LD, Cai TT, DasGupta  A (2001). Interval Estimation for a Binomial Proportion. Statistical Science.

[27] Agha H, Shalaby L, Attia W, Abdelmohsen G, Aziz OA, Rahman MY. Early Ventricular Dysfunction After Anthracycline Chemotherapy in Children. Pediatr Cardiol 2016;37(3):537–44. 10.1007/s00246-015-1311-5. [published Online First: Epub Date]. 10.1007/s00246-015-1311-5 26667956

[28] Al-Biltagi M, Abd Rab Elrasoul Tolba O, El-Shanshory MR, Abd El-Aziz El-Shitany N, El-Sayed El-Hawary E. Strain echocardiography in early detection of Doxorubicin-induced left ventricular dysfunction in children with acute lymphoblastic leukemia. ISRN Pediatr 2012;2012:870549. 10.5402/2012/870549. [published Online First: Epub Date]. 10.5402/2012/870549 PMC3302013 22518327

[29] Asselin BL, Devidas M, Chen L, et al. Cardioprotection and Safety of Dexrazoxane in Patients Treated for Newly Diagnosed T-Cell Acute Lymphoblastic Leukemia or Advanced-Stage Lymphoblastic Non-Hodgkin Lymphoma: A Report of the Children's Oncology Group Randomized Trial Pediatric Oncology Group 9404. J Clin Oncol 2016;34(8):854–62. 10.1200/JCO.2015.60.8851. [published Online First: Epub Date]. 10.1200/JCO.2015.60.8851 PMC4872007 26700126

[30] Berrak SG, Ewer MS, Jaffe N, et al. Doxorubicin cardiotoxicity in children: reduced incidence of cardiac dysfunction associated with continuous-infusion schedules. Oncol Rep 2001;8(3):611–4. 10.3892/or.8.3.611. [published Online First: Epub Date]. 10.3892/or.8.3.611 11295089

[31] Brown TR, Vijarnsorn C, Potts J, Milner R, Sandor GG, Fryer C. Anthracycline induced cardiac toxicity in pediatric Ewing sarcoma: a longitudinal study. Pediatr Blood Cancer 2013;60(5):842–8. 10.1002/pbc.24404. [published Online First: Epub Date]. 10.1002/pbc.24404 23382019

[32] Chen C, Heusch A, Donner B, Janssen G, Gobel U, Schmidt KG. Present risk of anthracycline or radiation-induced cardiac sequelae following therapy of malignancies in children and adolescents. Klin Padiatr 2009;221(3):162–6. 10.1055/s-0029-120722. [published Online First: Epub Date]. 10.1055/s-0029-120722 19437364

[33] Cheung YF, Li VW, Lai CT, et al. Circulating high-sensitivity troponin T and microRNAs as markers of myocardial damage during childhood leukaemia treatment. Pediatr Res 2021;89(5):1245–52. 10.1038/s41390-020-1049-5. [published Online First: Epub Date]. 10.1038/s41390-020-1049-5 32634817

[34] Choi HS, Park ES, Kang HJ, et al. Dexrazoxane for preventing anthracycline cardiotoxicity in children with solid tumors. J Korean Med Sci 2010;25(9):1336–42. 10.3346/jkms.2010.25.9.1336. [published Online First: Epub Date]. 10.3346/jkms.2010.25.9.1336 PMC2923785 20808678

[35] Creutzig U, Diekamp S, Zimmermann M, Reinhardt D. Longitudinal evaluation of early and late anthracycline cardiotoxicity in children with AML. Pediatr Blood Cancer 2007;48(7):651–62. 10.1002/pbc.21105. [published Online First: Epub Date]. 10.1002/pbc.21105 17183582

[36] de Matos Neto RP, Petrilli AS, Silva CM, et al. Left ventricular systolic function assessed by echocardiography in children and adolescents with osteosarcoma treated with doxorubicin alone or in combination with dexrazoxane. Arq Bras Cardiol 2006;87(6):763–71. 10.1590/s0066-782x2006001900013. [published Online First: Epub Date]. 10.1590/s0066-782x2006001900013 17262115

[37] El-Shitany NA, Tolba OA, El-Shanshory MR, El-Hawary EE. Protective effect of carvedilol on adriamycin-induced left ventricular dysfunction in children with acute lymphoblastic leukemia. J Card Fail 2012;18(8):607–13. 10.1016/j.cardfail.2012.06.416. [published Online First: Epub Date]. 10.1016/j.cardfail.2012.06.416 22858075

[38] Erkus B, Demirtas S, Yarpuzlu AA, Can M, Genc Y, Karaca L. Early prediction of anthracycline induced cardiotoxicity. Acta Paediatr 2007;96(4):506–9. 10.1111/j.1651-2227.2006.00174.x. [published Online First: Epub Date]. 10.1111/j.1651-2227.2006.00174.x 17391467

[39] Fukumi D, Uchikoba Y, Maeda M, Ogawa S. Longitudinal evaluation of anthracycline cardiotoxicity by signal-averaged electrocardiography in children with cancer. Pediatr Int 2002;44(2):134–40. 10.1046/j.1328-8067.2001.01526.x. [published Online First: Epub Date]. 10.1046/j.1328-8067.2001.01526.x 11896869

[40] Getz KD, Sung L, Ky B, et al. Occurrence of Treatment-Related Cardiotoxicity and Its Impact on Outcomes Among Children Treated in the AAML0531 Clinical Trial: A Report From the Children's Oncology Group. J Clin Oncol 2019;37(1):12–21. 10.1200/JCO.18.00313. [published Online First: Epub Date]. 10.1200/JCO.18.00313 PMC6354770 30379624

[41] Gupta V, Kumar Singh S, Agrawal V, Bali Singh T. Role of ACE inhibitors in anthracycline-induced cardiotoxicity: A randomized, double-blind, placebo-controlled trial. Pediatr Blood Cancer 2018;65(11):e27308 doi: 10.1002/pbc.27308. [published Online First: Epub Date]. 10.1002/pbc.27308 30009543

[42] Hagag AA, El Shehaby WA, El-Abasy AI, Mabrouk MM. Protective Role of Silymarin in Early Doxorubicin-induced Cardiac Dysfunction in Children with Acute Lymphoblastic Leukemia. Infect Disord Drug Targets 2019;19(2):133–40 doi: 10.2174/1871526518666180803141827. [published Online First: Epub Date]. 10.2174/1871526518666180803141827 30073931

[43] Hu H, Zhang W, Huang D, Yang Q, Li J, Gao Y. Cardiotoxicity of anthracycline (ANT) treatment in children with malignant tumors. Pediatr Hematol Oncol 2018;35(2):111–20 doi: 10.1080/08880018.2018.1459983. [published Online First: Epub Date]. 10.1080/08880018.2018.1459983 29648903

[44] Hu HM, Zhang XL, Zhang WL, Huang DS, Du ZD. Detection of Subclinical Anthracyclines' Cardiotoxicity in Children with Solid Tumor. Chin Med J (Engl) 2018;131(12):1450–56. 10.4103/0366-6999.233950. [published Online First: Epub Date]|. 10.4103/0366-6999.233950 PMC6006810 29893362

[45] Ishii M, Tsutsumi T, Himeno W, et al. Sequential evaluation of left ventricular myocardial performance in children after anthracycline therapy. Am J Cardiol 2000;86(11):1279–81, A9. 10.1016/s0002-9149(00)01222-4. [published Online First: Epub Date]. 10.1016/s0002-9149(00)01222-4 11090811

[46] Kang M, Kim KI, Song YC, Shin WG, Oh JM. Cardioprotective effect of early dexrazoxane use in anthracycline treated pediatric patients. J Chemother 2012;24(5):292–6. 10.1179/1973947812Y.0000000038. [published Online First: Epub Date]|. 10.1179/1973947812Y.0000000038 23182049

[47] Khairat I, Khalfallah M, Shaban A, Farag IA, Elkady A. Right ventricular 2D speckle-tracking echocardiography in children with osteosarcoma under chemotherapy. Egypt Heart J 2019;71(1):23. 10.1186/s43044-019-0028-9. [published Online First: Epub Date]. 10.1186/s43044-019-0028-9 PMC6868075 31748972

[48] Krischke M, Hempel G, Voller S, et al. Pharmacokinetic and pharmacodynamic study of doxorubicin in children with cancer: results of a "European Pediatric Oncology Off-patents Medicines Consortium" trial. Cancer Chemother Pharmacol 2016;78(6):1175–84. 10.1007/s00280-016-3174-8. [published Online First: Epub Date]. 10.1007/s00280-016-3174-8 PMC5114325 27770238

[49] Mavinkurve-Groothuis AM, Marcus KA, Pourier M, et al. Myocardial 2D strain echocardiography and cardiac biomarkers in children during and shortly after anthracycline therapy for acute lymphoblastic leukaemia (ALL): a prospective study. Eur Heart J Cardiovasc Imaging 2013;14(6):562–9. 10.1093/ehjci/jes217. [published Online First: Epub Date]|. 10.1093/ehjci/jes217 23109647

[50] Moke DJ, Schubert LE, Sun HY, Printz BF, Dietz AC. Utility of Echocardiography as Screening for Late-onset Anthracycline-induced Cardiotoxicity in Pediatric Cancer Survivors: Observations from the First Decade After End of Therapy. J Pediatr Hematol Oncol 2018;40(5):e283-e88. 10.1097/MPH.0000000000001087. [published Online First: Epub Date]|. 10.1097/MPH.0000000000001087 29432303

[51] Moussa E, Zamzam M, Kamel A, et al. Risk stratification and pattern of cardiotoxicity in pediatric Ewing sarcoma. J Egypt Natl Canc Inst 2017;29(1):53–56. 10.1016/j.jnci.2016.12.001. [published Online First: Epub Date]|. 10.1016/j.jnci.2016.12.001 28258912

[52] Oztarhan K, Guler S, Aktas B, Arslan M, Salcioglu Z, Aydogan G. The value of echocardiography versus cardiac troponin I levels in the early detection of anthracycline cardiotoxicity in childhood acute leukemia: prospective evaluation of a 7-year-long clinical follow-up. Pediatr Hematol Oncol 2011;28(5):380–94. 10.3109/08880018.2011.563772. [published Online First: Epub Date]. 10.3109/08880018.2011.563772 21699467

[53] Radu LE, Ghiorghiu I, Oprescu A, Dorobantu D, Arion C, Colita A. Cardiotoxicity evaluation in pediatric patients with acute lymphoblastic leukemia - results of prospective study. Med Ultrason 2019;21(4):449–55. 10.11152/mu-2012. [published Online First: Epub Date]. 10.11152/mu-2012 31765454

[54] Sagi JC, Egyed B, Kelemen A, et al. Possible roles of genetic variations in chemotherapy related cardiotoxicity in pediatric acute lymphoblastic leukemia and osteosarcoma. BMC Cancer 2018;18(1):704. 10.1186/s12885-018-4629-6. [published Online First: Epub Date]|. 10.1186/s12885-018-4629-6 PMC6029426 29970035

[55] Schramm F, Zimmermann M, Jorch N, et al. Daunorubicin during delayed intensification decreases the incidence of infectious complications - a randomized comparison in trial CoALL 08–09. Leuk Lymphoma 2019;60(1):60–68. 10.1080/10428194.2018.1473575. [published Online First: Epub Date]|. 10.1080/10428194.2018.1473575 29966458

[56] Shaikh AS, Saleem AF, Mohsin SS, Alam MM, Ahmed MA. Anthracycline-induced cardiotoxicity: prospective cohort study from Pakistan. BMJ Open 2013;3(11):e003663. 10.1136/bmjopen-2013-003663. [published Online First: Epub Date]. 10.1136/bmjopen-2013-003663 PMC3840341 24259388

[57] Stohr W, Paulides M, Brecht I, et al. Comparison of epirubicin and doxorubicin cardiotoxicity in children and adolescents treated within the German Cooperative Soft Tissue Sarcoma Study (CWS). J Cancer Res Clin Oncol 2006;132(1):35–40. 10.1007/s00432-005-0041-0. [published Online First: Epub Date]. 10.1007/s00432-005-0041-0 PMC12161019 16205946

[58] Tantawy AA, Elmasry OA, Shaaban M, Toaima DN, El Shahat AM. Radionuclide ventriculography detects early anthracycline cardiotoxicity in children with Hodgkin lymphoma. J Pediatr Hematol Oncol 2011;33(4):e132–7. 10.1097/MPH.0b013e318212eb6b. [published Online First: Epub Date]. 10.1097/MPH.0b013e318212eb6b 21516011

[59] Temming P, Qureshi A, Hardt J, et al. Prevalence and predictors of anthracycline cardiotoxicity in children treated for acute myeloid leukaemia: retrospective cohort study in a single centre in the United Kingdom. Pediatr Blood Cancer 2011;56(4):625–30. 10.1002/pbc.22908. [published Online First: Epub Date]. 10.1002/pbc.22908 21298750

[60] van Dalen EC, van der Pal HJ, Kok WE, Caron HN, Kremer LC. Clinical heart failure in a cohort of children treated with anthracyclines: a long-term follow-up study. Eur J Cancer 2006;42(18):3191–8. 10.1016/j.ejca.2006.08.005. [published Online First: Epub Date]. 10.1016/j.ejca.2006.08.005 16987655

[61] Burke GAA, Minard-Colin V, Auperin A, et al. Dose-Adjusted Etoposide, Doxorubicin, and Cyclophosphamide With Vincristine and Prednisone Plus Rituximab Therapy in Children and Adolescents With Primary Mediastinal B-Cell Lymphoma: A Multicenter Phase II Trial. J Clin Oncol 2021;39(33):3716–24. 10.1200/JCO.21.00920. [published Online First: Epub Date]. 10.1200/JCO.21.00920 PMC9150887 34570655

[62] El Amrousy D, El-Afify D, Khedr R, Ibrahim AM. Omega 3 fatty acids can reduce early doxorubicin-induced cardiotoxicity in children with acute lymphoblastic leukemia. Pediatr Blood Cancer 2022;69(7):e29496. 10.1002/pbc.29496. [published Online First: Epub Date]. 10.1002/pbc.29496 34842343

[63] Katzenstein HM, Malogolowkin MH, Krailo MD, et al. Doxorubicin in combination with cisplatin, 5-flourouracil, and vincristine is feasible and effective in unresectable hepatoblastoma: A Children's Oncology Group study. Cancer 2022;128(5):1057–65. 10.1002/cncr.34014. [published Online First: Epub Date]. 10.1002/cncr.34014 PMC9066555 34762296

[64] Linares Ballesteros A, Sanguino Lobo R, Villada Valencia JC, et al. Early-onset Cardiotoxicity assessment related to anthracycline in children with leukemia. A Prospective Study. Colomb Med (Cali) 2021;52(1):e2034542. 10.25100/cm.v52i1.4542. [published Online First: Epub Date]. 10.25100/cm.v52i1.4542 PMC8054707 33911320

[65] Moyo D, Chimalizeni Y, Chagaluka G, Banda CG, Molyneux EM. Early doxorubicin cardiotoxicity in Malawian children admitted to Queen Elizabeth Central Hospital, Malawi. Pediatr Blood Cancer 2021;68(7):e29003. 10.1002/pbc.29003. [published Online First: Epub Date]. 10.1002/pbc.29003 33719197

[66] Samosir SM, Utamayasa IKA, Andarsini MR, et al. Risk Factors of Daunorubicine Induced Early Cardiotoxicity in Childhood Acute Lymphoblastic Leukemia: A Retrospective Study. Asian Pac J Cancer Prev 2021;22(5):1407–12. 10.31557/APJCP.2021.22.5.1407. [published Online First: Epub Date]. 10.31557/APJCP.2021.22.5.1407 PMC8408392 34048168

[67] Tan VZZ, Chan NM, Ang WL, Mya SN, Chan MY, Chen CK. Cardiotoxicity After Anthracycline Chemotherapy for Childhood Cancer in a Multiethnic Asian Population. Front Pediatr 2021;9:639603 . 10.3389/fped.2021.639603. [published Online First: Epub Date]. 10.3389/fped.2021.639603 PMC7888269 33614560

[68] Tang Y, Luo C, Shen S, et al. The efficacy and safety of a homoharringtonine-based protocol for children with acute myeloid leukemia: A retrospective study in China. Pediatr Hematol Oncol 2021;38(2):97–107. 10.1080/08880018.2020.1820649. [published Online First: Epub Date]. 10.1080/08880018.2020.1820649 33016804

[69] Tringale KR, Modlin LA, Sine K, Forlenza CJ, Cahlon O, Wolden SL. Vital organ sparing with proton therapy for pediatric Hodgkin lymphoma: Toxicity and outcomes in 50 patients. Radiother Oncol 2022;168:46–52. 10.1016/j.radonc.2022.01.016. [published Online First: Epub Date]. 10.1016/j.radonc.2022.01.016 PMC9446376 35101461

[70] Yu H, Qiu Y, Yu H, et al. Anthracycline Induced Cardiac Disorders in Childhood Acute Lymphoblastic Leukemia: A Single-Centre, Retrospective, Observational Study. Front Pharmacol 2021;12:598708. 10.3389/fphar.2021.598708. [published Online First: Epub Date]. 10.3389/fphar.2021.598708 PMC8039458 33854429

[71] Kremer LC, Bastiaansen BA, Offringa M, et al. Troponin T in the first 24 hours after the administration of chemotherapy and the detection of myocardial damage in children. Eur J Cancer 2002;38(5):686–9. 10.1016/s0959-8049(01)00431-2. [published Online First: Epub Date]. 10.1016/s0959-8049(01)00431-2 11916551

[72] Pourier MS, Mavinkurve-Groothuis AMC, Dull MM, et al. Myocardial 2D Strain During Long-Term (>5 Years) Follow-Up of Childhood Survivors of Acute Lymphoblastic Leukemia Treated With Anthracyclines. Am J Cardiol 2020;127:163–68. 10.1016/j.amjcard.2020.03.040. [published Online First: Epub Date]. 10.1016/j.amjcard.2020.03.040 32444028

[73] Merkx R, Leerink JM, de Baat EC, et al. Asymptomatic systolic dysfunction on contemporary echocardiography in anthracycline-treated long-term childhood cancer survivors: a systematic review. J Cancer Surviv 2022;16(2):338–52. 10.1007/s11764-021-01028-4. [published Online First: Epub Date]. 10.1007/s11764-021-01028-4 PMC8964593 33772445

[74] Liu J, Banchs J, Mousavi N, et al. Contemporary Role of Echocardiography for Clinical Decision Making in Patients During and After Cancer Therapy. JACC Cardiovasc Imaging 2018;11(8):1122–31. 10.1016/j.jcmg.2018.03.025. [published Online First: Epub Date]. 10.1016/j.jcmg.2018.03.025 30092969

[75] Plana JC, Galderisi M, Barac A, et al. Expert consensus for multimodality imaging evaluation of adult patients during and after cancer therapy: a report from the American Society of Echocardiography and the European Association of Cardiovascular Imaging. Eur Heart J Cardiovasc Imaging 2014;15(10):1063–93. 10.1093/ehjci/jeu192. [published Online First: Epub Date]. 10.1093/ehjci/jeu192 PMC4402366 25239940

[76] Armstrong GT, Joshi VM, Ness KK, et al. Comprehensive Echocardiographic Detection of Treatment-Related Cardiac Dysfunction in Adult Survivors of Childhood Cancer: Results From the St. Jude Lifetime Cohort Study. J Am Coll Cardiol 2015;65(23):2511–22. 10.1016/j.jacc.2015.04.013. [published Online First: Epub Date]. 10.1016/j.jacc.2015.04.013 PMC4539123 26065990

[77] Atkinson TM, Li Y, Coffey CW, et al. Reliability of adverse symptom event reporting by clinicians. Qual Life Res 2012;21(7):1159–64. 10.1007/s11136-011-0031-4. [published Online First: Epub Date]. 10.1007/s11136-011-0031-4 PMC3633532 21984468

[78] Feijen EL, van der Pal HJ, van Dalen EC, et al. A new method to facilitate valid and consistent grading cardiac events in childhood cancer survivors using medical records. PLoS One 2014;9(7):e100432. 10.1371/journal.pone.0100432. [published Online First: Epub Date]. 10.1371/journal.pone.0100432 PMC4090125 25006805

[79] Leerink JM, de Baat EC, Feijen EAM, et al. Cardiac Disease in Childhood Cancer Survivors: Risk Prediction, Prevention, and Surveillance: JACC CardioOncology State-of-the-Art Review. JACC CardioOncol 2020;2(3):363–78. 10.1016/j.jaccao.2020.08.006. [published Online First: Epub Date]. 10.1016/j.jaccao.2020.08.006 PMC8352294 34396245

[80] Leerink JM, Verkleij SJ, Feijen EAM, et al. Biomarkers to diagnose ventricular dysfunction in childhood cancer survivors: a systematic review. Heart 2019;105(3):210–16. 10.1136/heartjnl-2018-313634. [published Online First: Epub Date]. 10.1136/heartjnl-2018-313634 30158136

[81] Franco VI, Henkel JM, Miller TL, Lipshultz SE. Cardiovascular effects in childhood cancer survivors treated with anthracyclines. Cardiol Res Pract 2011;2011:134679. 10.4061/2011/134679. [published Online First: Epub Date]. 10.4061/2011/134679 PMC3038566 21331374

[82] Lipshultz SE, Lipsitz SR, Mone SM, et al. Female sex and higher drug dose as risk factors for late cardiotoxic effects of doxorubicin therapy for childhood cancer. N Engl J Med 1995;332(26):1738–43. 10.1056/NEJM199506293322602. [published Online First: Epub Date]. 10.1056/NEJM199506293322602 7760889

