# Supplementary material for: Acute and early-onset cardiotoxicity in children and adolescents with cancer: a systematic review
Source: BMC Cancer. 2023 Sep 14;23:866. doi: 10.1186/s12885-023-11353-9 (PMC10500898; doi:10.1186/s12885-023-11353-9)
Supplement: Supplementary file 1 — Additional file 1. Search strategy. [file 12885_2023_11353_MOESM1_ESM.docx]

**Additional file 1: Search strategy**

| 1. **Children:**   infan* OR newborn* OR new-born* OR perinat* OR neonat* OR baby OR baby* OR babies OR toddler* OR minors OR minors* OR boy OR boys OR boyfriend OR boyhood OR girl* OR kid OR kids OR child OR child* OR children* OR schoolchild* OR schoolchild OR school child[tiab] OR school child*[tiab] OR adolescen* OR juvenil* OR youth* OR teen* OR under*age* OR pubescen* OR pediatrics[mh] OR pediatric* OR paediatric* OR peadiatric* OR school [tiab] OR school*[tiab] OR prematur* OR preterm*  *Reference: Leclercq E, Leeflang MMG, van Dalen EC, Kremer LCM. Validation of Search Filters for Identifying Pediatric Studies in PubMed. J Pediatr. 2013;162(3):629-34.* |
| --- |
| 1. **Childhood cancer:**   leukemia OR leukemi* OR leukaemi* OR (childhood ALL) OR AML OR lymphoma OR  lymphom* OR hodgkin OR hodgkin* OR T-cell OR B-cell OR non-hodgkin OR sarcoma OR  sarcom* OR sarcoma, Ewing's OR Ewing* OR osteosarcoma OR osteosarcom* OR wilms  tumor OR wilms* OR nephroblastom* OR neuroblastoma OR neuroblastom* OR  rhabdomyosarcoma OR rhabdomyosarcom* OR teratoma OR teratom* OR hepatoma OR  hepatom* OR hepatoblastoma OR hepatoblastom* OR PNET OR medulloblastoma OR  medulloblastom* OR PNET* OR neuroectodermal tumors, primitive OR retinoblastoma OR  retinoblastom* OR meningioma OR meningiom* OR glioma OR gliom* OR pediatric  oncology OR paediatric oncology OR childhood cancer OR childhood tumor OR childhood  tumors OR brain tumor* OR brain tumour* OR brain neoplasms OR central nervous system  neoplasm OR central nervous system neoplasms OR central nervous system tumor* OR  central nervous system tumour* OR brain cancer* OR brain neoplasm* OR intracranial  neoplasm* OR leukemia lymphocytic acute OR leukemia, lymphocytic, acute[mh] |
| 1. **Cancer:**   cancer OR cancers OR cancer* OR oncology OR oncolog* OR neoplasm OR neoplasms OR  neoplasm* OR carcinoma OR carcinom* OR tumor OR tumour OR tumor* OR tumour* OR  tumors OR tumours OR malignan* OR malignant OR hematooncological OR hemato  oncological OR hemato-oncological OR hematologic neoplasms OR hematolo* |
| 1. **Anthracyclines:**   anthracyclines OR anthracyclin* OR idarubicin OR idarubic* OR epirubicin OR epirubic* OR adriamycin OR doxorubicin OR doxorubic* OR adriamyc* OR daunorubicin OR daunorubic* OR daunoxome OR doxil OR caelyx OR myocet |
| 1. **Mitoxantrone:**   mitoxantrone OR novantrone OR dihydroxyanthracenedione OR mitoxantr* |
| 1. **Radiotherapy involving the heart:**   (Radiotherapy OR radiation OR radiation therapy OR irradiation OR irradiat* OR radiation injuries OR injuries, radiation OR injury, radiation OR radiation injury OR radiation syndrome OR radiation syndromes OR syndrome radiation OR radiation sickness OR radiation sicknesses OR sickness radiation OR radiation* OR irradiation OR radiations) AND (chest OR lung OR axilla OR mediastinal OR mantle OR supraclavicular OR susclavicular OR cranial axis OR total axis OR supra diaphragm[tiab] OR abdominal OR Inverted Y[tiab] OR Left Flank OR Hemiabdomen OR Left upper quadrant OR Paraaortic OR Spleen OR craniospinal OR TBI OR Total body OR whole body OR total body* OR body whole*) |
| 1. **Cardiomyopathy/heart failure:**   (Heart/adverse effects[Mesh] OR Heart/toxicity[Mesh] OR Ventricular Dysfunction[Mesh] OR Cardiotoxicity[Mesh] OR cardiotoxicit* OR ejection fraction OR LVEF OR shortening fraction OR fractional shortening OR contractilit* OR Cardiomyopathies[Mesh] OR cardiomyopath* OR ((cardiac OR myocard* OR heart OR ventricular OR systolic) AND (damage OR injur* OR toxicit* OR disease* OR dysfunct* OR function OR strain)) OR Echocardiography[Mesh] OR echocardiograph* OR (Magnetic Resonance Imaging[mesh] AND (heart OR cardiac)) OR Congestive heart failure OR CHF OR heart failure OR clinical heart failure OR clinical cardiotoxicity OR clinical cardiotoxicities OR clinical cardiotoxicit* OR Cardiac Failure OR Myocardial Failure OR Heart Failure, Left-Sided OR Heart Failure, Left Sided OR Left-Sided Heart Failure OR Left Sided Heart Failure OR Heart Failure, Right-Sided OR Heart Failure, Right Sided OR Right-Sided Heart Failure OR Right Sided Heart Failure OR Heart Failure, Congestive OR Heart Decompensation OR Decompensation, Heart OR cardiomyopathy OR cardiomyopathies OR cardiomyopath* OR cardiac damage OR cardiac toxicity OR cardiac toxicities OR cardiac dysfunction OR cardiac dysfunctions OR cardiac failure OR cardiac failures OR heart pathology OR heart/radiation effects OR heart ventricles/radiation effects OR heart disease OR heart diseases) |
| 1. **Biomarkers:**   (Natriuretic Peptides[Mesh] OR Atrial natriuretic factor OR ANP OR ANF OR atrial natriuretic peptide OR Brain natriuretic peptide OR BNP OR Pro-brain natriuretic peptide OR N-terminal pro-BNP OR NTproBNP OR NT-proBNP OR proBNP OR ctnt OR ctni OR troponin*) |
| 1. **ECG:**   "Electrocardiography"[Mesh] OR electrocardio*[tiab] OR ecg[tiab] OR ekg[tiab] |

**1 AND (2 OR 3) AND (4 OR 5 OR 6) AND (7 OR 8 OR 9)**
