# Supplementary material for: Acute and early-onset cardiotoxicity in children and adolescents with cancer: a systematic review
Source: BMC Cancer. 2023 Sep 14;23:866. doi: 10.1186/s12885-023-11353-9 (PMC10500898; doi:10.1186/s12885-023-11353-9)
Supplement: Supplementary file 2 — Additional file 2. Risk of bias assessment criteria for observational studies. [file 12885_2023_11353_MOESM2_ESM.docx]

**Additional file 2: Risk of bias assessment criteria for observational studies**

|  | **Internal validity** | **External validity** |
| --- | --- | --- |
| **Study group** | **Selection Bias**(low risk/high risk/unclear risk):  Low risk if:  the described study group consisted of more than 90% of the childhood cancer patients treated with anthracyclines, mitoxantrone and/or radiotherapy involving the heart included in the original cohort  *or*  it was a random sample of these patients with respect to the cancer treatment and important prognostic factors for cardiac function (i.e., age, sex, prior cardiac dysfunction, prior anthracyclines, prior mitoxantrone and/or prior radiotherapy involving the heart) | **Reporting bias**(well-defined/not well-defined):  Well-defined if:  the mean, median or range of the cumulative anthracycline, mitoxantrone and cardiac irradiation dose was mentioned and prior cardiotoxic treatment |
| **Follow-up** | **Attrition bias**(low risk/high risk/unclear risk):  Low risk if:  the outcome was assessed for more than 90% of the study group of interest | **Reporting bias**(well-defined/not well-defined):  Well-defined if:  the length of follow up was mentioned |
| **Outcome** | **Detection bias**(low risk/high risk/unclear risk):  Low risk if:  the outcome assessors were blinded to the investigated determinant | **Reporting bias**(well-defined/not well-defined):  Well-defined if:  the method of detection and the definition of an abnormal outcome were provided for all outcomes reported in the study |
| **Risk estimation** | **Confounding**(low risk/high risk/unclear risk):  Low risk if:  important prognostic factors (i.e. age, sex, prior cardiac dysfunction, prior anthracyclines, prior mitoxantrone and/or prior radiotherapy involving the heart) and follow-up period were adequately taken into account | **Risk estimation analyses**(well-defined/not well-defined):  Well-defined if:  a risk ratio, odds ratio, attributable risk, linear or logistic regression model, mean difference or Chi^2^ was calculated |
