# Supplementary material for: Acute and early-onset cardiotoxicity in children and adolescents with cancer: a systematic review
Source: BMC Cancer. 2023 Sep 14;23:866. doi: 10.1186/s12885-023-11353-9 (PMC10500898; doi:10.1186/s12885-023-11353-9)
Supplement: Supplementary file 3 — Additional file 3. Data extraction forms. [file 12885_2023_11353_MOESM3_ESM.docx]

**Additional file 3: Data extraction forms**

| **What is the frequency of occurrence of acute and early-onset cardiotoxicity, as diagnosed by clinical, echocardiographic and biochemical parameters routinely used in clinical practice, in children with cancer treated with anthracyclines, mitoxantrone and/or radiotherapy involving the heart? What are the risk factors?** | | | | |
| --- | --- | --- | --- | --- |
| *Agha et al: Early Ventricular Dysfunction After Anthracycline Chemotherapy in Children. Pediatric Cardiology, 2016; 37:537-544.* | | | | |
| **Study design; treatment era; follow-up** | **Participants** | **Treatment** | **Diagnostic test; main outcomes** | **Risk of bias assessment** |
| Study design:  Cross-sectional prospective study (single-center)  Treatment era:  Not reported  Follow-up:  From before induction to 1 week after last dose of induction chemotherapy; no further information provided | Type and number of participants:  Original cohort unclear, described study group  N = 30 asymptomatic newly diagnosed children with hematological malignancies  Diagnosis:  N = 12/30 (40%) Non-Hodgkin lymphoma (NHL)  N = 8/30 (26.7%) Acute myeloid leukemia (AML)  N = 7/30 (23.3%) Acute lymphoblastic leukemia (ALL)  N = 3/30 (10%) Hodgkin disease (HD)  Age at cancer diagnosis:  Mean 9.24 years ± 4.14 (SD)  Gender:  Male N = 21/30 (70%)  Female N = 9/30 (30%)  Controls:  Not applicable  Cardiovascular risk factors:  Not reported  Prior cardiotoxic treatment:  Not reported  Prior cardiac dysfunction:  Not reported  Prior cardioprotective interventions:  Not reported | Anthracyclines:  Dose according to protocol, actual received cumulative dose not described.  Doxorubicin:  N = 12/30 (40%) NHL: 60mg/m^2^ (60mg/m^2^ at day 2)  N = 8/30 (26,7%) AML: 75mg/m^2^ (25mg/m^2^ at day 3, 4 and 5)  N = 7/30 (23,3%) ALL: 50mg/m^2^ (25mg/m^2^ at day 1 and 8)  N = 3/30 (10%) HD: 50mg/m^2^ (25mg/m^2^ at day 1 and 15)  Infusion duration not reported.  Mitoxantrone:  Not reported  Radiotherapy involving the heart:  Not reported | Diagnostic test used for cardiotoxicity assessment:  Transthoracic echocardiography; conventional echo-doppler measures, tissue doppler imaging and 2D- speckle tracking echocardiography (2D-STE)  Timing of the diagnostic test:  Before induction and 1 week after last dose of induction treatment  Outcome definitions:  *Continuous parameters:*  No definition of abnormal values provided: **fractional shortening**; **ejection fraction**; **global longitudinal strain**; **tricuspid E’/A’ ratio**; **right ventricle Tei-index**  *Dichotomous parameters:* **Fractional shortening below normal reference range (28-38%)**; **decrease in fractional shortening >10% of baseline value**  Outcome assessors blinded:  Not reported  Occurrence of acute and early-onset cardiotoxicity (not separated): *Continuous parameters (mean ± SD):*  - **Fractional shortening** decreased after chemotherapy (38.70 ± 3.93 vs. 36.00 ± 5.00%, *P* <0.01)  - **Ejection fraction** decreased after chemotherapy (70.60 ± 5.70 % vs. 66.00 ± 7.18%, *P* <0.01)  - The average **global longitudinal strain** significantly decreased after chemotherapy (-21.58 ± 2.54 vs. -19.18 ± 3.59%, *P* = 0.001)  - **Tricuspid E’/A’ ratio** significantly decreased after chemotherapy (1.29 ± 0.27 vs. 1.03 ± 0.37, *P* <0.01)  - **Right ventricle Tei-index** showed significant prolongation after chemotherapy 0.32 ± 0.06 vs. 0.36 ± 0.08, *P* <0.01)  *Dichotomous parameters:*  - **Fractional shortening below normal reference range (28-38%)** N = 2/30 (6.7%)  - **Decrease in fractional shortening >10% of baseline value** N = 12/30 (40%)  Mortality: Not reported  Risk factors assessed:  No  Results of multivariate analyses:  Not applicable | Selection bias: Unclear risk (original cohort of eligible participants unclear)  Attrition bias: Low risk  (all participants had an outcome assessment for all outcomes)  Detection bias: Unclear risk (not described if assessors were blinded)  Confounding: Not applicable  Reporting bias (study group): Not well-defined (not reported whether doxorubicin doses were received per protocol or were adjusted per patient, no cumulative dosage anthracyclines; no information on mitoxantrone and radiotherapy involving the heart provided)  Reporting bias (follow-up): Not well-defined (length of follow-up is unclear)  Reporting bias (outcome): Well defined (method of detection and definition abnormal outcome provided) for dichotomous outcomes. Not well-defined for continuous outcomes (no definition abnormal outcome provided)  Risk estimation analyses:  Not applicable  Funding of the trial: No funding  Overlap with other included studies:  Not presumed |

| **What is the frequency of occurrence of acute and early-onset cardiotoxicity, as diagnosed by clinical, echocardiographic and biochemical parameters routinely used in clinical practice, in children with cancer treated with anthracyclines, mitoxantrone and/or radiotherapy involving the heart ? What are the risk factors?** | | | | |
| --- | --- | --- | --- | --- |
| *Al-Biltagi et al.: Strain Echocardiography in Early Detection of Doxorubicin-Induced Left Ventricular Dysfunction in Children with Acute Lymphoblastic Leukemia. ISRN Pediatrics, 2012; Volume 2012, Article ID 870549: 9 pages.* | | | | |
| **Study design; treatment era; follow-up** | **Participants** | **Treatment** | **Diagnostic test; main outcomes** | **Risk of bias assessment** |
| Study design: Single-center prospective cohort study  Treatment era: March 2008 – March 2010  Follow-up:  From before doxorubicin treatment and within 1 week of starting Doxorubicin treatment. | Type and number of participants: Described study group N = 25 children with newly diagnosed acute lymphoblastic leukemia.  Original cohort unknown.  Diagnosis: N = 25 acute lymphoblastic leukemia  Age at cancer diagnosis: Mean 9 years (± 2.6 SD)  Gender: Male N = 13/25 (52%)  Female N= 12/25 (48%)  Controls:  N = 30 healthy children matched of age and sex, mean age 9.2 years ± 2.9 SD  - Male N = 14/30 (46,7%)  - Female N = 16/30 (53,3%)  Cardiovascular risk factors:  ‘Any associated systemic disease that can affect the cardiac function’ and ‘medication that can affect cardiac function, such as angiotensin-converting enzyme inhibitors, angiotensin receptor blockers, diuretics or beta-blockers’ mentioned as exclusion criteria  Prior cardiotoxic treatment:  Not applicable (excluded)  Prior cardiac dysfunction:  Not applicable (excluded)  Prior cardioprotective interventions:  Not reported | Anthracyclines:  Dose according to protocol, actual received cumulative dose not described.  Doxorubicin, intravenous, 30 mg/m^2^, days 8, 15, 22, 29.  Infusion duration not reported.  Mitoxantrone:  Not reported  Radiotherapy involving the heart:  Not reported | Diagnostic test used for cardiotoxicity assessment:  - Echocardiographic Doppler examination  - Measurement of troponin I (cTnI) and creatine phosphokinase CPK (MB) levels  Timing of the diagnostic test: Before doxorubicin treatment and within 1 week of starting Doxorubicin treatment  Outcome definitions:  *Numerous continuous parameters:* **fractional shortening**, **E/A ratio**, **global peak systolic strain**, **troponin I**, **CPK**; no definition of abnormal values reported.  Outcome assessors blinded:  No (‘echocardiograms were not blindly read’)  Occurrence of acute cardiotoxicity:  *Only continuous parameters (mean ±SD):*  - **Fractional shortening** significantly decreased after doxorubicin (40 ± 4.87 vs 33.5 ± 6.58, *P* = 0.02)  - **E/A ratio** showed no significant change after doxorubicin (1.60 ± 0.42 1.5 ± 0.37, *P* = 1.06)  - **Global peak systolic strain** showed significantly reduction after doxorubicin (−18.65 ± 4.52% vs −15.10 ± 2.45%, *P* = 0.04)  - **Troponin I** (ng/ml) showed a significant increase after doxorubicin (0.055 ± 0.003 vs 0.061 ± 0.005, *P* = 0.002)  - **CPK** (MB) (U/L) showed no significant increase after doxorubicin (50.60 ± 8.55 vs 48.61 ± 6.56, *P* = 0.62)  Occurrence of early-onset cardiotoxicity:  - Not reported (shorter follow-up period)  Mortality: Not reported  Risk factors assessed:  No  Results of multivariate analyses:  Not applicable | Selection bias: Unclear risk (original cohort of eligible patients unclear)  Attrition bias: Low risk (all participants had an outcome assessment for all outcomes)  Detection bias: High risk for echocardiographic parameters (outcome assessors not blinded), low risk for biomarkers (no information on blinding of outcome assessors provided, but as blinding is not relevant for outcomes diagnoses by laboratory tests we judged this outcome at low risk of detection bias)  Confounding: Not applicable  Reporting bias (study group): Not well-defined (not reported whether doxorubicin doses were received per protocol or were adjusted per patient, no cumulative dosage anthracyclines; no information on mitoxantrone and radiotherapy involving the heart provided)  Reporting bias (follow-up): Well-defined (length of follow-up is reported)  Reporting bias (outcome):  Not well defined (definition of abnormal outcome not described)  Risk estimation analyses:  Not applicable  Funding of the trial:  Not reported  Overlap with other included studies:  Presumably same study group as El-Shitany 2012 (same authors, same study period, same center, same group size and diagnosis) |

| **What is the frequency of occurrence of acute and early-onset cardiotoxicity, as diagnosed by clinical, echocardiographic and biochemical parameters routinely used in clinical practice, in children with cancer treated with anthracyclines, mitoxantrone and/or radiotherapy involving the heart? What are the risk factors?** | | | | |
| --- | --- | --- | --- | --- |
| *Asselin et al:* *Cardioprotection and Safety of Dexrazoxane in Patients Treated for Newly Diagnosed T-Cell Acute Lymphoblastic Leukemia or Advanced-Stage Lymphoblastic Non-Hodgkin Lymphoma: A Report of the Children’s Oncology Group Randomized Trial Pedatric Oncoloy Group 9404. Journal of Clinical Oncology, 2016; Volume 34 (number 8): pages 854 – 862.* | | | | |
| **Study design; treatment era; follow-up** | **Participants** | **Treatment** | **Diagnostic test; main outcomes** | **Risk of bias assessment** |
| Study design: Randomized controlled trial, but as only one arm was eligible for this review this study was seen as a prospective cohort study  Treatment era:  June 1996 – September 2001  Follow-up:  Median follow-up = 8.9 years (range 0.02 – 14.7), starting point and end point of follow-up not defined. | Type and number of participants:  N = 264 patients with newly diagnosed T-cell acute lymphoblastic leukemia (ALL) or lymphoblastic non-Hodgkin lymphoma (L-NHL) allocated to treatment with doxorubicin but no dexrazoxane  Original cohort unknown, N = 573 patients enrolled, N = 35 did not meet inclusion criteria, N = 1 not evaluable for response  N = 537 eligible for randomization, N = 273 allocated to treatment with doxorubicin and dexrazoxane.  Information provided in this table is for the patients treated with doxorubicin but no dexrazoxane.  Diagnosis:  - T-cell acute lymphoblastic leukemia N = 175/264 (66.3%)  - Lymphoblastic non-Hodgkin lymphoma N = 89/264 (33.7%)  Age at cancer diagnosis:  Mean 9.7 years (SD 4.58)  Gender:  Male: N = 203/264 (76.9%) Female: N = 61/264 (23.1%)  Controls:  Not applicable  Cardiovascular risk factors:  Not reported  Prior cardiotoxic treatment:  Emergency mediastinal radiation (treatment for severe respiratory distress), number not reported  Prior cardiac dysfunction:  N = 5/97 (5.2%) with a troponin evaluation at baseline had elevated troponin.  No other parameters reported.  Prior cardioprotective interventions:  Not reported | Anthracyclines:  Dose according to protocol, actual received cumulative dose not described.  Induction doxorubicin 30mg/m^2^ (day 1, 2, 22), consolidation doxorubicin 30mg/m^2^ (every 3 weeks) to a cumulative dose of 360mg/m^2^. Infusion duration not reported.  Mitoxantrone:  Not reported  Radiotherapy involving the heart:  Number not reported | Diagnostic test used for cardiotoxicity assessment:  - Serum cardiac troponin-T concentrations (cTnT)  - Echocardiography  Timing of the diagnostic test:  - cTnT measures at diagnosis, before doxorubicin (cumulative) doses of 120, 240, and 330 mg/m^2^, and 3 weeks after last dose of doxorubicin (median 21 days (range 1 to 571 days) after start treatment)  - Echocardiography before doxorubicin treatment (diagnosis), at week 4 of induction, 3 weeks after completion doxorubicin therapy (week 34), and 3 and 6 years from diagnosis (i.e., 1 and 4 years from end of chemotherapy)  Outcome definitions:  *Continuous parameters:*  **Left ventricular fractional shortening** z-score (abnormal values not defined)  *Dichotomous parameters:*  - **Acute cardiac toxicity:** NCI Common Terminology Criteria for Adverse Effect, version 2.0, cardiac disorders grade 3 or higher  - **Elevated serum cTnT** = >0,01 ng/ml  Outcome assessors blinded: Yes (both)  Central investigators who evaluated troponin and echocardiograms were blinded to patient clinical status and treatment assignment.  Occurrence of acute and early-onset cardiotoxicity (not separated): *Continuous parameters:*  **Left ventricular fractional shortening** (z-score):  - Baseline (N = 158/264 with outcome assessment), z-score 0.37  - End of doxorubicin treatment (N = 61/264 with outcome assessment), z-score -1.68  *Dichotomous parameters:*  - **Grade 3 or 4 cardiac toxicity** (while receiving therapy) N = 3 with decreased left ventricular fractional shortening (number with outcome assessment not mentioned)  - **Elevated serum cTnT** N = 10/114 (8.8%)  (N = 114/264 with outcome assessment at a median of 21 days after start of treatment)  Mortality:  N = 35/264 (13.3%) died within 2 years from diagnosis  Risk factors assessed:  No  Results of multivariate analyses:  Not applicable | Selection bias: Unclear risk (original cohort unknown)  Attrition bias:  cTnT measures high risk, outcome assessed for <90% of study group:  Baseline = 97/264 (36.7%)  3 weeks after start treatment = 114/264 (43.2%)  Echocardiography (continuous parameters) high risk, outcome assessed for <90% of study group, different parameters not specified.  Baseline = 158/264 (59.9%)  Approximately week 34 = 61/264 (23.1%)  Cardiac toxicity (LVSF), unclear risk, not described if the whole study group was assessed  Detection bias: Low risk for cTnT and echocardiography  (Central investigators were blinded to patient clinical status and treatment assignment), unclear risk for cardiac toxicity (not reported if investigators were blinded)  Confounding: Not applicable  Reporting bias (study group): Not well defined (not reported whether doxorubicin doses were received per protocol or were adjusted per patient, no cumulative dosage anthracyclines; no information on mitoxantrone and radiotherapy involving the heart provided)  Reporting bias (follow-up): Well-defined (length of follow-up mentioned)  Reporting bias (outcome):  Well defined for dichotomous outcomes (method and definition are provided)  Not well-defined for continuous outcomes (no definition provided)  Risk estimation analyses:  Not applicable  Funding of the trial:  No specific funding reported, supported by Michael J. Borowith  Overlap with other included studies:  Not presumed |

| **What is the frequency of occurrence of acute and early-onset cardiotoxicity, as diagnosed by clinical, echocardiographic and biochemical parameters routinely used in clinical practice, in children with cancer treated with anthracyclines, mitoxantrone and/or radiotherapy involving the heart? What are the risk factors?** | | | | |
| --- | --- | --- | --- | --- |
| *Berrak et al: Doxorubicin cardiotoxicity in children: Reduced incidence of cardiac dysfunction associated with continuous-infusion schedules. Oncology reports 8, 2001; pages 611-614.* | | | | |
| **Study design; treatment era; follow-up** | **Participants** | **Treatment** | **Diagnostic test; main outcomes** | **Risk of bias assessment** |
| Study design: Single center retrospective cohort study  Treatment era:  August 23, 1988 – July 2, 1998  Follow-up:  Median 30.5 months (2 – 122 months). | Type and number of participants:  97 children who had been treated with continuous infusion of doxorubicin at a dosage of 60mg/m^2^ over 24 hours or at a dosage of 75mg/m^2^ over 72 hours.  Original cohort unknown  Diagnosis:  N = 87/97 (89.7%) Osteosarcoma  N = 2/97 (2.1%) Ewing’s sarcoma  N = 1/97 (1.0%) Neuroblastoma  N = 2/97 (2.1%) Nasopharyngeal carcinoma  N = 1/97 (1.0%) Desmoid fibroma  N = 1/97 (1.0%) Rhabdomyosarcoma  N = 1/97 (1.0%) Fibrosarcoma  N = 2/97 (2.1%) Wilms’ tumor  Age at cancer diagnosis:  Median 13 years ± 4 (SD)  Gender:  Male N = 59/97 (60.1%)  Female N = 38/97 (39.1%)  Controls:  Historical data for similar patients treated with doxorubicin administered by rapid infusion (not further described)  Cardiovascular risk factors:  ‘Neither cardiac status nor risk factors affected protocol selection’  Prior cardiotoxic treatment:  Not described  Prior cardiac dysfunction:  ‘None of these patients had evidence of pre-existing cardiac disease or cardiac dysfunction’  Prior cardioprotective interventions:  Not reported | Anthracyclines:  - N = 61/97 (62.9%) Doxorubicin 60mg/m^2^ every three weeks by continuous infusion over 24h (mean cumulative dosage ± SD, 324 ± 97 mg/m^2^)  - N = 36/97 (37.1%) Doxorubicin 75mg/m^2^ every three weeks by continuous infusion over 72h (mean cumulative dosage ± SD, 423±65 mg/m^2^)  Total doxorubicin dose ranged from 90 to 455 mg/m^2^ (mean ± SD, 360±98mg/m^2^)  Mitoxantrone:  Not reported  Radiotherapy involving the heart:  ‘None received radiation to the precordium’ | Diagnostic test used for cardiotoxicity assessment:  Clinical examination, evaluation by M-mode and two-dimensional echocardiography  Timing of the diagnostic test:  As a baseline and follow-up every six months  Outcome definitions:  Cardiac dysfunction defines as a decrease in the ejection fraction of more than 15% from the baseline value  Outcome assessors blinded:  Not reported  Occurrence of acute cardiotoxicity:  Not reported  Occurrence of early-onset cardiotoxicity:  N = 1/97 (1.0%) decrease in ejection fraction (and death due to progressive heart failure).  Mortality:  N = 37/97 (38.1%) died from their respective malignant conditions (not specified at which point during follow-up).  Risk factors assessed: No  Results of multivariate analyses:  Not applicable | Selection bias: Unclear risk (original cohort of eligible participants is unclear)  Attrition bias: Low risk (outcome assessed for whole study group)  Detection bias:  Unclear risk (not described if assessors were blinded)  Confounding: Not applicable  Reporting bias (study group): Well defined (cumulative dosage doxorubicin, mean + SD, described)  Reporting bias (follow-up): Well defined (length of follow-up mentioned)  Reporting bias (outcome): Well defined (method of detection and definition abnormal outcome provided)  Risk estimation analyses:  Not applicable  Funding of the trial:  Not reported  Overlap with other included studies:  Not presumed |

| **What is the frequency of occurrence of acute and early-onset cardiotoxicity, as diagnosed by clinical, echocardiographic and biochemical parameters routinely used in clinical practice, in children with cancer treated with anthracyclines, mitoxantrone and/or radiotherapy involving the heart? What are the risk factors?** | | | | |
| --- | --- | --- | --- | --- |
| *Brown et al: Anthracycline Induced Cardiac Toxicity in Pediatric Ewing Sarcoma: A Longitudinal Study. Pediatric Blood Cancer, 2013; 60: pages 842-848.* | | | | |
| **Study design; treatment era; follow-up** | **Participants** | **Treatment** | **Diagnostic test; main outcomes** | **Risk of bias assessment** |
| Study design:  Single center retrospective cohort study  Treatment era:  1978 - 2006  Follow-up:  Median time of follow up = 88 months (range 98-304). Duration of follow-up from baseline to at least 5 years after diagnosis. | Type and number of participants:  N = 71 patients with newly diagnosed Ewing sarcoma from one large geographic area (British Columbia)  Original cohort N = 91 cases; 20 excluded: N = 9 primary treatment not in BC, N = 1 no anthracyclines, N = 6 no baseline echo, N = 3 incomplete anthracycline data, N = 1 early disease death  Diagnosis:  N = 71 Ewing sarcoma  Age at cancer diagnosis:  Median age 11 years (2.2 – 16.1)  Gender:  Male 34/71 (47.9%)  Female 37/71 (52.1%)  Controls:  Not applicable  Cardiovascular risk factors:  Not reported  Prior cardiotoxic treatment:  Not applicable  Prior cardiac dysfunction:  No patients (excluded)  Prior cardioprotective interventions:  ‘Dexrazoxane was not administered to any of these patients’ | Anthracyclines:  Doxorubicin:  N = 2/71 (2.8%) 60mg/m^2^ every 6 weeks, IV bolus  N = 12/71 (16.9%) 75mg/m^2^ every 3 weeks, IV bolus)  N = 22/71 (31.0%) 75mg/m^2^ every 6 weeks, IV bolus  N = 1/71 (1.4%) 35mg/m^2^ every 2 weeks, IV bolus  N = 1/71 (1.4%) 30mg/m2/day every 6 weeks, 72 hours infusion  N = 7/71 (9.9%) 37.5mg/ m^2^ x2 days every 3 weeks, 48 hours infusion  N = 22/71 (31.0%) 37.5mg/m^2^ x2 days every 6 weeks, 48 hours infusion  N = 4/71 (5.6%) 20mg/m^2^ x3 days every 6 weeks, 4-6 hours infusion  Median dose of anthracycline 365mg/m^2^ (113-1,025).  N = 6 <250mg/m^2^  N = 10 ≤300mg/m^2^  N = 19 <350mg/m^2^  N = 41 ≤375mg/m^2^  Median duration anthracycline therapy = 6.5 months (0.82-19.5)  Mitoxantrone:  Not reported  Radiotherapy involving the heart:  N = 6/71 (8.5%) radiotherapy involving the heart, median dose of radiation 5,000 cGy.  N = 2/6 (33.3%) patients receiving radiation >50% heart volume | Diagnostic test used for cardiotoxicity assessment:  Echocardiograms  Timing of the diagnostic test:  Five time points: pretreatment, worst function during treatment, at completion of therapy, worst function during surveillance, most recent echocardiogram prior to January 1, 2011.  Median echocardiogram review period 5 years (range 2 months to 25.2 years)  Outcome definitions:  Cardiotoxicity:  - Ejection fraction <50% **(Common Terminology Criteria for Adverse Events (CTCAE) v3.0 and v4.0, grade 2 and higher)**  - Ejection fraction decline from baseline >10% from baseline **(CTCAE v4.0, grade 2 and higher)**  Outcome assessors blinded:  Not reported (unknown)  Occurrence of acute and early-onset cardiotoxicity (not separated):  **CTCAE v3.0 during or on completion of chemotherapy**:  - Grade 0, N = 26/71 (36.6%)  - Grade 1, N = 25/71 (35.2%)  - Grade 2, N = 10/71 (14.1%)  - Grade 3, N = 5/71 (7.0%)  - Missing, N = 5/71 (7.0%)  **CTCAE v4.0 during therapy**:  - 10-19% decline from baseline ejection fraction, N = 10/71 (14.1%)  Mortality: N = 20/71 (28.2%) died within 10 years from diagnosis  Risk factors assessed:  Yes, but not for acute and early-onset cardiotoxicity seperately    Results of multivariate analyses:  Not applicable | Selection bias: High risk (study group comprised N = 71/91 (78.0%) of original cohort)  Attrition bias: Low risk for CTCAE v3.0 during or on completion of chemotherapy (outcome assessment available in 66/71 (93.0%) of study group). Unclear risk for CTCAE v4.0 during therapy (number in which outcome was assessed not mentioned)  Detection bias: Unclear risk (blinding not stated)  Confounding: Not applicable  Reporting bias (study group): Well-defined (dosage of anthracyclines well described, as well as dosage changes and radiotherapy involving the heart)  Reporting bias (follow-up): Well-defined (follow-up period mentioned)  Reporting bias (outcome): Well-defined (method of detection and definition of abnormal outcome were provided)  Risk estimation analyses: Not applicable  Funding of the trial:  Not reported  Overlap with other included studies:  Not presumed |

| **What is the frequency of occurrence of acute and early-onset cardiotoxicity, as diagnosed by clinical, echocardiographic and biochemical parameters routinely used in clinical practice, in children with cancer treated with anthracyclines, mitoxantrone and/or radiotherapy involving the heart? What are the risk factors?** | | | | |
| --- | --- | --- | --- | --- |
| *Burke et al.: Dose-Adjusted Etoposide, Doxorubicin, and Cyclophosphamide With Vincristine and Prednisone Plus Rituximab Therapy in Children and Adolescents With Primary Mediastinal B-Cell Lymphoma: A Multicenter Phase II trial. Journal of Clinical Oncology, 2021; volume 39, issue 33: pages: 3716 - 3725* | | | | |
| **Study design; treatment era; follow-up** | **Participants** | **Treatment** | **Diagnostic test; main outcomes** | **Risk of bias assessment** |
| Study design:  Single-arm phase II trial (academic international study)  Treatment era:  April 2012 and April 2016  Follow-up:  Median follow-up 59.0 months (IQR 52.6 – 69.2)  From start of therapy, endpoint not specified | Type and number of participants:  N = 48 children between 6 months to 18 years with newly diagnosed primary mediastinal large B-cell lymphoma (PMLBL)  Original cohort unclear  N = 2 excluded (N = 1 diagnosis Burkitt leukemia who did not receive any trial therapy and N = 1 diagnosis Hodgkin lymphoma)  **N = 46 eligible patients**  Diagnosis:  All patients were diagnosed with PMLBL on local pathologic review, national pathologic review:  N = 41/46 (89.1%) PMLBL  N = 1/46 (2.2%) diffuse large B-cell lymphoma (DLBCL)  N = 1/46 (2.2%) gray zone lymphoma  N = 3/46 (6.5%) national pathologic review not done  Age at cancer diagnosis:  Median age 15.4 years, range 7-17, (IQR 14-16 years)  Distribution:  7 to <12 years N = 4/46 (8.7%)  12 to <15 years N = 15/46 (32.6%)  15 to <18 years N = 27/46 (58.7%)  Gender:  Male N = 20/46 (43.5%)  Female N = 26/46 (56.5%)  Controls:  Not applicable  Cardiovascular risk factors:  Not reported  Prior cardiotoxic treatment:  Not reported  Prior cardiac dysfunction:  Not reported (not described as exclusion criteria)  Prior cardioprotective interventions:  Not reported | Anthracyclines:  N = 46 patients received six courses of a dose adjusted etoposide, doxorubicin, cyclophosphamide with vincristine and prednisone plus rituximab (DA-EPOCH-R) regimen. Per protocol, 40mg/m^2^ doxorubicin administered in first cycle, dose in following cycles escalated based on absolute neutrophile counts  N = 33/46 (71.7%) received ≥300mg/m^2^ cumulative dose of doxorubicin  N = 11/46 (23.9%) received ≥350mg/m^2^ cumulative dose of doxorubicin.  (N = 42/46, 91.3% patients followed the dose escalation rules according to available data)  Infusion duration not reported.  Mitoxantrone:  None  Radiotherapy involving the heart:  Not reported | Diagnostic test used for cardiotoxicity assessment:  Not specified (assuming clinical evaluation and echocardiography)  Timing of the diagnostic test: Not specified (during treatment, cycles of DA-EPOCH-R) and 1 year after treatment)  Outcome definitions:  Adverse events (AEs) graded according to NCI-CTC V4.  Cardiac AE grade 2-5.  Abnormal left ventricular ejection fraction or abnormal left ventricular shortening fraction not specified.  Outcome assessors blinded: Not reported  Occurrence of acute cardiotoxicity:  Not separated from early-onset cardiotoxicity  Occurrence of early-onset cardiotoxicity:  N = 1/46 (2.2%) left ventricular systolic dysfunction (grade 2) after fifth cycle of therapy).  N = 1/26 patients evaluated at 1 year after last chemotherapy) (3.8%) had cardiac toxicity with a shortening fraction of 26%.  Mortality:  N = 7/46 (15.2%) deaths during follow up (N=6 progression, N=1 second malignancy)  N = 0/46 (0%) toxic deaths  During follow-up of 4 years, therefore not specified for early-onset mortality.  Risk factors assessed: No  Results of multivariate analyses: Not applicable | Selection bias: Unclear risk (original cohort unclear)  Attrition bias: Low risk for cardiotoxicity evaluation during treatment (all AE’s were assessed in all patients and all 276 courses). High risk for cardiotoxicity evaluation at 1 year (26/36 (72.2%) had an evaluation (echocardiography) at 1 year following the last chemotherapy)  Detection bias: Unclear risk (blinding of assessors not described)  Confounding: Not applicable  Reporting bias (study group): Not well-defined (no mean of cumulative dose given, no infusion duration described)  Reporting bias (follow-up): Well-defined (length of follow-up provided)  Reporting bias (outcome):  Not well-defined (method of detection partly described, definition of abnormal outcome provided for NCI-CTC criteria, but not provided for abnormal ventricular function (ejection fraction or shortening fraction))  Risk estimation analyses:  Not applicable  Funding of the trial:  Supported by the Clinical Research Hospital Program of the French Ministry of Health, Enfants Cancers Santé (ECS), NCTN Operations Center Grant U10Ca180886, NCTN Statistics & Data Center Grant U10XA180899, Cancer Research UK, and National Institute for Health Research Clinical Research Network (UK).  Overlap with other included studies:  Not presumed |

| **What is the frequency of occurrence of acute and early-onset cardiotoxicity, as diagnosed by clinical, echocardiographic and biochemical parameters routinely used in clinical practice, in children with cancer treated with anthracyclines, mitoxantrone and/or radiotherapy involving the heart? What are the risk factors?** | | | | |
| --- | --- | --- | --- | --- |
| *Chen et al: Present Risk of Anthracycline or Radiation-induced Cardiac Sequelae Following Therapy of Malignancies in Children and Adolescents. Klinische Paediatrie, 2009; volume 221/3: pages 162-6.* | | | | |
| **Study design; treatment era; follow-up** | **Participants** | **Treatment** | **Diagnostic test; main outcomes** | **Risk of bias assessment** |
| Study design:  Single center retrospective cohort study.  Treatment era:  2000 - 2004  Follow-up:  Mean follow-up 5.6 years (range 3.2 – 8 years) after successful completed chemotherapy.  Starting point = prior to chemotherapy.  Follow-up = after successful completion of therapy at routine outpatient referrals (most commonly 6-12 month intervals after chemotherapy). | Type and number of participants:  N = 168 patients treated for malignancies with anthracyclines.  Original cohort unknown.  Diagnosis:  N = 64/168 (38.1%) acute lymphatic leukemia (ALL)  N = 26/168 (15.5%) non-Hodgkin lymphoma (NHL)  N = 9/168 (5.4%) acute myeloic leukemia (AML)  N = 25/168 (14.9%) Hodgkin’s disease (HD)  N = 10/168 (6.0%) osteosarcoma (OS)  N = 9/168 (5.4%) Ewing tumor (ET)  N = 5/168 (3.0%) nephroblastoma (NBL)  N = 12/168 (7.1%) neuroblastoma (NB)  N = 8/168 (4.8%) other  Age at cancer diagnosis:  Mean age 8.1 years (±5.3 years, ranging 1 month to 19.6 years)  Gender:  Male = 110/168 (65.5%)  Female = 58/168 (34.5%)  Controls:  Not applicable  Cardiovascular risk factors:  Not reported  Prior cardiotoxic treatment:  Not reported  Prior cardiac dysfunction:  Not reported  Prior cardioprotective interventions:  Not reported | Anthracyclines:  Used anthracyclines and standard cumulative dose used according to protocol:  ALL; N = 64/168 (38.1%) adriamycine 60mg/m^2^ and daunorubicine 144mg/m^2^ (low risk) OR adriamycine 120mg/m^2^ and daunorubicine 144mg/m^2^ (high risk)  HL; N = 26/168 (15.5%) adriamycine 120mg/m^2^ and daunorubicine 144mg/m^2^ (CoALL) OR adriamycine 100mg/m^2^ (BFM)  AML; N = 9/168 (5.4%) Idarubicin 64mg/m^2^  HD; N = 25/168 (14.9%) adriamycine 160mg/m^2^  OS; N = 10/168 (6.0%) adriamycine 360mg/m^2^ (standard risk 1) OR adriamycine 270mg/m^2^ (standard risk 2)  ET; N = 9/168 (5.4%) adriamycine 360mg/m^2^  NBL; N = 5/168 (3.0%) adriamycine 250mg/m^2^ (standard risk) OR adriamycine 300mg/m^2^ (high risk)  NB; N = 12/168 (7.1%) adriamycine 180mg/m^2^  Other; N = 8/168 (4.8%) adriamycine 240mg/m2 OR adriamycine 150mg/m2. Infusion duration not reported.  Mitoxantrone:  AML; N = 9/168 (5.4%) Mitoxantrone 20mg/m^2^  Radiotherapy involving the heart:  N = 18/25 (72.0%) of patients with Hodgkin disease treated with additional chest radiation.  Mean dose of 22 ± 4.26 Gy of the heart included in the radiation field.  N = 1/18 (5.6%) developed acute left ventricular systolic dysfunction shortly after radiation | Diagnostic test used for cardiotoxicity assessment:  Echocardiographic studies (M-mode, two dimensional, Doppler)  Timing of the diagnostic test:  Prior (1-2 days) to each cycle of chemotherapy. Follow-up after successful completion of therapy at routine outpatient referrals (most commonly 6-12 month intervals after chemotherapy).  Outcome definitions:  Adverse cardiac effect =  Fractional shortening below 28%  Outcome assessors blinded:  Not reported  Occurrence of acute and early-onset cardiotoxicity (not separated):  N = 2/168 (1.2%) fractional shortening below 28%.  - 1/2 (50.0%) at completion of radiation and chemotherapy  - 1/2 (50.0%) concurrent with atrial ectopic tachycardia during chemotherapy  Mortality: Not reported  Risk factors assessed: No  Results of multivariate analyses:  Not applicable | Selection bias: Unclear risk (original cohort not described)  Attrition bias: Low risk (all included patients are assessed for outcome)  Detection bias: Unclear risk (blinding is not mentioned)  Confounding: Not applicable  Reporting bias (study group): Not well-defined (Unclear how many patients were in the low- or high risk groups, meaning it not being clear which dosage of anthracyclines they received. Moreover, no cumulative dose reported, only per protocol).  Reporting bias (follow-up): Well-defined (length of follow-up mentioned)  Reporting bias (outcome):  Well defined (method of detection and definition of abnormal outcome provided)  Risk estimation analyses: Not applicable  Funding of the trial:  No funding reported  Overlap with other included studies:  Not presumed |

| **What is the frequency of occurrence of acute and early-onset cardiotoxicity, as diagnosed by clinical, echocardiographic and biochemical parameters routinely used in clinical practice, in children with cancer treated with anthracyclines, mitoxantrone and/or radiotherapy involving the heart? What are the risk factors?** | | | | |
| --- | --- | --- | --- | --- |
| *Cheung et al: Circulating high-sensitivity troponin T and microRNAs as markers of myocardial damage during childhood leukaemia treatment. Pediatric Research, 2020;* 89, pages 1245–1252*.* | | | | |
| **Study design; treatment era; follow-up** | **Participants** | **Treatment** | **Diagnostic test; main outcomes** | **Risk of bias assessment** |
| Study design:  Single center prospective cohort study  Treatment era:  Not reported  (published in 2020)  Follow-up:  About 16 months (patients were followed from diagnosis (starting point, before starting chemotherapy) up to 6 months after completion of anthracycline therapy (end point), not further described) | Type and number of participants:  N = 39 children newly diagnosed with acute lymphoblastic or myeloid leukemia  N = 1 did not complete the study due to parental withdrawal.  Original cohort unknown.  Diagnosis:  N = 32/39 (82.1%) acute lymphoblastic leukemia  N = 7/39 (17.9%) acute myeloid leukemia  Age at cancer diagnosis:  Mean age 7.5 years ± 4.8 (SD)  Gender:  Male = 25/39 (64.1%)  Female = 14/39 (35.9%)  Controls:  Not applicable  Cardiovascular risk factors:  Not applicable  Prior cardiotoxic treatment:  Not reported  Prior cardiac dysfunction:  None reported (N=0), patients were excluded  ‘Exclusion criteria included patients with congenital heart disease and those with heart failure prior to initiation of chemotherapy’  Prior cardioprotective interventions:  Not reported | Anthracyclines:  Anthracycline therapy not defined. Dosage and infusion duration of anthracyclines not mentioned.  Mitoxantrone:  Not reported  Radiotherapy involving the heart:  Not reported | Diagnostic test used for cardiotoxicity assessment:  Echocardiographic assessment and blood investigations (hs-cTnT and circulating miRNAs levels)  Timing of the diagnostic test:  - Baseline before start of chemotherapy (TP0);  - Within 24 hours after given the first dose of anthracycline (TP1);  - Within 1 week after completion of the anthracycline therapy in induction fase (TP2);  - Within 1 week after completion of all anthracycline treatment (TP3);  - 6 months after completion of anthracycline treatment (TP4).  Outcome definitions:  *Continuous parameters:*  **- Plasma high-sensitivity cardiac troponin T (hs-cTnT)**, not defined  - **Global longitudinal myocardial deformation (systolic strain**), not defined  *Dichotomous parameters:*  - **Clinical heart failure or reduced LV ejection fraction** (not defined).  - **Subclinical myocardial dysfunction** = ≥20% reduction of LV global systolic longitudinal strain.  Outcome assessors blinded:  Not reported  Occurrence of acute cardiotoxicity:  *Continuous parameters:*  - **hs-cTnT** not reported  - **Global longitudinal myocardial deformation: Systolic strain (%, mean ± SD)**  TP0 17.9 ± 1.6  TP1 15.7 ± 2.1  *Dichotomous parameters:*  - **Clinical heart failure / reduced LV ejection fraction**, N = 0/39 (0%)  Occurrence of early-onset cardiotoxicity:  *Continuous parameters:*  - **hs-cTnT** (mean ± SD) was significantly higher at TP2 (13.03 ± 14.96 ng/l), TP3 (11.53 ± 8.52 ng/l) and TP4 (4.87 ± 3.04 ng/l) when compared with TP0: (3.50 ± 5.44 ng/l), all *P* <  0.001  - **Global longitudinal myocardial deformation: Systolic strain (%, mean ± SD)**  TP0 17.9 ± 1.6  TP2 13.7 ± 2.0 (*P* <0.05, TP2 versus TP0)  TP3 15.4 ± 1.7  TP4 16.8 ± 1.8  *Dichotomous parameters:*  - **Clinical heart failure/ reduced LV ejection fraction** N = 0/39 (0%)  - **Subclinical myocardial dysfunction** at TP2, N = 22/39 (56.4%)  Mortality: N = 4/39 (10.3%) died during treatment due to relapse, refractoriness to treatment or infection. N = 1/4 (25.0%) between TP1 and TP2, N = 2/4 (50.0%) between TP2 and TP3, N = 1/4 (25.0%) between TP3 and TP4  Risk factors assessed: No  Results of multivariate analyses:  Not applicable | Selection bias: Unclear risk (original cohort unclear)  Attrition bias: Low risk (39/40 = 97.5% of study group were assessed)  Detection bias: Unclear risk (blinding not mentioned)  Confounding: Not applicable  Reporting bias (study group): Not well-defined (specifics, cumulative dose or range of dose of anthracyclines not mentioned, radiotherapy not reported)  Reporting bias (follow-up): Well-defined (length of follow-up defined, however, no mean of follow-up mentioned)  Reporting bias (outcome):  Well-defined for subclinical myocardial dysfunction (method of detection and definition of abnormal outcome provided), not well-defined for hs-cTnT, global longitudinal myocardial deformation, clinical heart failure or reduced left ventricle ejection fraction (method of detection defined, no definition of abnormal outcome provided)  Risk estimation analyses:  Not applicable  Funding of the trial:  Supported by the Health and Health Services Research Fund, Food and Health Bureau, Hong Kong SAR Government.  Overlap with other included studies:  Not presumed |

| **What is the frequency of occurrence of acute and early-onset cardiotoxicity, as diagnosed by clinical, echocardiographic and biochemical parameters routinely used in clinical practice, in children with cancer treated with anthracyclines, mitoxantrone and/or radiotherapy involving the heart? What are the risk factors?** | | | | |
| --- | --- | --- | --- | --- |
| *Choi et al.: Dexrazoxane for Preventing Anthracycline Cardiotoxicity in Children with Solid Tumors. Korean Medical Science, 2010; 25: 1336-1342* | | | | |
| **Study design; treatment era; follow-up** | **Participants** | **Treatment** | **Diagnostic test; main outcomes** | **Risk of bias assessment** |
| Study design: Single center retrospective cohort study  Treatment era:  January 1995 – July 2001.  Follow-up:  Median follow up 86 months (range 7-158) for the control group.  Starting point = diagnosis, before first gift of doxorubicin.  End point = not specified | Type and number of participants:  Total study group = 103 patients diagnosed as having solid tumors.  Original cohort unknown.  N = 47 patients in dexrazoxane group (3 patients excluded from dexrazoxane group because of cardiac dysfunction before doxorubicin therapy)  N = 42 historical control group treated with the same chemotherapy as the study group (11 patients excluded from control group (1 misdiagnosis, 2 incomplete records, 3 early withdrawals without echocardiographic evaluation, 3 congenital heart diseases, 3 cardiac dysfunction))  Information provided in this table is for the patients treated with doxorubicin but no dexrazoxane (also called historical group).  Diagnosis:  Neuroblastoma, N = 40/42 (95.2%)  Peripheral primitive neuro-ectodermal tumor, N = 2 /42 (4.8%)  Age at cancer diagnosis:  Median age = 30 months (range 3-127)  Gender:  Male = 25/42 (59.5%)  Female = 17/42 (40.5%)  Controls:  Not applicable  Cardiovascular risk factors:  Not reported  Prior cardiotoxic treatment:  Not reported  Prior cardiac dysfunction:  None (patients with cardiac dysfunction before start of therapy were excluded)  Prior cardioprotective interventions:  Not reported | Anthracyclines:  Doxorubicin, intravenous as bolus infusion, dose 30mg/m^2^ at intervals of 4 weeks  Cumulative dose of doxorubicin 266.1mg/m^2^ SD ±75.0 (range 87-388; median 294)  Mitoxantrone:  None  Radiotherapy involving the heart:  N = 3/42 (7.1%) patients receiving chest therapy  Cardioprotective interventions:  Not applicable for historical group. | Diagnostic test used for cardiotoxicity assessment:  Echocardiography  Timing of the diagnostic test:  Before initiation of doxorubicin therapy; every 2-3 doses of doxorubicin before cumulative dose reached of 300mg/m^2^; afterwards every dose of doxorubicin administration; after completion treatment on a yearly basis  Outcome definitions:  **Cardiac events**: at least one of the following three criteria: 1) increased LV diastolic diameter for the age; 2) increased LV systolic diameter for the age; 3) FS less than 28% at any time point of doxorubicin treatment, either **subclinical or asymptomatic cardiotoxicity** (based on changes in FS and LV diameters) or **clinical cardiotoxicity** (based on the presence of heart failure symptoms)  **Early cardiotoxicity**: cardiac toxicity during or within one year after doxorubicin treatment  Outcome assessors blinded:  Not reported  Occurrence of acute and early-onset cardiotoxicity (not separated):  Early cardiotoxicity  N = 16/42 (38.1%)  Mortality:  N = 2/42 (4.8%) cardiac deaths (not specified at which point during follow-up).  Risk factors assessed:  Yes, but not in multivariate analysis  Results of multivariate analyses:  Not applicable | Selection bias: Unclear risk (unclear how historical control group was selected)  Attrition bias:  Unclear risk (not specified in which proportion of study group outcome was assessed)  Detection bias: Unclear risk (blinding not reported)  Confounding: Not applicable  Reporting bias (study group): Well defined (mean and cumulative dose of doxorubicin mentioned)  Reporting bias (follow-up): Well defined (length of follow-up mentioned)  Reporting bias (outcome):  Well-defined for all outcomes  Risk estimation analyses: Not applicable (no multivariate analysis)  Funding of the trial: Not reported  Overlap with other included studies:  Not presumed |

| **What is the frequency of occurrence of acute and early-onset cardiotoxicity, as diagnosed by clinical, echocardiographic and biochemical parameters routinely used in clinical practice, in children with cancer treated with anthracyclines, mitoxantrone and/or radiotherapy involving the heart? What are the risk factors?** | | | | |
| --- | --- | --- | --- | --- |
| *Creutzig et al.: Longitudinal Evaluation of Early and Late Anthracycline Cardiotoxicity in Children with AML. Pediatric Blood Cancer, 2007: 48: pages 651 - 662* | | | | |
| **Study design; treatment era; follow-up** | **Participants** | **Treatment** | **Diagnostic test; main outcomes** | **Risk of bias assessment** |
| Study design:  Retrospective cohort study  Treatment era:  January 1993 – June 2003  Follow-up:  From start therapy to after (not specified) | Type and number of participants:  N = 1207 patients <18 years with acute myelogeneous leukemia (AML) (N=1010 de novo AML patients without Down syndrome, N=121 de novo AML patients with Down syndrome, N=76 patients with secondary AML after other malignancies).  ‘Five patients were excluded from the analysis. One of them with a congenital heart defect had a decreased shortening fraction (SF) grade 2 already at diagnosis, which remained at the same level during anthracycline therapy until death after early relapse. Another four patients died during intensive chemotherapy or after stem cell transplantation (64-288 days after diagnosis) suffering from cardiomyopathy, which was attributable to simultaneous sepsis (three patients) or to other therapy-related complications (ileus, pulmonary oedema, one patient). All five patients had failed to achieve remission.’  Data for early cardiotoxicity available for 885/1207 (73.3%). Because of our research question, we will focus on this group from here on.  Diagnosis:  N = 790/885 (89.3%) de novo AML  N = 74/885 (8.4%) DS-AML  N = 21/885 (2.4%) secondary AML  Age at cancer diagnosis:  Not reported  Gender:  Not reported  Controls:  Not applicable  Cardiovascular risk factors:  Not reported  Prior cardiotoxic treatment:  Not reported  Prior cardiac dysfunction:  Not reported (one patient was excluded because of congenital heart defect, but no exclusion criteria are mentioned)  Prior cardioprotective interventions:  Not reported | Anthracyclines:  N = 402/885 (45.4%) **AML-BFM93**  Cumulative anthracycline dose standard risk group = 300mg/m^2^, high risk group 400mg/m^2^.  Induction = 2x30 mg/m^2^/day daunorubicin (DNR) x 3 days or 12 mg/m^2^ idarubicin (IDR) 30 minute infusion x 3 days.  Since 1996 4 hour infusion DNR or IDR.  N = 483 /885 (54.6%) **AML-BFM98**  Cumulative anthracycline dose cycle therapy 450mg/m^2^ and 6-week consolidation 420mg/m^2^  Induction = Only IDR 12mg/m^2^ 4 hour infusion x 3 days  ‘Cumulative dose were calculated as equivalence dose to daunorubicin (DNR) using a dose ratio 1:5 for idarubicin and mitoxantrone.’  Mitoxantrone:  N = 402/885 (45.4%) **AML-BFM93**  Second induction randomly assigned in the high-risk group mitoxantrone 10mg/m^2^/day (HAM) at day 3 and 4 (cumulative dose 20mg/m^2^), number of patients who received mitoxantrone not specified  N = 483 /885 (54.6%) **AML-BFM98** all children received HAM (including mitoxantrone), except AML FAB M3 and DS-AML, number of patients who received mitoxantrone not specified  Radiotherapy involving the heart:  Not reported | Diagnostic test used for cardiotoxicity assessment:  Clinical evaluation and echocardiography.  Timing of the diagnostic test:  After induction before starting HAM course, before consolidation, before HAE course, at the end of maintenance treatment and after finishing maintenance as annuals controls.  Outcome definitions:  Clinical cardiotoxicity defined as clinical signs and symptoms of cardiomyopathy not attributable to other know causes such as sepsis or renal failure.  Subclinical cardiotoxicity defined as the lack of clinical signs and symptoms of cardiomyopathy, but with an abnormal shortening fraction (SF) of the left ventricle measured by echocardiography.  Decreased SF <30% defined as abnormal.  Early cardiotoxicity defined as during intensive AML therapy or within 1 year afterwards.  Cardiac toxicity graded by WHO Common Toxicity Criteria for cardiac function and echocardiography.  Outcome assessors blinded: Not reported  Occurrence of acute cardiotoxicity:  Not separated from early-onset cardiotoxicity  Occurrence of early-onset cardiotoxicity:  Data for early cardiotoxicity available for 885/1207 (73.3%) patients  N = 38/885 (4.3%) developed **early cardiotoxicity.**  - N = 2/38 DS-AML  - N = 1/38 secondary AML)  N = 14/885 (1.6%) developed **clinical cardiotoxicity**  N = 24/885 (2.7%) developed **subclinical cardiotoxicity**  - N = 9/24 (37.5%) SF of 20-25% (grade 2)  - N = 11/24 (45.8%) SF of 25-30% (grade 1)  - N = 4/24 (16.7%) temporarily decreased SF >10% compared to baseline  **Time of occurrence**  N = 10/38 (26.3%) after induction  N = 5/38 (13.2%) after second induction with HAM  N = 15/38 (39.5%) consolidation  N = 4/38 (10.5%) 2-cycle therapy  N = 3/38 (7.9%) last block HAE  N = 1/38 (2.6%) during maintenance therapy  Mortality:  N = 5/38 (13.2%) of patients with clinical or subclinical cardiotoxicity died within 1 year after therapy  - N = 3/5 treatment-related  - N = 2/5 AML relapse/non-response  Risk factors assessed: Yes, but only for late cardiotoxicity  Results of multivariate analyses: Not applicable | Selection bias: Unclear risk (original cohort unclear)  Attrition bias: High risk (Data for early cardiotoxicity available for 885/1207 (73.3%) patients)  Detection bias: Unclear risk (no mention of blinding of assessors)  Confounding: Not applicable  Reporting bias (study group): Not well-defined (no changes in cumulative dose given, only standard protocol numbers)  Reporting bias (follow-up): Not well-defined (no length of follow-up given)  Reporting bias (outcome): Well-defined (method of detection provided and definition of abnormal outcome provided)  Risk estimation analyses: Not applicable  Funding of the trial:  Supported by Deutsche Krebshilfe e. V. and in part by Pharmaxia & Upjohn GmbH and Pfizer GmbH.  Overlap with other included studies:  Not presumed |

| **What is the frequency of occurrence of acute and early-onset cardiotoxicity, as diagnosed by clinical, echocardiographic and biochemical parameters routinely used in clinical practice, in children with cancer treated with anthracyclines, mitoxantrone and/or radiotherapy involving the heart? What are the risk factors?** | | | | |
| --- | --- | --- | --- | --- |
| *De Matos Neto et al.: Left Ventricular Systolic Function Assessed by Echocardiography in Children and Adolescents with Osteosarcoma Treated with Doxorubicin Alone or in Combination with Dexrazoxane. Arq Bras Cardiol 2006; 87(6): 699-706* | | | | |
| **Study design; treatment era; follow-up** | **Participants** | **Treatment** | **Diagnostic test; main outcomes** | **Risk of bias assessment** |
| Study design:  Prospective non-randomized study (multi-center)  Treatment era:  May 1996 – February 2001  Follow-up:  Not specified  (from start therapy to final evaluation 4 weeks after 6^th^ cycle of chemotherapy) | Type and number of participants:  Total cohort N = 55 patients with osteosarcoma undergoing a six-cycle chemotherapy regimen of doxorubicin.  Original cohort unknown.  - N = 37 /55 (67.3%) patients who did not receive dexrazoxane  - N = 18 /55 (32.7%) patients who did receive dexrazoxane  Information provided in this table is for the patients treated with doxorubicin but no dexrazoxane.  Diagnosis:  N = 37 osteosarcoma  Age at cancer diagnosis:  Average age 15.4 years  Gender:  Male N = 28/37 (75.7%)  Female N = 9/37 (24.3%)  Controls:  Not applicable  Cardiovascular risk factors:  Not applicable  Prior cardiotoxic treatment:  Not reported  Prior cardiac dysfunction:  None (N = 0, exclusion criteria)  Prior cardioprotective interventions:  Not applicable | Anthracyclines:  Doxorubicin intravenously, 6 cycles, 3 in preoperative phase and 3 in postoperative phase, in doses of 60 mg/m^2^ (in rapid infusions of 30 minutes each)  Cumulative dosage doxorubicin in mg/m^2^; mean (standard error)  - Evaluation 2 = 174.05 (8.52)  - Evaluation 3 = 292.38 (21.71)  - Evaluation 4 = 345.19 (20.3)  Mitoxantrone:  None  Radiotherapy involving the heart:  None | Diagnostic test used for cardiotoxicity assessment:  Echocardiography  Timing of the diagnostic test:  Evaluation 1: before beginning of chemotherapy  Evaluation 2: up to 2 weeks after the 3^rd^ cycle  Evaluation 3: up to 2 weeks after 5^th^ cycle  Evaluation 4: up to 4 weeks after the 6^th^ cycle of chemotherapy  Outcome definitions:  Left ventricular systolic dysfunction = fractional shortening percentage (FS%) ≤ 29% and/or a drop in FS% by an absolute value ≥ 10 units of the baseline value of each patient  Outcome assessors blinded: Yes  (Operator of echocardiography was blinded to dose of anthracycline)  Occurrence of acute and early-onset cardiotoxicity (not separated):  N = 7/37 (18.9%) had left ventricular dysfunction (altered FS%)  N = 5/7 (71.4%) progressed to normal levels before end of chemotherapy  FS%; Mean (standard error)  - Evaluation 1: 37.03 (3.66)  - Evaluation 2: 35.67 (3.84)  - Evaluation 3: 34.95 (3.79)  - Evaluation 4: 35.26 (3.91)  Mortality  Not reported  Risk factors assessed: No  Results of multivariate analyses:  Not applicable | Selection bias:  Unclear risk (original cohort of eligible participants unclear)  Attrition bias: Low risk (140/148 (94.6%) echocardiograms were performed for our patient population)  Detection bias: Low risk (Assessors for echocardiography were blinded)  Confounding: Not applicable  Reporting bias (study group): Well-defined (cumulative dosage of anthracyclines mentioned)    Reporting bias (follow-up): Well-defined (follow-up period linked to chemotherapy protocol)  Reporting bias (outcome):  Well-defined (method of detection and definition of abnormal outcome provided)  Risk estimation analyses: Not applicable  Funding of the trial:  Supported by: UNIFESP - EPM and CAPES.  Overlap with other included studies:  Not presumed |

| **What is the frequency of occurrence of acute and early-onset cardiotoxicity, as diagnosed by clinical, echocardiographic and biochemical parameters routinely used in clinical practice, in children with cancer treated with anthracyclines, mitoxantrone and/or radiotherapy involving the heart? What are the risk factors?** | | | | |
| --- | --- | --- | --- | --- |
| *El Amrousy et al.: Omega 3 fatty acids can reduce early doxorubicin-induced cardiotoxicity in children with acute lymphoblastic leukemia. Pediatric blood & Cancer, 2021; 69e29496* | | | | |
| **Study design; treatment era; follow-up** | **Participants** | **Treatment** | **Diagnostic test; main outcomes** | **Risk of bias assessment** |
| Study design:  Prospective randomized clinical trial (single center)  Treatment era:  February 2020 – August 2021  Follow-up:  From start therapy to 6 months after therapy (no mean of follow-up given) | Type and number of participants:  N = 78 assessed for eligibility (N = 18 excluded for not meeting inclusion criteria (N = 13) or declining to participate (N = 5))  N = 60 children with newly diagnosed Acute lymphoblastic leukemia (ALL)  Group 1 (N=30) received omega 3 fatty acids 1000mg/day for 6 months in addition to their usual doxorubicin protocol  Group II (N=30) received usual doxorubicin protocol  Information provided in this table is for the patients treated with doxorubicin but no omega 3 fatty acids.  Diagnosis:  ALL  Age at cancer diagnosis:  Mean age 8.5 years ± 1.9 SD  Gender:  Male N = 19/30 (63.3%) Female N = 11/30 (36.7%)  Controls: Not applicable  Cardiovascular risk factors:  Not reported  Prior cardiotoxic treatment:  None (exclusion criteria mentions ‘previous chemotherapy or radiotherapy’)  Prior cardiac dysfunction:  None (exclusion criteria mentions ‘CHF, acquired heart disease, congenital heart disease, cardiomyopathy’)  Prior cardioprotective interventions:  None (exclusion criteria mentions ‘patients receiving other drugs that may affect cardiac function’) | Anthracyclines:  Dose according to protocol, actual received cumulative dose not described.  Doxorubicin 25mg/m^2^ intravenously (days 2 and 9) in induction, not further described during consolidation/continuation phases).  Infusion duration not reported.  Mitoxantrone:  None  Radiotherapy involving the heart:  Not reported | Diagnostic test used for cardiotoxicity assessment:  Echocardiography and laboratory tests (troponin I (TnI), creatinine kinase-MB (CK-MB), N-terminal pro-brain natriuretic peptide (NT-proBNP).  Timing of the diagnostic test:  Echocardiography and laboratory tests: Before treatment and after 6 months  Outcome definitions:  Early-onset toxicity = within 1 year of ending chemotherapy  Outcome assessors blinded: For echocardiography: Yes  Occurrence of acute and early-onset cardiotoxicity (not separated): *Continuous results*  **Troponin I (ng/ml)** mean before treatment <0.01, mean after treatment 0.050 ± 0.012, *P* <0.05.  **CK-MB (U/L)** mean before treatment 11.6 ± 3.6 SD, mean after treatment 46 ± 7.2 SD, *P* <0.05.  **NT-proBNP (pg/ml)** mean before treatment 37.1 ± 7.7 SD, mean after treatment 88.8 ± 13.6 SD, *P* <0.05.  **Left ventricular fraction shortening (LV FS %)** mean before treatment 40.7 ± 5.2 SD, mean after treatment 36 ± 3.8 SD, not significant  **Mitral E/A** mean before treatment 1.49 ± 14 SD, mean after treatment 1.45 ± 1.6 SD, NS  **Two-dimensional global longitudinal strain** mean before treatment -19.8 ± 1.2 SD, mean after treatment -15.8 ± 1.6 SD, *P* <0.05  Mortality:  Not reported  Risk factors assessed: No  Results of multivariate analyses: Not applicable | Selection bias: High risk (60/78, 76.9% of original cohort included)  Attrition bias: Low risk (all patients 30/30, 100% had an result for outcome measurements)  Detection bias: Low risk for echocardiography (assessors were blinded to study groups). Low risk for laboratory tests (no blinding of assessors mentioned, but since blinding is not relevant for this outcome measurement we scores this as low risk).  Confounding: Not applicable  Reporting bias (study group): Not well-defined (no cumulative dosage of anthracyclines given, no infusion duration of doxorubicin during consolidation given)  Reporting bias (follow-up): Well-defined (6 months)  Reporting bias (outcome): Not well-defined (method of detection provided, but no definition of abnormal outcome provided)  Risk estimation analyses: Not applicable  Funding of the trial: None reported  Overlap with other included studies:  Not presumed |

| **What is the frequency of occurrence of acute and early-onset cardiotoxicity, as diagnosed by clinical, echocardiographic and biochemical parameters routinely used in clinical practice, in children with cancer treated with anthracyclines, mitoxantrone and/or radiotherapy involving the heart? What are the risk factors?** | | | | |
| --- | --- | --- | --- | --- |
| *El-Shitany et al.: Protective Effect of Carvedilol on Adriamycin-Induced Left Ventricular Dysfunction in Children With Acute Lymphoblastic Leukemia. Journal of Cardiac Failure Vol. 18 No. 8 2012.* | | | | |
| **Study design; treatment era; follow-up** | **Participants** | **Treatment** | **Diagnostic test; main outcomes** | **Risk of bias assessment** |
| Study design:  Parallel-assignment, randomized clinical study (single center).  Treatment era:  March 2008 – March 2010  Follow-up:  From before therapy to one week after last dose of ADR (not further specified) | Type and number of participants:  N = 50 children with newly diagnosed acute lymphoblastic leukemia (ALL)  Original cohort unknown.  N = 25/50 (50%) with Adriamycin (ADR)  N = 25/50 (50%) with ADR + carvedilol  Information provided in this table is for the patients treated with anthracylines but no carvedilol.  Diagnosis:  N = 25/25 (100%) ALL  Age at cancer diagnosis:  Mean age 9.5 years ± 2.6 SD  Gender:  Male N = 12/25 (48%)  Female N = 13/25 (52%)  Controls:  Not applicable  Cardiovascular risk factors:  Not reported  Prior cardiotoxic treatment:  None (exclusion criteria mentions ‘previous chemotherapy or radiotherapy’)  Prior cardiac dysfunction:  None (exclusion criteria mentions ‘any cardiac disease either congenital or acquired, any cardiac lesion detected in baseline echocardiography’)  Prior cardioprotective interventions:  Not applicable | Anthracyclines:  Dose according to protocol, actual received cumulative dose not described. Adriamycin 30mg/m^2^ (days 8, 15, 22 and 29), slow intravenous infusion over 6 hours.  Mitoxantrone:  None  Radiotherapy involving the heart:  Not reported | Diagnostic test used for cardiotoxicity assessment:  Conventional echocardiography, pulsed tissue doppler imaging, longitudinal strain echocardiography.  Laboratory tests (LDH, CPK, troponin I)  Timing of the diagnostic test:  Before and after therapy (1 week after last ADR dose)  Outcome definitions:  Fractional shortening, E/A ratio, LV global peak systolic strain and global peak systolic strain (GPSS), Troponin I, CPK. No definitions of abnormal outcomes given.  Outcome assessors blinded:  Not reported  Occurrence of acute and early-onset cardiotoxicity (not separated): *Continuous parameters:*  **Fractional shortening (FS)** before ADR therapy mean 40 ± 4.62 SD and after ADR therapy mean 33.5 ± 6.24 SD (significantly different *P* ≤0.05)  **E/A ratio** before ADR therapy mean 1.904 ± 0.403 SD and after ADR therapy mean 1.966 ± 0.389 SD  **Global peak-systolic strain** before ADR therapy mean 18.65 ± 2.9 SD and after 15.1 ± 1.769 SD (significantly different P≤0.05)  **Troponin I (ng/mL)** before ADR therapy no data, and after ADR therapy mean 0.061 ± 0.05 SD.  **CPK I (U/L)** before ADR therapy mean 50.6 ± 25.67, after ADR therapy mean 47.6 ± 20.45 SD  Mortality:  Not reported  Risk factors assessed: No  Results of multivariate analyses:  Not applicable | Selection bias:  Unclear risk (original cohort unclear)  Attrition bias: Low risk (All included patients had outcomes for both echocardiography and laboratory tests)  Detection bias:  Unclear risk (blinding not mentioned) for echocardiography.  Low risk for laboratory tests (No information on blinding of outcome assessors provided for laboratory results, but as blinding is not relevant for outcomes diagnosed by laboratory tests we judged this outcome at low risk of detection bias).  Confounding: Not applicable  Reporting bias (study group): Not well-defined (no cumulative dosage of anthracyclines given)  Reporting bias (follow-up): Not well-defined (No mean/SD given for length of follow-up)  Reporting bias (outcome):  Not well-defined (method of detection given but no definition of abnormal outcome given)  Risk estimation analyses:  Not applicable  Funding of the trial:  Not reported  Overlap with other included studies:  Presumably same study group as Al-Biltagi 2012 (same authors, same study period, same center, same group size and diagnosis) |

| **What is the frequency of occurrence of acute and early-onset cardiotoxicity, as diagnosed by clinical, echocardiographic and biochemical parameters routinely used in clinical practice, in children with cancer treated with anthracyclines, mitoxantrone and/or radiotherapy involving the heart? What are the risk factors?** | | | | |
| --- | --- | --- | --- | --- |
| *Erkus et al.: Early prediction of anthracycline induced cardiotoxicity. Acta Pædiatrica, 2006, 96, pp. 506–509* | | | | |
| **Study design; treatment era; follow-up** | **Participants** | **Treatment** | **Diagnostic test; main outcomes** | **Risk of bias assessment** |
| Study design:  Prospective single center cohort study  Treatment era:  Not reported  Follow-up:  From start therapy to one month after end of therapy, not further specified | Type and number of participants:  N = 29 patients with diagnosis acute lymphoblastic leukemia and who received anthracycline containing chemotherapy.  Original cohort unknown.  Diagnosis:  Acute lymphoblastic leukemia  Age at cancer diagnosis:  Mean age = 6.65 years ± 0.625 SD (range 1-16)  Gender:  Male N = 21/29 (72.4%)  Female N = 8/29 (27.6%)  Controls:  Not applicable  Cardiovascular risk factors:  Not reported  Prior cardiotoxic treatment:  Not reported  Prior cardiac dysfunction:  Not reported  ‘At the start of anthracycline therapy all patients had normal systolic function prior to receiving chemotherapy (EF 75.20% ± 0.90, FS 39.6% ± 0.47)’  Prior cardioprotective interventions:  Not reported | Anthracyclines:  ALL-BFM95 treatment protocol.  N = 16/29 (55.2%) doxorubicin + daunorubicin  N = 11/29 (37.9%) daunorubicin only  N = 1/29 (3.4%) doxorubicin + idarubycine  N = 1/29 (3.4%) doxorubicin only  ‘Mean ± SD value of drug doses given = 181.6 ± 64.9mg/m^2^ (range 56-374mg/m^2^)’  N = 1/29 (3.4%) anthracycline dose >300mg/m^2^  Infusion duration not reported.  Mitoxantrone:  None  Radiotherapy involving the heart:  None (exclusion criteria mentions ‘those receiving radiotherapy were excluded from the study’) | Diagnostic test used for cardiotoxicity assessment:  Echocardiography, blood samples for serum BNP (brain natriuretic peptide), cTnI (serum cardiac troponin I) and TAOS (total antioxidant status)  Timing of the diagnostic test:  At the start of therapy and one month after end of anthracycline administration  Outcome definitions:  Echocardiography:  - **Left ventricular failure** = Ejection Fraction (EF) < 55% OR Fractional shortening (FS) <29%  Blood samples:  - **BNP** reference values are 10.3 ± 11.6 ng/L  - **cTnI** reference values are <0.04 ng/mL  Outcome assessors blinded:  For echocardiography yes. For laboratory results not described.  Occurrence of acute cardiotoxicity:  Not applicable  Occurrence of early-onset cardiotoxicity:  *Dichotomous parameters*  N = 0/29 (0%) **Left ventricular failure**  N = 2/29 (6.9%) **elevated cTnI** (0.06 and 0.14 ng/mL), but EF and FS in normal range  *Continuous parameters*  **Echocardiography** (mean ± SD):  - EF decreased from 75.20 ± 0.90 to 68.4 ± 4.8  - FS decreased from 39.6 ± 2.9 to 36.6 ± 2.9  - Both within normal limits, but significantly lower (*P* <0.05)  - With every 1 mg/m^2^ increase in drug dosage, an 0.0355 decrease in value of EF and 0.0225 decrease in value of FS (P<0.05)  Laboratory (mean ± SD)  - **BNP** significantly increased from 4.09 ± 2.26 pg/mL to 7.47 ± 3.16 pg/mL (*P* <0.05) (within reference range)  - BNP values increased 0.0242 pg/mL with every 1 mg/m^2^ increase in anthracycline dosage (*P* <0.05)  - **cTnI** not significantly changed (from 0.020 ± 0.006 ng/mL to 0.024 ± 0.009 ng/mL)  Mortality:  Not reported  Risk factors assessed: No  Results of multivariate analyses: Not applicable | Selection bias:  Unclear risk (original cohort not specified)  Attrition bias:  Low risk, all included patients are assessed for all outcomes (echocardiography and laboratory results)  Detection bias: Low risk, assessors for echocardiography were blinded. No information on blinding provided for assessors of laboratory results, but as blinding is not relevant for outcomes diagnosed by laboratory tests we judged this outcome at low risk of detection bias  Confounding: Not applicable  Reporting bias (study group): Not well-defined, mean dosage anthracycline given (but different kinds of anthracyclines used, not specified)  Reporting bias (follow-up): Not well-defined, length of follow-up not specified (no mean/range)  Reporting bias (outcome): Well-defined, method of detection and definition of abnormal outcome provided  Risk estimation analyses: Not applicable  Funding of the trial: Not reported  Overlap with other included studies:  Not presumed |

| **What is the frequency of occurrence of acute and early-onset cardiotoxicity, as diagnosed by clinical, echocardiographic and biochemical parameters routinely used in clinical practice, in children with cancer treated with anthracyclines, mitoxantrone and/or radiotherapy involving the heart? What are the risk factors?** | | | | |
| --- | --- | --- | --- | --- |
| *Fukumi et al.: Longitudinal evaluation of anthracycline cardiotoxicity by signal-averaged electrocardiography in children with cancer. Pediatrics International, 2002 (44), pages 134–140* | | | | |
| **Study design; treatment era; follow-up** | **Participants** | **Treatment** | **Diagnostic test; main outcomes** | **Risk of bias assessment** |
| Study design:  Prospective single center cohort study  Treatment era:  Not reported  Follow-up:  From start therapy up to having received maximum anthracycline dosage.  ‘Duration from onset to investigation of cardiotoxicity was 6 months to 4 years and 3 months’, presumably this indicates the last investigation of cardiotoxicity, i.e. the end of follow-up; but this is not clearly stated | Type and number of participants:  N = 29 patients with cancer with anthracycline usage.  Original cohort unknown.  Diagnosis:  N = 18/29 (62.1%) Acute lymphoblastic leukemia (ALL)  N = 6/29 (20.7%) Acute non-lymphoblastic leukemia (ANLL)  N = 3/29 (10.3%) Malignant lymphoma  N = 2/29 (6.9%) Ewing’s sarcoma  Age at cancer diagnosis:  Range of age = 5 months to 15 years and 2 months  *Age groups:*  N = 15/29 (51.7%) >4 years old  N = 6/29 (20.7%) 2-4 years old  N = 8/29 (27.6%) <2 years old  Gender:  Male N = 16/29 (55.2%)  Female N = 13/29 (44.8%)  Controls:  Not applicable  Cardiovascular risk factors: Not reported  Prior cardiotoxic treatment: Not reported  Prior cardiac dysfunction: ‘None of the patients had symptoms of congestive heart failure’  Prior cardioprotective interventions:  Not reported | Anthracyclines:  Most patients received combinations of anthracyclines or anthracyclines and mitoxantrone  **Doxorubicin** N = 13/29 (44.8%), median cumulative dosage 120 mg/m^2^, range 50-400 mg/m^2^  **Aclarubicin** N = 4/29 (13.8%), median cumulative dosage 120 mg/m^2^, range 50-180 mg/m^2^  **Daunorubicin** N = 14/29 (48.3%), median cumulative dosage 80 mg/m^2^, range 25-180 mg/m^2^  **Epirubicin** N = 1/29 (3.4%), cumulative dosage 460mg/m^2^  **Pirarubicin** N = 13/29 (44.8%), median cumulative dosage 100 mg/m^2^, range 60-360 mg/m^2^  **Idarubicin** N = 4/29 (13.8%), median cumulative dosage 49.5 mg/m^2^, range 24-100 mg/m^2^  Mitoxantrone:  N = 9/29 (31.0%), median cumulative dosage 45 mg/m^2^, range 20-120 mg/m^2^  Radiotherapy involving the heart:  None had irradiation of the chest | Diagnostic test used for cardiotoxicity assessment:  Echocardiography  Timing of the diagnostic test:  Repeatedly, 1) a few days after each anthracycline course (defined as early cardiotoxicity) and 2) just before the next anthracycline course (defined as chronic cardiotoxicity)  Outcome definitions:  Ejection fraction (EF) <50% is considered abnormal  Outcome assessors blinded: Not reported  Occurrence of acute cardiotoxicity:  There were no patients with abnormal EF  Occurrence of early-onset cardiotoxicity:  There were no patients with abnormal EF  Mortality  Not reported  Risk factors assessed:  No  Results of multivariate analyses:  Not applicable | Selection bias: Unclear risk (original cohort unclear)  Attrition bias: Low risk (all outcomes assessed in whole study group)  Detection bias: Unclear risk (blinding not stated)  Confounding: Not applicable  Reporting bias (study group): Well-defined (cumulative dosage of anthracyclines and mitoxantrone mentioned for all patients).  Reporting bias (follow-up): Not well-defined (length of follow-up not clearly defined)  Reporting bias (outcome): Well-defined (method of detection and definition of abnormal outcome provided)  Risk estimation analyses: Not applicable  Funding of the trial:  Not reported  Overlap with other included studies:  Not presumed |

| **What is the frequency of occurrence of acute and early-onset cardiotoxicity, as diagnosed by clinical, echocardiographic and biochemical parameters routinely used in clinical practice, in children with cancer treated with anthracyclines, mitoxantrone and/or radiotherapy involving the heart? What are the risk factors?** | | | | |
| --- | --- | --- | --- | --- |
| *Getz et al.: Occurrence of Treatment-Related Cardiotoxicity and Its Impact on Outcomes Among Children Treated in the AAML0531 Clinical Trial: A Report From the Children’s Oncology Group. Journal of clinical oncology, 2018; volume 37, issue 1: pages 12-21.* | | | | |
| **Study design; treatment era; follow-up** | **Participants** | **Treatment** | **Diagnostic test; main outcomes** | **Risk of bias assessment** |
| Study design:  Multi-center retrospective cohort study.  Treatment era:  August 2006 – June 2010  Follow-up:  Median follow-up of 6.6 years (range 0 to 9.8 years) for patients alive at last contact  ‘Full follow-up for cardiotoxicity was defined as time from the start of induction I chemotherapy to the first documentation of cardiotoxicity. Patients who did not experience cardiotoxicity were censored at relapse, loss to follow-up, or 5 years after the start of treatment; deaths were considered competing events.’ | Type and number of participants:  1022 pediatric patients with acute myeloid leukemia (AML)  ‘All patients enrolled in AAML0531 provided informed consent for use of trial data for research’  Diagnosis:  AML  Age at cancer diagnosis:  N = 207/1022 (20.3%) age 0-1 years  N = 354/1022 (34.6%) age 2-10 years  N = 461/1022 (45.1%) age ≥ 11 years  Gender:  Male N = 508/1022 (49.7%)  Female N = 514/1022 (50.3%)  Patients included in overall and on-protocol analyses: N = 949/1022 (92.9%)  Controls:  Not applicable  Cardiovascular risk factors:  Obesity N = 194/1022 (19.0%), defined as weight ≥95^th^ percentile  Prior cardiotoxic treatment:  Not reported  Prior cardiac dysfunction:  Not reported  Prior cardioprotective interventions:  Not applicable | Anthracyclines:  Daunorubicin 50mg/m^2^/dose or 1.67 mg/kg/dose if body-surface area (BSA) <0.6m^2^, administered as 6-hour intravenous infusion on day 1, 3 and 5 of induction I and II.  No detailed information about treatment modifications given, no cumulative dosage anthracyclines given.  Mitoxantrone:  12mg/m^2^/dose or 0.4mg/kg/dose if BSA <0.6m2 as 1-hour infusion on day 3, 4, 5 and 6 of intensification II.  Radiotherapy involving the heart:  Not reported  *‘Dexrazoxane use was not captured but was likely used in <10% of patients’* | Diagnostic test used for cardiotoxicity assessment:  Echocardiography  Timing of the diagnostic test: Mandatory before start of induction courses I and II and intensification II, before hematopoietic stem-cell transplantation, at the end of protocol therapy and at yearly intervals during off-protocol follow-up.  Outcome definitions:  Definition of cardiotoxicity is based on the National Cancer Institute Common Terminology Criteria for Adverse Events (version 3).  Grade 2 or higher LVSD (left ventricular systolic dysfunction) = resting shortening fraction (SF) <24% or ejection fraction (EF) <50%.  Experienced LVSD = when reported on a CRF (case report form) or submitted echo report.  Outcome assessors blinded: Not reported  Occurrence of acute cardiotoxicity:  Acute cardiotoxicity not clearly separated from early-onset cardiotoxicity. See below.  Occurrence of acute and early-onset cardiotoxicity:  *Dichotomous results*  **Course-specific LVSD identified by AE report** (No.) - **by submitted echocardiogram** (No.) - **total LSVD events (AE report or echocardiogram** (No./patients starting course (%))  Ind I = 13 - 10 - 15/1022 (1.5%)  Ind II = 6 - 5 - 10/951 (1.1%)  Int I = 17 - 12 - 20/851 (2.4%)  Int II = 30 - 20 - 31/626 (5.0%)  Int III = 21 - 17 - 24/513 (4.7%)  HSCT = 5 - 2 - 5/158 (3.2%)  6 months FU = 36 - 30 - 40/951 (4.2%)  12 months FU = 11 - 9 - 14/795 (1.8%)  **Cumulative total of first occurrence of LVSD** (No./ patients starting course (%)).  Ind I = 15/1022 (1.5%)  Ind II = 22/951(2.3%)  Int I = 39/851 (4.6%)  Int II = 64/626 (10.2%)  Int III = 83/513(16.2%)  HSCT = 88/158 (55.7%)  6 months FU = 111/951 (11.7%)  12 months FU = 118/795 (14.8%)  Median time to cardiotoxicity 4.3 months (interquartile range, 3.1 to 5.9).  **Distribution of incident cardiotoxicity by reporting period and grade of initial onset**  - Ind I = N=10/15 (66.7%) grade 2; N=4/15 (26.7%) grade 3; N=1/15 (6.7%) grade 4  - Ind II = N=6/7 (85.7%) grade 2; N=1/7 (14.2%) grade 4  - Int I = N=14/17 (82.4%) grade 2; N=3/17 (17.6%) grade 3  - Int II = N=14/25 (56.0%) grade 2; N=9/25 (36.0%) grade 3; N=1/25 (4.0%) grade 4, N=1/25 (4.0%) grade 5  - Int III = N=11/19 (57.9%) grade 2; N=5/19 (26.3%) grade 3; N=3/19 (15.7%) grade 4  - HSCT = N=2/5 (40.0%) grade 2; N=3/5 (60.0%) grade 3  - 6 months FU = N=11/23 (47.8%) grade 2; N=10/23 (43.5%) grade 3; N=2/23 (8.7%) grade 4  - 12 months FU = N=7/7 (100.0%) grade 2  61% of incident LVSD first documented as grade 2  39% of incident LVSD as grade 3 or higher  Mortality:  After completion of  - Ind I, N = 18/1022 (1.8%)  - Ind II, N = 4/951 (0.4%)  - Int I, N = 2/851 (0.2%)  - Int II, N = 12/626 (1.9%)  - Int III, N = 10/513 (1.9%)  - HSCT, N = 5/158 (3.2%)  Total deaths after completion of therapy  N = 51/1022 (5.0%)  ‘Among patients who completed protocol-planned therapy and experienced LVSD during  the off-protocol period (N = 30), there were 12 deaths, of which 75% occurred postrelapse.’  Risk factors assessed: Yes  Results of multivariate analyses:  ‘Fine and Gray methods were used to compute hazard ratios comparing cardiotoxicity incidence by covariables’  Results for off-protocol follow-up not separated in early-onset or late-onset. Therefore we only used the results of the multivariate analyses for the on-protocol results.  **Age at diagnosis (years)**  - 0-1 years, N = 189/949 (19.9%), hazard ratio (HR) 0.21 (95% confidence interval (95% CI) 0.06 to 0.69), *P* <0.05  - 2-10 years, N = 334/949 (35.2%), reference  - ≥ 11 years, N = 426/949 (44.9%), HR 1.37 (95% CI 0.85 to 2.21)  **Sex**  - Female, N = 470/949 (49.5%), HR 1.30 (0.84 to 2.02)  - Male, N = 479/949 (50.5%), reference  **Race**  - White, N = 713/949 (75.1%), reference  - Black, N = 109/949 (11.5%), HR 2.18 (1.27 to 3.75), *P* <0.05  - Other, N = 127/949 (13.4%), 0.81 (0.38 to 1.71)  **Ethnicity**  - Hispanic or Latino, N = 178/949 (18.8%), HR 0.63 (0.32 to 1.25)  - Not Hispanic or Latino, N = 771/949 (81.2%), reference  **Weight category**  - Underweight, N = 66/949 (7.0%), HR 0.55 (0.17 to 1.78)  - Normal weight, N = 559/949 (58.9%), reference  - Overweight/obese, N = 324/949 (34.1%), HR 1.04 (0.66 to 1.66)  **Cytogenetic risk group**  - Low [t(8;21) or inv(16)], N = 239/949 (25.2%), reference  - Intermediate, N = 676/949 (71.2%), HR 1.03 (0.64 to 1.66)  - High (-5/del5q or -7), N = 34/949 (3.6%), HR 1.04 (0.24 to 4.40)  **Initial WBC count**  - ≤100,000/µL, N = 771/949 (81.2%), reference  - >100,000/µL, N = 178/949 (18.8%), HR 0.61 (0.31 to 1.19)  **Randomized treatment arm**  - Standard, N = 476/949 (50.2%), reference  - Standard plus GMTZ (gemtuzumab ozogamicin), N = 473/949 (49.8%), HR 0.82 (0.53 to 1.27)  **Microbiologically documented bloodstream infection during treatment**  - Yes, N = 683/949 (72.0%), HR 1.90 (0.98 to 3.71)  - No, N = 266/949 (28.0%), reference | Selection bias: Low risk (original cohort well-described, all patients included in AAML0531 protocol)  Attrition bias: High risk (only 54% of reporting periods have documentation of completed echoes, during on-protocol period).  Detection bias: Unclear risk (blinding for echocardiography not mentioned)  Confounding: High risk (some prognostic factors for cardiac function (age and sex) are taken into account, however several factors such as prior cardiac dysfunction, prior cardiotoxic treatment and follow-up not taken into account)  Reporting bias (study group): Not well-defined (Detailed information on treatment modifications were not collected. No cumulative anthracycline dosage).  Reporting bias (follow-up): Well-defined (length of follow-up clearly stated)  Reporting bias (outcome): Well-defined (method of detection and definition of abnormal outcome are provided)  Risk estimation analyses: Well-defined (hazard ratio is provided)  Funding of the trial:  Supported by a National Clinical Trials Network Operations Center Grant  (U10CA180886), National Clinical Trials Network Statistics & Data  Center Grant (U10CA180899), and St Baldrick’s Foundation.  Overlap with other included studies:  Not presumed |

| **What is the frequency of occurrence of acute and early-onset cardiotoxicity, as diagnosed by clinical, echocardiographic and biochemical parameters routinely used in clinical practice, in children with cancer treated with anthracyclines, mitoxantrone and/or radiotherapy involving the heart? What are the risk factors?** | | | | |
| --- | --- | --- | --- | --- |
| *Gupta et al.: Role of ACE inhibitors in anthracycline-induced cardiotoxicity: A randomized, double-blind, placebo-controlled trial. Pediatr Blood Cancer. 2018;65:e27308.* | | | | |
| **Study design; treatment era; follow-up** | **Participants** | **Treatment** | **Diagnostic test; main outcomes** | **Risk of bias assessment** |
| Study design: Randomized, double-blind, placebo-controlled trial, but as only one arm was eligible for this review this study was seen as a prospective cohort study  Treatment era:  August 1, 2014 - September 30, 2016  Follow-up:  6 months (baseline to 6 months after), not further described. | Type and number of participants:  Original cohort N = 98 patients with acute lymphoblastic leukemia / lymphoma with projected cumulative anthracycline dose >200 mg/m2  N = 4 not eligible, N = 2 withdrew consent.  N = 48 in experimental group (enalapril)  N = 44 in placebo group  Information provided in this table is for the patients treated with anthracyclines but no enalapril.  After dropouts (N = 4): N = 40 in placebo group  Diagnosis:  N = 19/40 (47.5%) acute leukemia  N = 21/40 (52.5%) lymphoma  Age at cancer diagnosis:  Mean age 8.77 years ± 2.86 SD  Gender:  Male N = 30/40 (75%)  Female N = 10/40 (25%)  Controls:  Not applicable  Cardiovascular risk factors:  Not reported  Prior cardiotoxic treatment:  None (exclusion criteria mentions ‘previously treated’)  Prior cardiac dysfunction:  None (exclusion criteria mentions ‘preexisting cardiac disease (valvular disease, cardiomyopathy)’)  Prior cardioprotective interventions:  Not applicable | Anthracyclines:  Cumulative anthracycline dosage = 263.64 mg/m^2^ ± 80.90 SD. More detailed information about anthracyclines not mentioned (no infusion duration).  Mitoxantrone:  Not reported  Radiotherapy involving the heart:  Not reported | Diagnostic test used for cardiotoxicity assessment:  Two-dimensional echocardiography and cardiac biomarkers (laboratory tests)  Timing of the diagnostic test:  At baseline and after 6 months.  Outcome definitions:  Cardiotoxicity = a **decrease in left ventricle ejection fraction (LVEF) of ≥ 20%** (from baseline to 6 months).  **CK-MB** normal range 0.0 – 4.3, abnormal >4.3 ng/mL  **cTnI** normal range 0.0 – 0.02, abnormal >0.02 ng/mL  **pro-BNP** normale range 0.0 – 100, abnormal >100 pg/mL  Outcome assessors blinded: Not reported  Occurrence of acute cardiotoxicity: Not reported  Occurrence of early-onset cardiotoxicity:  *Dichotomous results*  N = 37/40 (92.5%) had a <20% decrease in LVEF  N = 3/40 (7.5%) had a **≥ 20% decrease in LVEF**  N = 15/40 (37.5%) had **pro-BNP levels ≥100pg/mL**  *Continuous results*  - **LVEF** baseline (N = 40) mean 64.85 ± 4.94 SD, after 6 months mean 56.15 ± 4.79 SD  - **cTnI** baseline mean 0.01 ± 0.00 SD, after 6 months mean 0.011 ± 0.003 SD  - **pro-BNP** baseline mean 5.00 ± 0.00 SD, after 6 months mean 98.60 ± 54.24 SD  - **CK-MB** baseline mean 1.00 ± 0.00 SD, after 6 months mean 1.21 ± 0.44 SD  Mortality:  Not reported  Risk factors assessed: No  Results of multivariate analyses: Not applicable | Selection bias: Low risk, (92/98 (93.9%) of eligible participants were included)  Attrition bias: Low risk (40/44 (90.1%) were assessed for outcomes)  Detection bias: Unclear risk for echocardiography, blinding of assessors not mentioned.  Low risk for laboratory tests (blinding not mentioned but as blinding is not relevant for outcomes diagnosed by laboratory tests we judged this outcome at low risk of detection bias  Confounding: Not applicable  Reporting bias (study group): Not well-defined, cumulative dosage mentioned but no specifics or infusion duration.  Reporting bias (follow-up): Not well-defined (no mean of follow-up mentioned)  Reporting bias (outcome): Well-defined for LVEF and pro-BNP (method of detection is clear, reference values for abnormal outcomes are clear), not well-defined for CK-MB and cTnI (method of detection and reference values for abnormal outcomes mentioned, but not applied in results)  Risk estimation analyses: Not applicable  Funding of the trial: None reported  Overlap with other included studies:  Not presumed |

| **What is the frequency of occurrence of acute and early-onset cardiotoxicity, as diagnosed by clinical, echocardiographic and biochemical parameters routinely used in clinical practice, in children with cancer treated with anthracyclines, mitoxantrone and/or radiotherapy involving the heart? What are the risk factors?** | | | | |
| --- | --- | --- | --- | --- |
| *Hagag et al.: Protective Role of Silymarin in Early Doxorubicin-induced Cardiac Dysfunction in Children with Acute Lymphoblastic Leukemia. Infectious Disorders - Drug Targets, 2018 (18): pages 1-9* | | | | |
| **Study design; treatment era; follow-up** | **Participants** | **Treatment** | **Diagnostic test; main outcomes** | **Risk of bias assessment** |
| Study design:  Randomized controlled trial (single center), but as only one arm was eligible for this review this study was seen as a prospective cohort study  Treatment era:  April 2015 - December 2017  Follow-up:  6 weeks (from start therapy to 6 weeks after, not further described) | Type and number of participants:  N = 80 children with newly diagnosed acute lymphoblastic leukemia (ALL)  Original cohort unclear.  N = 40 under doxorubicin therapy and silymarin (group I)  N = 40 under doxorubicin therapy and placebo  Information provided in this table is for the patients treated with doxorubicin but no silymarin.  Diagnosis:  ALL  Age at cancer diagnosis:  Mean age 7.75 years ± 3.05 SD (range 3 – 13.5), median age 7.25 years.  Gender:  Male N = 24/40 (60%)  Female N = 16/40 (40%)  Controls:  Not applicable  Cardiovascular risk factors:  Not reported  Prior cardiotoxic treatment:  Not applicable (‘newly diagnosed’)  Prior cardiac dysfunction:  None (exclusion criteria mentions ‘patients with ALL with previous cardiac diseases as rheumatic heart disease’)  Prior cardioprotective interventions:  Not reported | Anthracyclines:  Dose according to protocol, actual received cumulative dose not described.  Doxorubicin 25 mg/m^2^/week IV infusion (days 0, 7, 14, 21, 28, 35)  Infusion duration not mentioned.  Mitoxantrone:  Not applicable  Radiotherapy involving the heart:  Not reported | Diagnostic test used for cardiotoxicity assessment:  Echocardiography, laboratory tests (troponin I).  Timing of the diagnostic test:  Before starting chemotherapy and after 6 weeks of chemotherapy.  Outcome definitions:  Ejection fraction denoted systolic dysfunction if <50%. No other outcome definitions provided.  Outcome assessors blinded: Not reported.  Occurrence of acute and early-onset cardiotoxicity (not separated):  *Dichotomous parameters*  **EF <50%** N = 0/40  *Continuous parameters*  **E/A ratio** (m/second) before treatment mean 1.31 ± 0.16 SD (range 1.1 – 1.6), median 1.3.  **E/A ratio** (m/second) after treatment mean 1.28 ± 0.04 SD (range 1 – 1.3), median 1.3.  **Ejection fraction** (EF, %) before treatment mean 68.25 ± 3.91 SD (range 62 – 74), median 67  **Ejection fraction** (EF, %) after treatment mean 54.9 ± 5.35 SD (range 50 - 69), median 53 (*P* <0.001)  **Fractional shortening** (%) before treatment mean 35.6 ± 1.93 SD (range 32 - 38), median 36  **Fractional shortening** (%) after treatment mean 29.35 ± 1.63 SD (range 27 - 33), median 29 (*P* <0.001)  **Serum Troponin I** (pg/ml) before treatment mean 37.3 ± 8.71 SD (range 28.8 – 45.3), median 35.66.  **Serum Troponin I** (pg/ml) after treatment mean 75.5 ± 7.71 SD (range 67.0 – 85.5), median 76.33 (*P* = 0.027)  Mortality:  Not reported  Risk factors assessed: No  Results of multivariate analyses: Not applicable | Selection bias: Unclear risk (original cohort unclear)  Attrition bias: Low risk for echocardiography (all patients had outcome assessment), low risk for laboratory tests (all patients had outcome assessment).  Detection bias: Unclear risk for echocardiography, blinding of the assessors is not mentioned. Low risk for laboratory tests (blinding not mentioned but as blinding is not relevant for outcomes diagnosed by laboratory tests we judged this outcome at low risk of detection bias)  Confounding: Not applicable  Reporting bias (study group): Not well-defined (no cumulative dosage or our dosage changes mentioned, no infusion duration mentioned)  Reporting bias (follow-up): Not well-defined (no mean of follow up mentioned, however, clear cut-off follow-up (6 weeks), so could be interpretable as well-defined).  Reporting bias (outcome):  Well-defined for EF (method of detection and definition of abnormal outcom provided).  Not well-defined for other outcomes (method of detection is clear, however, no definitions of abnormal outcome given).  Risk estimation analyses: Not applicable  Funding of the trial:  None reported  Overlap with other included studies:  Not presumed |

| **What is the frequency of occurrence of acute and early-onset cardiotoxicity, as diagnosed by clinical, echocardiographic and biochemical parameters routinely used in clinical practice, in children with cancer treated with anthracyclines, mitoxantrone and/or radiotherapy involving the heart? What are the risk factors?** | | | | |
| --- | --- | --- | --- | --- |
| *Hu et al.: Detection of Subclinical Anthracyclines’ Cardiotoxicity in Children with Solid Tumor. Chinese Medical Journal: June 20, 2018 (volume 131), issue 12: pages 1450 – 1456.* | | | | |
| **Study design; treatment era; follow-up** | **Participants** | **Treatment** | **Diagnostic test; main outcomes** | **Risk of bias assessment** |
| Study design:  Single center retrospective cohort study according to authors, as the diagnostic test is only reported at one time point also possible to classify as cross-sectional study.  Treatment era:  January 2015 - December 2016  Follow-up:  Unclear, from start therapy to end of therapy.  ‘Duration of therapy was >1 year and all patients were in complete remission at the time of the study’ | Type and number of participants:  N = 36 asymptomatic children with hepatoblastoma (HB) or rhabdomyosarcoma (RMS)  Original cohort unclear.  Diagnosis:  N = 26/36 (72.2%) HB  N = 10/36 (27.8%) RMS  Age at cancer diagnosis:  Mean age 3.6 years ± 2.2 SD  Gender:  Male N = 24/36 (66.7%)  Female N = 12/36 (33.3%)  Controls:  N = 36 healthy children with no congenital cardiovascular disease, matched based on age and gender  Male N = 20/36 (55.6%)  Female N = 16/36 (44.4%)  Mean age 3.9 years ± 2.2 years.  Cardiovascular risk factors: Not reported  Prior cardiotoxic treatment:  None (exclusion criteria mentions ‘no other cardiotoxic drugs administered’)  Prior cardiac dysfunction: None (exclusion criteria mentions ‘no congenital cardiovascular disease or cardiovascular system metastases’)  Prior cardioprotective interventions:  Not applicable | Anthracyclines:  - HB patients: pirarubicin (25mg/m^2^, day 1-3)  - RMS patients: pirarubicin (25mg/m^2^, day 2 and 9)  Number of courses not specified  Cumulative dosage ranged from 24.8 mg/m^2^ to 772.0 mg/m^2^. Mean accumulative anthracycline dosage = 293.7 mg/m^2^ ± 35.4 SD.  N = 21/36 (58.3%) had cumulative dosage <300mg/m^2^  N = 15/36 (41.7%) had cumulative dosage ≥300mg/m^2^.  Mitoxantrone:  Not reported  Radiotherapy involving the heart: Not reported | Diagnostic test used for cardiotoxicity assessment:  Echocardiograpy (transthoracic).  Timing of the diagnostic test: Echocardiography after the last chemotherapy session.  Outcome definitions: Not reported.  Outcome assessors blinded: Not reported  Occurrence of acute cardiotoxicity: Not reported.  Occurrence of early-onset cardiotoxicity:  N = 0 /36 (0%) had heart failure, mean LVEF within the normal range of 65.7% ± 5.1 SD.  *Continuous parameters*  **Left ventricular ejection fraction** (LVEF, %): Patient group mean 65.7 ± 5.1 SD; Control group mean 66.6 ± 3.4 SD; *P* = 0.52  **Left ventricular global longitudinal strain** (GLS, %): Patient group mean -17.9 ± 2.9 SD; Control group mean -22.2 ± 1.9 SD; *P* <0.01  *Differentiated between anthracycline cumulative dosage*  **LVEF** (%) subgroup <300mg/m^2^ (N=21) mean 65.9 ± 5.5 SD. Subgroup ≥300mg/m^2^ (N=15) mean 66.5 ± 4.6 SD; *P* = 0.83.  **GLS** (%) subgroup <300mg/m^2^ (N=21) mean -18.7 ± 2.7 SD. Subgroup ≥300mg/m^2^ (N=15) mean -16.5 ± 2.1; *P* = 0.04.  Mortality:  None  Risk factors assessed: No  Results of multivariate analyses: Not applicable | Selection bias: Unclear risk (original cohort unclear)  Attrition bias: Low risk (all outcomes assessed in all participants).  Detection bias: Unclear risk, blinding of assessors of echocardiography not mentioned.  Confounding: Not applicable.  Reporting bias (study group): Well-defined, cumulative dosage of anthracyclines mentioned.  Reporting bias (follow-up): Not well-defined (no duration or mean of follow-up given)  Reporting bias (outcome): Not well-defined (method of detection is clearly stated, but no definition of abnormal outcome is given).  Risk estimation analyses: Not applicable  Funding of the trial:  Supported by a grant from Research Backbone Training Fund of Beijing Tongren Hospital (No. 2015‑YJJ‑GGL‑009).  Overlap with other included studies:  Not presumed |

| **What is the frequency of occurrence of acute and early-onset cardiotoxicity, as diagnosed by clinical, echocardiographic and biochemical parameters routinely used in clinical practice, in children with cancer treated with anthracyclines, mitoxantrone and/or radiotherapy involving the heart? What are the risk factors?** | | | | |
| --- | --- | --- | --- | --- |
| *Hu et al.: Cardiotoxicity of anthracycline (ANT) treatment in children with malignant tumors. Pediatric hematology and oncology. 35:2, 111-120* | | | | |
| **Study design; treatment era; follow-up** | **Participants** | **Treatment** | **Diagnostic test; main outcomes** | **Risk of bias assessment** |
| Study design:  Single center cohort study  Treatment era:  January 2011 - December 2015  Follow-up:  Not reported | Type and number of participants:  N = 131 children with malignant tumors who were treated with anthracycline (ANT) chemotherapy.  Original cohort unclear  Diagnosis:  N = 85/131 (64.9%) hepatoblastoma, HB  N = 41/131 (31.3%) rhabdomyosarcoma, RMS  N = 5/131 (3.8%) myeloid sarcoma, MS  Age at cancer diagnosis:  Median age of onset 2 years, range 0.08 – 12 years  Gender:  Male N = 72/131 (55.0%)  Female N = 59/131 (45.0%)  Controls:  Not applicable  Cardiovascular risk factors:  Not reported  Prior cardiotoxic treatment:  None (exclusion criteria mentions ‘target drugs’)  Prior cardiac dysfunction:  None (exclusion criteria mentions ‘congenital heart disease’ and ‘cardiovascular system metastasis’)  Prior cardioprotective interventions:  Not reported | Anthracyclines:  N = 85 HB: pirarubicin 25mg/m^2^/d, day 1-3  N = 41 RMS: pirarubicin 25mg/m^2^/d, days 2 and 9  N = 5 MS: pirarubicin 25mg/m^2^/d, day 1-3  No infusion duration stated. Patients were treated with 3-17 cycles of chemotherapy.  Cumulative anthracycline dosage range was 12 – 697mg/m^2^ (median: 120.5 mg/m^2^, mean: 154.66 mg/m^2^ ± 127.04 mg/m^2^)  Cumulative dosage of ANT in the 2 patients with heart failure was 616.9mg/m^2^ and 511.2mg/m^2^, respectively  Groups divided by cumulative ANT dosage  Group 1, N = 49/131 (37.4%) = <100mg/m^2^  Group 2, N = 51/131 (38.9%) = ≥100 and <200mg/m^2^  Group 3, N = 31/131 (23.7%) = ≥200mg/m^2^  (Of which N = 20/31 ≥200 and <300mg/m^2^ and N = 11/31 ≥300mg/m^2^)  Mitoxantrone:  N = 5 MS: mitoxantrone 8mg/m^2^/d, day 1-5  Radiotherapy involving the heart:  None (exclusion criteria mentions ‘combined chest radiotherapy’) | Diagnostic test used for cardiotoxicity assessment:  Laboratory tests, echocardiography  Timing of the diagnostic test:  Laboratory tests: before chemotherapy and on the 7^th^ day of the first cycle of ANT chemotherapy.  Echocardiography: before treatment and the month when the treatment involving the cumulative dose of ANT was performed. However, only post-treatment values reported.  Outcome definitions:  **NT-proBNP** normal range = 0-62 pg/ml.  **cTnT** normal reference values <0.05 ng/ml  **CK-MB** normal reference range = 0-25 U/L  **Clinical cardiotoxicity** is the occurrence of left ventricular ejection fraction (LVEF) <53% and heart failure (HF) symptoms.  Outcome assessors blinded: Not reported  Occurrence of acute cardiotoxicity:  *Continuous results*  Post-treatment **NT-proBNP** (pg/ml) group 1 mean 142.93 ± 104.43 SD, 95% CI 112.94 – 172.93; group 2 mean 158.27 ± 78.18 SD, 95% CI 136.28 – 180.26; group 3 mean 1725.90 ± 5634.78 SD, 95% CI 340.95 – 3792.75. *P* = 0.022.  Pre- and posttreatment **cTnT** (ng/mL) <0.05 in all groups  Post-treatment **CK-MB** (U/L) group 1 mean 30.06 ± 11.43 SD, 95% CI 26.78 – 33.34; group 2 mean 27.53 ± 8.80, 95% CI 25.05 – 30.00; group 3 mean 25.67 ± 12.31, 95% 21.18 – 29.90. *P* = 0.190  Occurrence of early-onset cardiotoxicity:  *Dichotomous results*  2/131 (1.5%) developed **clinical cardiotoxicity**.  *Continuous results*  **LVEF** (%) group 1 mean 68.88 ± 6.79 SD, 95% CI 66.92 – 70.83; group 2 mean 69.35 ± 2.73 SD, 95 CI 68.58 – 70.12; group 3 mean 65.95 ± 7.94 SD, 95% CI 63.05 – 68.88. *P* = 0.035.  **FS** (%) group 1 mean 37.22 ± 3.56 SD, 95% CI 36.2 – 38.24; group 2 mean 37.37 ± 5.01 SD, 95% CI 35.96 – 38.78; group 3 mean 36.54 ± 4.8 SD, 95% CI 32.08 – 36.11. *P* = 0.005.  Mortality:  Not reported  Risk factors assessed:  Yes  Results of multivariate analyses:  ‘Multiple linear regression set the cumulative dose of ANT as the dependent variable, and included the post-treatment serum NT-proBNP, age and QT-c in the regression equation for the analysis; the result showed F = 11.359 and *P* = 0.000 (<0.001). The standard coefficient of NT-proBNP was 0.423 and *P* = 0; that of age was 0.184 and *P* = 0.021; that of QT-c was 0.191 and *P* = 0.018.’ | Selection bias:  Unclear risk (original cohort not described)  Attrition bias:  Low risk (all included patients had outcomes for both laboratory tests and echocardiography)  Detection bias:  Unclear risk for echocardiography, not stated whether assessors were blinded for echocardiography.  Low risk for laboratory tests (blinding not mentioned but as blinding is not relevant for outcomes diagnosed by laboratory tests we judged this outcome at low risk of detection bias)  Confounding: High risk (risk factors analyzed in multiple linear regression, but only few variables taken into account, for example sex not considered)  Reporting bias (study group): Well-defined (cumulative dosage of anthracyclines mentioned)  Reporting bias (follow-up): Not well-defined (no duration of follow-up mentioned)  Reporting bias (outcome): Well-defined for echocardiography (method of detection and definition of abnormal outcome stated).  Not well-defined for laboratory tests (method of detection and definition of abnormal outcome stated, but results only reported in a continuous fashion).  Risk estimation analyses:  Well-defined (multiple linear regression model provided)  Funding of the trial:  Research Backbone Training Fund of Beijing Tongren Hospital [2015-YJJ-GGL-009]  Overlap with other included studies:  Not presumed |

| **What is the frequency of occurrence of acute and early-onset cardiotoxicity, as diagnosed by clinical, echocardiographic and biochemical parameters routinely used in clinical practice, in children with cancer treated with anthracyclines, mitoxantrone and/or radiotherapy involving the heart? What are the risk factors?** | | | | |
| --- | --- | --- | --- | --- |
| *Ishii et al.: Sequential Evaluation of Left Ventricular Myocardial Performance in Children After Anthracycline Therapy. The American Journal of Cariology, 2000, volume 86: pages 1279 - 1281* | | | | |
| **Study design; treatment era; follow-up** | **Participants** | **Treatment** | **Diagnostic test; main outcomes** | **Risk of bias assessment** |
| Study design:  Single center prospective cohort study  Treatment era:  Not reported  Follow-up:  Not reported | Type and number of participants:  N = 65 patients receiving anthracycline antibiotic therapy for malignant neoplasms.  Original cohort unclear  Diagnosis:  Not reported  Age at cancer diagnosis:  Not reported  Gender:  Not reported  Controls:  N = 81 age-matched healthy children (mean age 7.9 years ± 4.3 SD)  ‘There were no significant differences in age, heart rate, body weight, height and blood pressure.’  Cardiovascular risk factors: Not reported  Prior cardiotoxic treatment:  ‘None of these patients had received prior mediastinal radiation’  Prior cardiac dysfunction:  Not reported  Prior cardioprotective interventions:  Not reported | Anthracyclines:  Group A, N = 35/65 (53.8%) received low dose (<200mg/m^2^)  Group B, N = 30/65 (46.2%) received moderate to high dose (≥200mg/m^2^)  Infusion duration or other specifics on anthracyclines not mentioned.  Mitoxantrone:  Not reported  Radiotherapy involving the heart:  Not reported | Diagnostic test used for cardiotoxicity assessment:  Echocardiography  Timing of the diagnostic test:  Before every administration of anthracycline antibiotics (not clearly indicated at what time point reported Tei index was measured).  Outcome definitions:  *Dichotomous parameters:* **Tei index**, range of normal values in the left ventricle = Mean 0.33 ±0.02 SD (based on control group).  Outcome assessors blinded: Partly (primary assessment not described, but interobserver variance tested by 2 independent observers with no knowledge of previous outcomes in 20 randomly selected patients)  Occurrence of acute and early-onset cardiotoxicity (not separated): *Continuous parameters*  **Tei index** in group A mean 0.34 ± 0.09 SD and group B mean 0.45 ± 0.06 SD. Significant difference in Tei index between groups A and B (*P* <0.05) and between controls and group B (*P* <0.05).  **Left ventricular ejection fraction** and **fractional shortening** showed no significant differences between the groups (not quantified).  Group B:  - N = 9/30 (30.0%) abnormal Tei index during low-dose (<200mg/m^2^) period, all had normal fractional shortening  - N = 25/30 (83.3%) abnormal Tei index during moderate dose (≥200mg/m^2^ and <400mg/m^2^) period, all had normal fractional shortening  - N = 12/12 (100%) who received high dose (≥400mg/m^2^) abnormal Tei index (mean 0.48 ± 0.06), 5/12 (41.7%) had abnormal fractional shortening  Mortality:  Not reported  Risk factors assessed: No  Results of multivariate analyses: Not applicable | Selection bias:  Unclear risk (original cohort unclear)  Attrition bias: Unclear risk (not clear how many echocardiograms were made, if any patients were lost to follow-up).  Detection bias: Unclear risk (blinding of primary assessment not described, but interobserver variance tested by 2 independent observers with no knowledge of previous outcomes in 20 randomly selected patients, which was low).  Confounding: Not applicable  Reporting bias (study group): Not well-defined, no specifics on anthracycline therapy given, no infusion duration, no mean of cumulative dosage)  Reporting bias (follow-up): Not well-defined, no length of follow-up given  Reporting bias (outcome): Not-well defined for left ventricular ejection fraction and fractional shortening (no definition of abnormal outcome given), well-defined for Tei index (method of detection and definition of abnormal outcome given).  Risk estimation analyses: Not applicable  Funding of the trial:  Not reported  Overlap with other included studies:  Not presumed |

| **What is the frequency of occurrence of acute and early-onset cardiotoxicity, as diagnosed by clinical, echocardiographic and biochemical parameters routinely used in clinical practice, in children with cancer treated with anthracyclines, mitoxantrone and/or radiotherapy involving the heart? What are the risk factors?** | | | | |
| --- | --- | --- | --- | --- |
| *Kang et al.: Cardioprotective effect of early dexrazoxane use in anthracycline treated patients. Journal of Chemotherapy, 2012; volume 24 (No.5): 292 - 296* | | | | |
| **Study design; treatment era; follow-up** | **Participants** | **Treatment** | **Diagnostic test; main outcomes** | **Risk of bias assessment** |
| Study design:  Retrospective cohort study (single center)  Treatment era:  January 1997 to May 2005  Follow-up:  Range of anthracycline treatment was ranged from 4 to 20 months, follow-up not further described | Type and number of participants:  N = 258 newly diagnosed pediatric cancer patients, with no prior treatment with anthracycline, who presented at a tertiary teaching hospital in Korea.  ‘Patients without exact echocardiography records or who had expired immediately after the diagnosis were excluded’, not quantified.  Group A, N = 123/258 (47.7%) received anthracycline alone without dexrazoxane  Group LA+D, N = 85/258 (32.9%) received anthracyclines and dexrazoxane when the cumulative dose of anthracycline was <100mg/m^2^  Group HA+D, N = 50/258 (19.4%) received anthracyclines and dexrazoxane when the cumulative dose of anthracycline was >100mg/m^2^  Information provided in this table is for the patients treated with doxorubicin but no dexrazoxane.  Diagnosis:  N = 52/123 (42.3%) leukemia  N = 20/123 (16.3%) lymphoma  N = 6/123 (4.9%) Ewing’s sarcoma  N = 4/123 (3.3%) osteosarcoma  N = 15/123 (12.2%) neuroblastoma  N = 14/123 (11.4%) hepatoblastoma  N = 12/123 (9.8%) other tumors  Age at cancer diagnosis:  Median age 6 years (range 0.2 – 15.0)  Gender:  Male N = 68/123 (55.3%)  Female N = 55/123 (44.7%)  Controls:  Not applicable  Cardiovascular risk factors:  Not reported  Prior cardiotoxic treatment:  None (‘no prior treatment with anthracycline (doxorubicin, daunorubicin, idarubicin, or mitoxantrone)’)  Prior cardiac dysfunction:  None (‘There were no baseline cases of congestive heart failure or other symptomatic cardiac diseases’)  Prior cardioprotective interventions:  Not reported | Anthracyclines:  Anthracycline treatment according to the type of disease being treated; no further description of how many patients got which cumulative dosage; no infusion duration mentioned  Mitoxantrone:  Not reported  Radiotherapy involving the heart:  Not reported | Diagnostic test used for cardiotoxicity assessment:  Echocardiography  Timing of the diagnostic test:  At baseline before anthracycline treatment and serially when the cumulative dose of anthracycline administered was 100, 200, 300, 400 and 500mg/m^2^.  Outcome definitions:  **Subclinical cardiotoxicity** = abnormal systolic function (greater than 15% reduction of left ventricular ejection fraction (LVEF))  **Dose-limiting cardiotoxicity** = a reduction in LVEF to less than 45% or evidence of clinical congestive heart failure (CHF)  Outcome assessors blinded:  Not reported  Occurrence of acute and early-onset cardiotoxicity (not separated): *Dichotomous results*  N = 9/123 (7.3%) developed **dose-limiting cardiotoxicity**  *Continuous results*  **LVEF** at baseline (N = 123), mean 72.7 ± 5.9 SD  **LVEF** at 100mg/m^2^ cumulative dose (N = 69), mean 69.3 ± 7.8 SD  **LVEF** at 200mg/m^2^ cumulative dose (N = 82), mean 67.7 ± 5.7 SD  **LVEF** at 300mg/m^2^ cumulative dose (N = 55), mean 65.8 ± 8.5 SD  **LVEF** at 400mg/m^2^ cumulative dose (N = 23), mean 63.7 ± 8.6 SD  **LVEF** at 500mg/m^2^ cumulative dose (N = 4), mean 63.0 ± 10.6  **SF** at baseline (N = 123), mean 41.0 ± 5.2 SD  **SF** at 100mg/m^2^ cumulative dose (N = 71), mean 38.6 ± 6.2 SD  **SF** at 200mg/m^2^ cumulative dose (N = 82), mean 37.2 ± 4.8 SD  **SF** at 300mg/m^2^ cumulative dose (N = 54), mean 35.2 ± 6.0 SD  **SF** at 400mg/m^2^ cumulative dose (N = 22), mean 34.2 ± 6.5 SD  **SF** at 500mg/m^2^ cumulative dose (N = 4), mean 33.5 ± 8.0 SD  Mortality: Not reported  Risk factors assessed: No (multivariate analysis performed, but patients who did and did not receive dexrazoxane not separated)  Results of multivariate analyses: Not applicable | Selection bias: Unclear risk (original cohort unclear)  Attrition bias: High risk (for echocardiography all outcomes after baseline are assessed for <90% of the study group, highest is 82/123 (66.7%)  Detection bias: Unclear risk, blinding of assessors of echocardiography not mentioned  Confounding: Not applicable  Reporting bias (study group): Not-well defined, mean cumulative dosage anthracyclines not mentioned, cumulative dosage groups not separately discussed  Reporting bias (follow-up): Not well-defined (no length of follow-up mentioned)  Reporting bias (outcome): Well-defined (method of detection and definition of abnormal outcome well provided)  Risk estimation analyses: Not applicable  Funding of the trial:  Supported by the National Research Foundation of Korea (NRF) grant funded by the Korea government (MEST) (No. 2012.0000185)  Overlap with other included studies:  Not presumed |

| **What is the frequency of occurrence of acute and early-onset cardiotoxicity, as diagnosed by clinical, echocardiographic and biochemical parameters routinely used in clinical practice, in children with cancer treated with anthracyclines, mitoxantrone and/or radiotherapy involving the heart? What are the risk factors?** | | | | |
| --- | --- | --- | --- | --- |
| *Katzenstein et al.: Doxorubicin in combination with cisplatin, 5-flourouracil, and vincristine is feasible and effective in unresectable hepatoblastoma: A Children’s Oncology Group study. Cancer, 2022; 128: 1057 – 1065.* | | | | |
| **Study design; treatment era; follow-up** | **Participants** | **Treatment** | **Diagnostic test; main outcomes** | **Risk of bias assessment** |
| Study design:  Non-randomized, phase 3 COG trial.  Treatment era:  Patients enrolled from September 14, 2009 to March 12, 2012. Latest outcomes from February 10, 2020.  Follow-up:  Median potential follow-up time for EFS: 97 months; 25^th^ and 75^th^ percentiles were 86 and 102 months. Follow-up for cardiotoxicity not specified. | Type and number of participants:  N = 105 patients with newly diagnosed, previously untreated, unresectable, nonmetastatic, Evans stage III hepatoblastoma (HB) or resected, stage I or II HB with small cell undifferentiated (SCU) histology, stratified as intermediate risk.  N = 3 excluded (organ function requirements, disease type, stage/extent of disease)  **N = 102 patients assigned to C5VD chemotherapy and analyzed for feasibility and toxicity**  N = 32 patients removed from protocol early but remained on study  N = 70 patients completed treatment on protocol  Diagnosis:  Hepatoblastoma  N = 6/102 (5.9%) Stage I  N = 3/102 (2.9%) Stage II  N = 93/102 (91.2%) Stage III  Age at cancer diagnosis:  Median age at enrollment: 16 months (range 0-189 months)  Gender:  Male N = 59/102 (57.8%)  Female N = 43/102 (42.2%)  Controls:  Not applicable  Cardiovascular risk factors:  Not reported  Prior cardiotoxic treatment:  None (‘Eligible patients … had not received previous chemotherapy or other tumor-directed therapy’)  Prior cardiac dysfunction:  None (‘Eligible patients … had … normal bone marrow, kidney, liver, and cardiac function’)  Prior cardioprotective interventions:  None | Anthracyclines:  Dose according to protocol, actual received cumulative dose not described (but ‘mean percentage of target dose delivered during cycles 1 to 4 were 95% (95% CI, 93-97%) for doxorubicin’)  Doxorubicin 30mg/m^2^ per dose or 1mg/kg per dose for <10kg, intravenously over 15 minutes on days 1 and 2, no cumulative dosage provided.  Mitoxantrone:  None  Radiotherapy involving the heart:  Not reported  **Dexrazoxane (300mg/m^2^ per dose or 10mg/kg per dose for patients <10kg) by intravenous push was given before doxorubicin during the last 2 cycles of C5VD when the cumulative dose was >300mg/m^2^; therefore we excluded the results after the first 4 cycles** | Diagnostic test used for cardiotoxicity assessment:  Baseline physical examinations, organ function, imaging studies, not further specified for cardiotoxicity  Timing of the diagnostic test: Before initiation of therapy and after 2 and 4 cycles of C5VD  Outcome definitions:  Toxicities were graded according to the National Cancer Institute’s Common Toxicity Criteria guidelines (v3/4).  Toxicities of grade 3 or higher were tabulate for each reporting period.  Outcome assessors blinded:  Not reported  Occurrence of acute cardiotoxicity:  Not separated from early-onset cardiotoxicity  Occurrence of early-onset cardiotoxicity:  Toxicities (grades 3-5)  - N=1/102, 1.0% Left ventricular systolic dysfunction in cycle 1-2  - N = 1/102, 1.0% Cardiac arrest in cycle 1-2 and N = 1/93, 1,1% in cycle 3-4  - N = 1/102, 1.0% Right ventricular dysfunction in cycle 1-2  - N = 1/102, 1.0% Ventricular tachycardia in cycle 1-2  Mortality:  87% survival 12 months from enrollment, EFS 86% 12 months from enrollment  Risk factors assessed: No  Results of multivariate analyses: Not applicable | Selection bias:  Low risk (102/105, 97.1% patients of original cohort included)  Attrition bias: Unclear risk (not described how many patients were completely assessed for cardiotoxicity criteria, i.e. had an echocardiography etc.)  Detection bias: Unclear risk (blinding of assessors for cardiotoxicity not mentioned)  Confounding: Not applicable  Reporting bias (study group): Not well-defined (infusion duration and mean percentage of target dose delivered reported, but no cumulative dosage of anthracyclines provided)  Reporting bias (follow-up): Not well-defined (no length of follow-up provided for cardiotoxicity)  Reporting bias (outcome): Well-defined (method of detection and definition of abnormal outcome provided)  Risk estimation analyses:  Not applicable  Funding of the trial:  Supported by Imaging and Radiation Oncology Core Rhode Island (formerly Quality Assurance Review Center) grant U10 CA 29511, National Clinical Trials Network Operations Center grant U10CA180886, and Statistics and Data Center grant U10CA180899 from the National Cancer Institute of the National Institutes of Healy and by St. Baldrick’s Foundation.  Overlap with other included studies:  Not presumed |

| **What is the frequency of occurrence of acute and early-onset cardiotoxicity, as diagnosed by clinical, echocardiographic and biochemical parameters routinely used in clinical practice, in children with cancer treated with anthracyclines, mitoxantrone and/or radiotherapy involving the heart? What are the risk factors?** | | | | |
| --- | --- | --- | --- | --- |
| *Khairat 2019: Right ventricular 2D speckle-tracking echocardiography in children with osteosarcoma under chemotherapy. The Egyptian Heart Journal, 2019; 71:23* | | | | |
| **Study design; treatment era; follow-up** | **Participants** | **Treatment** | **Diagnostic test; main outcomes** | **Risk of bias assessment** |
| Study design:  Multi-center prospective cohort study  Treatment era:  March 2017 – January 2019  Follow-up:  From baseline to 3 months after ending of chemotherapy, not further specified | Type and number of participants:  N = 100 patients with osteosarcoma in childhood (group 1)  Original cohort unclear.  Diagnosis:  N = 100 /100 (100%) osteosarcoma in the long bone  Age at cancer diagnosis:  Mean age 11.85 years ± 2.09 years (range 9-13 years)  Gender:  Male N = 50/100 (50%)  Female N = 50/100 (50%)  Controls:  N = 100 ages matched healthy control persons (group 2), mean age 12.13 ± 1.87 years  Male N = 52/100 (52%)  Female N = 48/100 (48%)  Cardiovascular risk factors:  Not reported  Prior cardiotoxic treatment:  Not reported  Prior cardiac dysfunction:  None (inclusion criteria mentions ‘normal LV function at baseline’, exclusion criteria mentions ‘valvular heart disease, congenital heart disease, impaired LV function, or impaired LV GLS’  Prior cardioprotective interventions:  Not reported | Anthracyclines:  Doxorubicin intravenous infusion 36.5 mg/m^2^ in week 1, 6, 12, 17, 22 and 26. Anthracycline cumulative dosage = 450mg/m^2^, not further specified, no mean given. No infusion duration given.  Mitoxantrone:  None  Radiotherapy involving the heart:  Not reported | Diagnostic test used for cardiotoxicity assessment:  Echocardiography  Timing of the diagnostic test:  At baseline, 10^th^ week, 20^th^ week, 29^th^ week and 3 months after completion of chemotherapy.  Outcome definitions:  Not reported  Outcome assessors blinded:  Not reported  Occurrence of acute and early-onset cardiotoxicity (not separated):  *At baseline*  Group 1 N = 100, **LVEF** (%) mean 67.7 ± 3.62; **LVGLS** (%) mean -23.77 ± 0.93; **RVGLS** (%) mean -24.75 ± 0.53  Group 2 N = 100, **LVEF** (%) mean 68.2 ± 3.52, *P* = 0.353; **LVGLS** (%) mean -23.83 ± 0.91, *P* = 0.670; **RVGLS** (%) mean -24.64 ± 0.35, *P* = 0.110  *At 10^th^ week of follow-up*  Group 1A: N = 93/100 (93%) patients with **normal** **RVGLS** (RV strain)  Group 1B: N = 7/100 (7%) patients with **decreased RVGLS**  - 1A: **LVEF** (%) mean 66.65 ± 4.03 SD. 1B: LVEF (%) 66.71 ± 1.60 SD. *P* = 0.964  - 1A: **LVGLS** (%) mean -23.93 ± 0.81 SD. 1B: -23.65 ± 1.15 SD. *P* = 0.964.  *At 20^th^ week of follow-up*  Group 1A: N = 90/100 (90%) patients with **normal** **RVGLS** (RV strain)  Group 1B: N = 10/100 (10%) patients with **decreased RVGLS**  - 1A: **LVEF** (%) mean 64.13 ± 7.51 SD. 1B: LVEF (%) 66.50 ± 1.58 SD. *P* = 0.325  - 1A: **LVGLS** (%) mean -23.88 ± 0.86 SD. 1B: -23.60 ± 1.22 SD. *P* = 0.340  *At 29^th^ week of follow-up*  Group 1A: N = 88/100 (88%) patients with **normal** **RVGLS** (RV strain)  Group 1B: N = 12/100 (12%) patients with **decreased** **RVGLS**  - 1A: **LVEF** (%) mean 64.00 ± 7.55 SD. 1B: LVEF (%) 65.75 ± 1.35 SD. *P* = 0.427  - 1A: **LVGLS** (%) mean -23.85 ± 0.89 SD. 1B: -23.43 ± 1.13 SD. *P* = 0.142  *After 3 months of follow-up after therapy*  Group 1A: N = 96/100 (96%) patients with **normal** **RVGLS** (RV strain)  Group 1B: N = 4/100 (4%) patients with **decreased RVGLS**  - 1A: **LVEF** (%) mean 64.31 ± 7.31 SD. 1B: LVEF (%) 65.75 ± 1.50 SD. *P* = 0.697  - 1A: **LVGLS** (%) mean -23.84 ± 0.88 SD. 1B: -23.40 ± 1.61 SD. *P* = 0.339  Mortality:  Not reported  Risk factors assessed:  No  Results of multivariate analyses:  Not applicable | Selection bias: Unclear risk (original cohort unclear)  Attrition bias: Low risk (all included patients were assessed for all outcomes)  Detection bias: Unclear risk (blinding not mentioned)  Confounding: Not applicable  Reporting bias (study group): Not well-defined (mean of cumulative dosage not mentioned, not specified if there were any changes in medication protocol)  Reporting bias (follow-up): Well-defined (length of follow-up given)  Reporting bias (outcome): Not well-defined (method of detection is clear but definition of abnormal outcome not provided)  Risk estimation analyses:  Not applicable  Funding of the trial:  No financial support or scholarship  Overlap with other included studies:  Not presumed |

| **What is the frequency of occurrence of acute and early-onset cardiotoxicity, as diagnosed by clinical, echocardiographic and biochemical parameters routinely used in clinical practice, in children with cancer treated with anthracyclines, mitoxantrone and/or radiotherapy involving the heart? What are the risk factors?** | | | | |
| --- | --- | --- | --- | --- |
| *Kremer et al.: Troponin T in the first 24 hours after the administration of chemotherapy and the detection of myocardial damage in children, 2002, European Journal of Cancer 38: 686-689* | | | | |
| **Study design; treatment era; follow-up** | **Participants** | **Treatment** | **Diagnostic test; main outcomes** | **Risk of bias assessment** |
| Study design:  Prospective cohort study  Treatment era:  December 1998 – May 2000  Follow-up:  From before start of therapy to end of therapy, not further specified | Type and number of participants:  N = 38 children with various kinds of malignancies.  Original cohort unclear.  Diagnosis:  N = 16/38 (42.1%) solid tumor  N = 22/38 (57.9%) leukemia or lymphoma  Age at cancer diagnosis:  Mean age 9.9 years ± 4.7 SD  Gender:  Not specified  Controls:  Not applicable  Cardiovascular risk factors:  Not reported  Prior cardiotoxic treatment:  Not reported  Prior cardiac dysfunction:  Not reported  Prior cardioprotective interventions:  Not reported | Anthracyclines:  N = 20/38 (52.6%) doxorubicin  N = 4/38 (10.5%) daunorubicin  N = 9/38 (23.7%) epirubicin  No specifics on infusion duration given.  Mean cumulative dose at sampling 172 mg/m^2^ ± 112.3 SD  Mean cumulative dose at end of treatment 255 mg/m^2^ ± 118.9 SD  Mitoxantrone:  N = 5/38 (13.2%)  Mean cumulative dose at sampling 67.5 mg/m^2^ ± 26.4 SD  Mean cumulative dose at end of treatment 106 mg/m^2^ ± 13.7 SD  Radiotherapy involving the heart:  Not reported | Diagnostic test used for cardiotoxicity assessment:  Laboratory test cardiac troponin T (cTnT) and echocardiography,  Timing of the diagnostic test:  cTnT: prior, 4-6 hours after and 24 hours after administration of chemotherapy  Echocardiography: before, during and after last cycle of chemotherapy  Outcome definitions:  **Abnormal cTnT** defined as > 0.010 ng/ml  **Left ventricular dysfunction** (LV dysfunction) defined as a shortening fraction (SF) below 30% or a decline of 15% or more from baseline SF  **Cardiotoxic chemotherapy-induced clinical heart failure** defined as heart failure not due to factors other than cardiotoxic chemotherapy.  Outcome assessors blinded: Yes (assessors for both laboratory tests and echocardiography)  Occurrence of acute cardiotoxicity:  N = 3/38 (8%) patients had elevated levels of cTnT (0.018 – 0.040 ng/ml) within 24 hours of therapy.  This was in 6/163 (4%) of collected samples.  Occurrence of early-onset cardiotoxicity:  *Dichotomous outcomes:*  N = 7/32 (21.9%) developed LV dysfunction at the end of therapy (6 patients lost to follow-up/ had no echocardiogram at end of therapy)  N = 1/7 (14.3%) had clinical heart failure  N = 1/7 (14.3%) had elevated cTnT level  *Continuous outcomes:*  **SF** (%) at start of treatment mean = 40.5 ± 3.7 SD  **SF** (%) at end of treatment mean = 36.4 ± 4.6 SD  Mortality:  N = 1/38 (2.6%) died because of tumor progression (2 months after end of therapy)  Risk factors assessed: No  Results of multivariate analyses: Not applicable | Selection bias: Unclear risk (original cohort unclear)  Attrition bias:  Low risk for laboratory tests (total of 163 blood samples during 58 treatment cycles (multiplied by 3 time points per cycle equals 174) from 38 patients, 163/174 (93.7%))  High risk for echocardiography (only 32 /38 (84.2%) had echocardiography at the end of treatment (2 did not finish treatment, 2 left the county, 2 could not obtain good echo window)).  Detection bias: Low risk (technician who performed the array was blinded the both clinical and echocardiographic results and technician who performed echocardiography was unaware of cumulative dose of chemotherapy and cTnT levels)  Confounding: Not applicable  Reporting bias (study group): Not-well defined (no infusion duration given)  Reporting bias (follow-up): Not well-defined (no length of follow-up specified)  Reporting bias (outcome): Well defined (method of detection and definition of abnormal outcome provided)  Risk estimation analyses: Not applicable  Funding of the trial:  None reported  Overlap with other included studies:  Possible overlap with Van Dalen 2006, but different outcomes reported |
| **What is the frequency of occurrence of acute and early-onset cardiotoxicity, as diagnosed by clinical, echocardiographic and biochemical parameters routinely used in clinical practice, in children with cancer treated with anthracyclines, mitoxantrone and/or radiotherapy involving the heart? What are the risk factors?** | | | | |
| *Krischke et al.: Pharmacokinetic and pharmacodynamic study of doxorubicin in children with cancer: results of a “European Pediatric*  *Oncology Off‑patents Medicines Consortium” trial. Cancer Chemotherapy Pharmacology, 2016; 78: 1175-1184* | | | | |
| **Study design; treatment era; follow-up** | **Participants** | **Treatment** | **Diagnostic test; main outcomes** | **Risk of bias assessment** |
| Study design:  Multinational pharmacokinetic study (cohort study)  Treatment era:  April 2010 - October 2012  Follow-up:  From baseline (before first doxorubicin administration) up to 2-4 weeks after 2^nd^ dosage, not further specified | Type and number of participants:  N = 110 patients with solid tumors or leukemia ‘consented to participate in the trial’  N = 9/110 excluded ‘before any baseline data were recorded and plasma samples taken’, (N = 2/9 not meeting inclusion criteria, N = 4/9 declined to participate, N = 3/9 other reasons)  Original cohort unknown.  Study group N = 101  N = 98/101 (97.0%) contributed to biomarker analysis.  N = 7/101 (6.9%) dropped out after first sample period  (N = 4/7 relapse/disease progression, N = 1/7 problems with blood sampling, N = 2/7 withdrawal of consent)  Diagnosis:  N = 12/101 (11.9%) Wilms tumor  N = 9/101 (8.9%) neuroblastoma  N = 27/101 (26.7%) Ewing sarcoma  N = 16/101 (15.8%) soft tissue sarcoma  N = 30/101 (29.7%) acute lymphoblastic leukemia  N = 7/101 (6.9%) other  Age at cancer diagnosis:  Median age 5.3 years (range 0.2 – 17.7)  Gender:  Male N = 50/101 (49.5%)  Female N = 51/101 (50.5%)  Controls:  Not applicable  Cardiovascular risk factors:  Not reported  Prior cardiotoxic treatment:  Not reported  Prior cardiac dysfunction:  Not reported  Prior cardioprotective interventions:  Not reported | Anthracyclines:  Doxorubicin intravenous according to treatment protocol for their tumor entity.  Doxorubicin median dose 28.7 mg/m^2^ (range 10.4 – 57.7).  Doxorubicin infusion duration median 3.88 hours (range 0.25 – 24.02)  Mitoxantrone:  Not reported  Radiotherapy involving the heart:  Not reported | Diagnostic test used for cardiotoxicity assessment:  Laboratory tests: cardiac troponin T (cTnT) and I (cTnI) and natriuretic peptides NT-proANP (A-type natriuretic peptide), NT-proBNP (B-type natriuretic peptide) and BNP.  As biomarker levels were only included in univariate analyses, and absolute values were not reported, we omitted them from reported outcomes.  Echocardiography.  Timing of the diagnostic test:  *Echocardiography:* before and after the two investigated doxorubicin administrations (‘after’ defined as before next protocol block containing doxorubicin, but no later than 28 days after doxorubicin administration).  Outcome definitions:  Echocardiography: one left ventricular shortening fraction (LVSF) value below 28% / one or more LVSF value ≤ 30%  Outcome assessors blinded: Not reported for both echocardiography and laboratory tests.  Occurrence of acute and early-onset cardiotoxicity (not separated): *Dichotomous results:*  N= 6/101 (5.9%) had a **LVSF value below 28%**  N = 20/101 (19.8%) had **one or more LVSF values ≤ 30%**  *Continous results:*  **LVSF** mean at echo 1.1 = 39% ± 8.1 SD and echo 2.2 mean 37.6% ± 6.6 SD.  Mortality: Not reported  Risk factors assessed: No  Results of multivariate analyses: Not applicable | Selection bias: Unclear risk (101/110 (91.8%) of ‘registered patients’ included, but unclear from which cohort registered patients were included)  Attrition bias: Unclear risk for echocardiography (not reported whether outcome was assessed in all patients).  Detection bias: Unclear risk for echocardiography (blinding of assessors not mentioned).  Confounding: Not applicable  Reporting bias (study group): Well-defined (cumulative dosage of anthracyclines and infusion duration specified)  Reporting bias (follow-up): Not-well defined (no mean of follow-up given)  Reporting bias (outcome): Well-defined for echocardiography (method of detection and definition of abnormal outcome provided)  Risk estimation analyses: Not applicable  Funding of the trial:  Sponsorship for the EPOC-MS-001-Doxo-Trial (EudraCTNr:  2009-011454-17, ClinicalTrials.gov Identifier:  NCT01095926) was provided by the University Hospital Münster, Germany  Overlap with other included studies:  Not presumed |

| **What is the frequency of occurrence of acute and early-onset cardiotoxicity, as diagnosed by clinical, echocardiographic and biochemical parameters routinely used in clinical practice, in children with cancer treated with anthracyclines, mitoxantrone and/or radiotherapy involving the heart? What are the risk factors?** | | | | |
| --- | --- | --- | --- | --- |
| *Linares Ballesteros et al.: Early-onset Cardiotoxicity assessment related to anthracycline in children with leukemia. A Prospective Study. Calomb Medica, 2021; 52 (1).* | | | | |
| **Study design; treatment era; follow-up** | **Participants** | **Treatment** | **Diagnostic test; main outcomes** | **Risk of bias assessment** |
| Study design:  Prospective descriptive study  Treatment era:  October 1, 2017 and March 31, 2019  Follow-up:  From start of therapy to end of therapy, not further specified | Type and number of participants:  N = 113 Patients >1y and <18 years diagnosed with acute lymphoblastic leukemia (ALL) and acute myeloid leukemia (AML)  N = 1 patient with AML excluded because of not having data from second evaluation)  Diagnosis:  N = 94/112 (83.9%) ALL  - N=38/112 (33.9%) high risk (HR)  - N=42/112 (37.5%) intermediate risk (IR)  - N=14/112 (12.5%) standard risk (SR)  N = 18/112 (16.1%) AML  Age at cancer diagnosis:  Median age 6.35 years (range 1.0 – 17.7)  Gender:  Male N = 67/112 (59.8%)  Female N = 45/112 (40.2%)  Controls:  Not reported  Cardiovascular risk factors:  Not reported  Prior cardiotoxic treatment:  None (inclusion criteria mentions ‘new diagnosis of acute lymphoblastic leukemia and acute myeloid leukemia’)  Prior cardiac dysfunction:  None (exclusion criteria mentions ‘Patients with a diagnosis of heart disease before the start of cancer treatment’)  Prior cardioprotective interventions:  Not reported | Anthracyclines:  Dose according to protocol, actual received cumulative dose not described.  N=14/112 (12.5%) ALL SR: 170 mg/m^2^  N=42/112 (37.5%) ALL IR: 220 mg/m^2^  N=38/112 (33.9%) ALL HR: 270 mg/m^2^  N = 18/112 (16.1%) AML: 298 mg/m^2^  Infusion duration not reported.  Cumulative doses of anthracyclines were converted to equivalent doses of doxorubicin: 1mg of daunorubicine is equivalent to 0.833mg of doxorubicin  Mitoxantrone:  Not reported  Radiotherapy involving the heart:  Not reported | Diagnostic test used for cardiotoxicity assessment:  Echocardiogram and biomarkers in serum  Timing of the diagnostic test:  1) Before starting treatment (baseline)  2) End of induction (ALL) / end of 1^st^ cycle 7x3 (AML)  3) End of early intensification (ALL SR/IR) / end of high-risk 2 block (ALL HR) / end of second cycle 7x3 (AML)  4) End of late reinduction (ALL) / end of therapy (AML)  Outcome definitions:  Acute cardiac toxicity occurs in the first week after exposure to the neoplastic agent.  Early-onset cardio toxicity occurs between one week and up to a year after the end of antineoplastic treatment.  *Cardiac dysfunction:* Decrease in left ventricle’s systolic performance by estimating its ejection fraction below 53%.  *Serum biomarkers, abnormal when:*  - Brain natriuretic peptide >100 pg/mL  - Troponin I (TnI) >0.05 ng/L  - Troponin T (TnT) >40 ng/L  *Echocardiography*  LVEF (T) = Teicholz method  LVEF (S) = Simpson method  Outcome assessors blinded:  Not reported  Occurrence of acute cardiotoxicity:  N = 0/112 (0%) had acute heart disease  Occurrence of early-onset cardiotoxicity:  *Dichotomous results*  N = 20/112 (17.9%) developed early-onset of cardiac dysfunction  N = 26/112 (23.2%) had elevated BNP  N = 0/112 (0.0%) had elevated troponins  *Continuous results*  **ALL High risk:**  - At diagnosis (N=38) LVEF (T) median 66.0 (range 48-77); LVEF (S) median 63.5 (range 46-70); FS median 36.0 (range 23-46); GLS median -22.0 (range -30 - -18)  - At end of induction (N=38) LVEF (T) median 65.5 (range 37-73); LVEF (S) median 54.0 (range 42-73); FS median 35.0 (range 17-41); GLS median -16.6 (range -22 - -15)  - At end of consolidation (N=30) LVEF (T) median 70.5 (range 65-76); LVEF (S) median 62.5 (range 60-65); FS median 39.0 (range 34-44); GLS median -22.5 (range -23 - -22)  - At end of reinduction (N=17) LVEF (T) median 64.5 (range 50-75); LVEF (S) median 64.7 (range 53-67); FS median 34.5 (range 25-43); GLS median -24.2 (-26.3 - - 18.2)  **ALL Intermediate risk**  - At diagnosis (N=42) LVEF (T) median 68.5 (range 54-78); LVEF (S) median 62.9 (range 53.8-74.0); FS median 37.0 (range 27-46); GLS median -24.4 (range -30 - -18)  - At end of induction (N=41) LVEF (T) median 68.5 (range 48-79); LVEF (S) median 62.1 (range 54-71); FS median 37.3 (range 24-46); GLS median -22.5 (range -27.5 - -17.2)  - At end of consolidation (N=40) LVEF (T) median 66.0 (range 56-74); LVEF (S) median 63.0 (range 54.8-67.0); FS median 36.0 (range 29-43); GLS median -23.3 (range -31.0 - -19.6)  - At end of reinduction (N=36) LVEF (T) median 64.5 (range 46-74); LVEF (S) median 62.1 (range 48-75); FS median 34.1 (range 22-42); GLS median -22.7 (-28.0 - - 17.4)  **ALL Standard risk**  - At diagnosis (N=14) LVEF (T) median 65.5 (range 40-74); LVEF (S) median 60.2 (range 38-65); FS median 34.5 (range 19-43); GLS median -23.4 (range -26.6 - -16.8)  - At end of induction (N=14) LVEF (T) median 70.0 (range 57-77); LVEF (S) median 58.6 (range 55-63.3); FS median 38.5 (range 29-44); GLS median -21.3 (range -25.9 - -16.3)  - At end of consolidation (N=14) LVEF (T) median 65.0 (range 58-72); LVEF (S) median 60.0 (range 54.7-67.7); FS median 35.0 (range 30-40); GLS median -24.1 (range -26.7 - -20.0)  - At end of reinduction (N=14) LVEF (T) median 63.0 (range 60-72); LVEF (S) median 59.0 (range 55.7-67.2); FS median 33.0 (range 31-40); GLS median -23.2 (-31 - - 19)  **AML**  - At diagnosis (N=18) LVEF (T) median 68.0 (range 64-77); LVEF (S) median 62.2 (range 55.5-74.3); FS median 37.0 (range 34-48); GLS median -21 (range -30 - -15)  - At end 7+3 (1^st^ cycle) (N=18) LVEF (T) median 68.0 (range 54-76); LVEF (S) median 63.0 (range 52.2-69.8); FS median 38.0 (range 27-42); GLS median -21.2 (range -26.8 - -17.4)  - At finalizing 7+3 (2^nd^ cycle) (N=18) LVEF (T) median 64.0 (range 38-70); LVEF (S) median 58.8 (range 32-71); FS median 33.0 (range 18-38); GLS median -21.5 (range -27.0 - -15.8)  - At end of treatment (N=16) LVEF (T) median 63.0 (range 43-73); LVEF (S) median 59.9 (range 41.0-64.4); FS median 34.0 (range 21-42); GLS median -22.6 (-26.1 - - 16.1)  Mortality:  Not reported  Risk factors assessed:  Risk factor analysis for cardiotoxicity is reported, but method of analysis and whether analysis was univariate or multivariate is unclear  Results of risk factor analyses (not clear whether analyses were multivariate):  High risk leukemia and factors related to cardiotoxicity:  - Presence of systemic arterial hypertension at any time of treatment (OR 8.57, IC95% 1.35-54.35, P=0.022)  - Pulmonary hypertension (OR 4.11, IC95% 1.12-15.05, P=0.038)  - Global longitudinal myocardial deformation delta difference greater than 15% (OR 19.5, CI95% 4.57-83.09, P<0.000)  - Decrease in LVEF (S) by more than ten percentage points with respect to baseline evaluation by echocardiogram (OR 56, CI95% 6.31-496, P<0.000 | Selection bias: Low risk, original cohort unclear, but ‘participants were selected by non-probabilistic sequential convenience sampling’  Attrition bias:  Low risk for echocardiography for the first two evaluations (111/112 = 99.1% and 102/112 = 91.1%, respectively), high risk for echocardiography for the last evaluation (83/112 = 74.1%).  Unclear risk for biomarker analysis (not stated how many patients had an outcome for this result)  Detection bias: Unclear risk for echocardiography, blinding of assessors not stated. Low risk for laboratory tests, blinding of assessors not mentioned but since blinding has no influence on laboratory results we scores this risk of bias as low.  Confounding: Not applicable (method of risk factor analyses is unclear)  Reporting bias (study group): Not well-defined (no infusion duration of anthracyclines mentioned, no changes in cumulative anthracycline dose (due to circumstances as toxicity) mentioned)  Reporting bias (follow-up): Not well-defined (no length of follow-up provided)  Reporting bias (outcome): Well-defined (method of detection and definition of abnormal outcome provided)  Risk estimation analyses: Not applicable (method of risk factor analyses is unclear)  Funding of the trial:  Supported by Universidad Nacional de Colombia-Bogotá Research Division, Hermes project #37523 and the Research Office of HOM Fundación Hospiral de La Misericordia, Bogot’-Colombia.  Overlap with other included studies:  Not presumed |

| **What is the frequency of occurrence of acute and early-onset cardiotoxicity, as diagnosed by clinical, echocardiographic and biochemical parameters routinely used in clinical practice, in children with cancer treated with anthracyclines, mitoxantrone and/or radiotherapy involving the heart? What are the risk factors?** | | | | |
| --- | --- | --- | --- | --- |
| *Mavinkurve-Groothuis et al.: Myocardial 2D strain echocardiography and cardiac biomarkers in children during and shortly after anthracycline therapy for acute lymphoblastic leukaemia (ALL): a prospective study. European Heart Journal – Cardiovascular Imaging (2013) 14: 562–569* | | | | |
| **Study design; treatment era; follow-up** | **Participants** | **Treatment** | **Diagnostic test; main outcomes** | **Risk of bias assessment** |
| Study design:  Prospective single-center cohort study.  Treatment era:  Not reported  Follow-up:  From start of therapy to 1 year after end of chemotherapy, not further specified | Type and number of participants:  N = 70 newly diagnosed patients with childhood acute lymphoblastic leukaemia (ALL) were asked informed consent  N = 65 were included in the study (N = 4 refused informed consent, N = 1 congenital heart disease)  N = 60 children included in analyses (N = 3 altered treatment because of treatment-related toxicity, N = 2 died)  N = 11/60 children not available for analyses at T = 2 (N = 3/11 died after T = 1, N = 1/11 lost to follow-up due to move abroad, N = 1/11 parents refused tests, N = 6/11 follow-up not available at T = 2)  Original cohort unknown  Diagnosis:  ALL  Age at cancer diagnosis:  Mean age 6 years (range 2.2 – 15.4)  Gender:  Male N = 37/60 (61.7%)  Female N = 23/60 (38.3%)  Controls:  N = 60 age-matched controls ‘routinely referred for echocardiographic evaluation of an asymptomatic, innocent heart murmur or for screening purposes’  Mean age 5.8 years (range 2.2 – 14.7).  Male N = 40/60 (66.7%)  Female N = 20/60 (33.3%)  Cardiovascular risk factors:  Not reported  Prior cardiotoxic treatment:  Not applicable  Prior cardiac dysfunction:  None (exclusion criteria mentions ‘cardiac disease prior to therapy’)  Prior cardioprotective interventions:  Not reported | Anthracyclines:  *Per protocol:*  **T = 0** all patients cumulative anthracycline dose 0 mg/m^2^  **T = 1** all patients cumulative anthracycline dose 120mg/m^2^  **T = 2**,  - N = 20/60 (33.3%) standard risk (SR) patients cumulative anthracycline dose 120mg/m^2^  - N = 30/60 (50%) medium risk (MR) patients cumulative anthracycline dose 300mg/m^2^  - N = 10/60 (16.7%) high risk (HR) patients  cumulative anthracycline dose 240mg/m^2^ + 18mg/m^2^ idarubicin  *Actually received:*  N = 6/10 (60.0%) HR patients completed T = 2.  - N = 4/6 (66.7%) received per protocol anthracycline dose (mentioned above)  - N = 1/6 (16.7%) received cumulative anthracycline dose 120mg/m^2^ (proceeded to stem cell transplant (SCT))  - N = 1/6 (16.7%) received cumulative anthracycline dose 120mg/m^2^ + 18mg/m^2^ idarubicin (proceeded to SCT)  Infusion duration not reported.  Mitoxantrone:  N = 6/10 (60.0%) HR patients completed T = 2.  - N = 4/6 (66.7%) received cumulative mitoxantrone dose 52.5mg/m^2^  - N = 1/6 (16.7%) received cumulative mitoxantrone dose 26.25mg/m^2^ (proceeded to SCT)  Radiotherapy involving the heart:  All SCT (N = 2 /60, 3.3%) patients received total body irradiation | Diagnostic test used for cardiotoxicity assessment:  Laboratory tests: cardiac troponin T (cTnT) and N-terminal-pro-brain natriuretic peptide (NT-pro-BNP).  Echocardiography.  Timing of the diagnostic test:  Before first anthracycline dose (**T = 0**), at the end of induction phase (10 weeks after start of treatment and 5 weeks after latest anthracycline dose, **T = 1**) and 1 year after start of treatment (at least 2 weeks after last anthracycline dose, **T = 2**).  Outcome definitions:  Normal cardiac cTnT was defined as ≤0.01ng/ml.  Normal NT-pro-BNP was based on age-dependent reference values (97.5^th^ percentile).  Abnormal fractional shortening (FS) defined as below 28%  Outcome assessors blinded: Not reported  Occurrence of acute cardiotoxicity:  Not reported  Occurrence of early-onset cardiotoxicity:  *Dichotomous results*  N = 0/60 (0%) showed clinical signs of cardiac failure or abnormal fractional shortening.  **T = 0**, patients N = 60, biomarker samples N = 46/60 (76.7%), echocardiography N = 60/60 (100%)  **T = 1**, patients N = 60, biomarker samples N = 45/60 (75.0%), echocardiography N = 60/60 (100%)  **T = 2**, patients N = 49, biomarker samples N = 41/49 (83.7%), echocardiography N = 49/49 (100%)  N = 0/46 (0%) showed **abnormal cTnT at** **T = 0**.  N = 5/45 (11.1%) showed **abnormal cTnT at** **T = 1**.  N = 1/41 (2.4%) showed **abnormal cTnT at** **T = 2**.  McNemar test based on 37 paired available observations at T = 1 and T = 2: *P* = 0.2  N = 12/46 (26.1%) showed **abnormal NT-pro-BNP at T = 0**.  N = 6/45 (13.3%) showed **abnormal NT-pro-BNP at T = 1**.  N = 8/41 (19.5%) showed **abnormal NT-pro-BNP at T = 2**.  McNemar test based on 37 paired available observations at T = 1 and T = 2: *P* = 0.5  23% had a decreased FS of more than 10% (not further specified in which group / at which time-point.  *Continuous parameters*  **cTnT**:  - **T = 0** median 0.01ng/mL  - **T = 1** median 0.01ng/mL (range 0.01 – 0.04)  - **T = 2** median 0.01ng/mL (range 0.01 – 0.02) Wilcoxon signed ranks test on 37 paired available observations at T = 1 and T = 2: *P* = 0.08.  **NT-pro-BNP**:  - **T = 0** median 13pmol/L (range 2 – 185)  - **T = 1** median 10pmol/L (range 1 – 45),  - **T = 2** median 11pmol/L (range 1 – 68).  Wilcoxon signed ranks test on 37 paired available observations at T = 1 and T = 2: *P* = 0.05.  **FS:**  - **T = 0** mean 40% ± 5 SD,  - **T = 1** mean 36% ± 3 SD  - **T = 2** mean 35% ± 3 SD. *P* <0.0001.  **E/A ratio:**  - **T = 0** mean 1.8 ± 0.6,  - **T = 1** mean 1.8 ± 0.5 SD,  - **T = 2** mean 1.8 ± 0.6 SD. *P* = 0.8.  **Global longitudinal strain (SL):**  - **T = 0** mean -18.2% ± 3.1 SD  - **T = 1** mean -17.3% ± 3.6 SD  - **T = 2** mean -16.7% ± 5.2 SD.  *P* = 0.5.  Mortality:  Before T = 1 N = 2/65 (3.1%) died (progressive disease and sepsis).  Between T = 1 and T = 2 N = 3/60 (5.0%) died (one sepsis, two relapses)  Risk factors assessed: No  Results of multivariate analyses: Not applicable | Selection bias:  Unclear risk (60/70 (85.7%) of patients asked for informed consent included, furthermore original cohort unknown).  Attrition bias:  High risk for laboratory tests (only for 76.7%, 75.0% and 83.7% of the patients were biomarkers available at T = 0, T = 1 and T = 2, respectively)  High risk for echocardiography (at T = 2 only 49/60 (81.7%) of the patients had an outcome assessment)  Detection bias:  Unclear risk for echocardiography (blinding of assessors not described).  Low risk for laboratory tests (no information on blinding of outcome assessors provided, but as blinding is not relevant for outcomes diagnosed by laboratory tests we judged this outcome at low risk of detection bias)  Confounding: Not applicable  Reporting bias (study group): Well-defined (cumulative dosage of anthracyclines given)  Reporting bias (follow-up): Not well-defined (no final length of follow-up mentioned, no mean of follow-up provided)  Reporting bias (outcome):  Well-defined for laboratory tests (method of detection described and definition of abnormal outcome provided). Not well-defined for echocardiography (method of detection described but no definition of abnormal outcome provided for all measures)  Risk estimation analyses:  Not applicable  Funding of the trial:  This study was supported by the Foundation of Childhood Cancer Nijmegen  (‘Stichting Vrienden KOC’).  Overlap with other included studies:  Study group overlaps with Pourier 2020. As largest eligible study group is reported in Mavinkurve-Groothuis 2013, this study is included. |

| **What is the frequency of occurrence of acute and early-onset cardiotoxicity, as diagnosed by clinical, echocardiographic and biochemical parameters routinely used in clinical practice, in children with cancer treated with anthracyclines, mitoxantrone and/or radiotherapy involving the heart? What are the risk factors?** | | | | |
| --- | --- | --- | --- | --- |
| *Moke et al.: Utility of Echocardiography as Screening for Late-onset Anthracycline-induced Cardiotoxicity in Pediatric*  *Cancer Survivors: Observations from the First Decade After End of Therapy. Pediatr Hematol Oncol 2018;40:e283–e288* | | | | |
| **Study design; treatment era; follow-up** | **Participants** | **Treatment** | **Diagnostic test; main outcomes** | **Risk of bias assessment** |
| Study design:  Retrospective cohort study (single center)  Treatment era:  January 2006 – December 2013  Follow-up:  Median follow-up time from end of therapy 5.0 years (range 0 – 18.2) | Type and number of participants:  Original cohort = 435 patients with known anthracycline exposure  N = 11 patients excluded (no anthracycline dose recorded)  N= 56 patients excluded (no echo data reported in chart)  N = 368 patients with known anthracycline dose and echo data  Diagnosis:  N = 159/368 (43.2%) acute lymphoblastic leukemia (ALL)  N = 39/368 (10.6%) acute myeloid leukemia (AML)  N = 39/368 (10.6%) Hodgkin lymphoma  N = 37/368 (10.1%) non-Hodgkin lymphoma  N = 28/368 (7.6%) neuroblastoma  N = 18/368 (4.9%) osteosarcoma  N = 17/368 (4.6%) Wilm’s tumor  N = 10/368 (2.7%) Ewing sarcoma  N = 4/368 (1.1%) rhabdomyosarcoma  N = 4/368 (1.1%) hepatoblastoma  N = 3/368 (0.8%) clear cell renal sarcoma  N = 2/368 (0.5%) myelodysplastic syndrome  N = 2/368 (0.5%) hepatic sarcoma  N = 2/368 (0.5%) undifferentiated sarcomas  N = 1/368 (0.3%) natural killer cell leukemia  N = 1/368 (0.3%) synovial sarcoma  N = 1/368 (0.3%) adrenocortical carcinoma  N = 1/368 (0.3%) renal sarcoma  Age at cancer diagnosis:  Median age 5.3 years (range 0 – 18.3) mentioned in table, median age 5.1 years mentioned in text  Gender:  Male = 201/368 (54.6%)  Female = 167/368 (45.5%)  Controls: Not applicable  Cardiovascular risk factors:  Data on diabetes, hypertension, hyperlipidemia and family history extracted but not reported  Prior cardiotoxic treatment: Not reported  Prior cardiac dysfunction: Data on preexisting heart disease extracted but not reported  Prior cardioprotective interventions:  Not reported | Anthracyclines:  Median cumulative anthracycline dose 200 mg/m^2^ (range 25-515)  Infusion duration not reported  Anthracycline equivalent dosing: doxorubicin x1, daunorubicin x0.833, epirubicin x0.67, idarubicin x5, mitoxantrone x4  Mitoxantrone:  Not separately reported, included as anthracycline equivalent (see above)  Radiotherapy involving the heart:  N = 47/368 (12.8%) had radiation exposure to their chest or neck. Median maximum dose exposure 1950 cGy (range 1000-4500). | Diagnostic test used for cardiotoxicity assessment:  Echocardiography  Timing of the diagnostic test:  Not clearly stated  Outcome definitions:  **Cardiotoxicity** is defined as a composite of any significant echocardiographic change (fractional shortening (FS) <25%, ejection fraction (EF) <50% or absolute 10% decrease in FS or EF from patient baseline, ventricular dilation or cardiomyopathy, E/A reversal, valve abnormality, or arrhythmia) that also led to either formal cardiology referral or cardiac medication initiation  **Early-onset cardiotoxicity** was defined as cardiotoxicity  during treatment up to ≤1 year after end of treatment (EOT).  Outcome assessors blinded: Not reported  Occurrence of acute cardiotoxicity:  Not reported  Occurrence of early-onset cardiotoxicity:  Total of **N = 8/368 (2.2%) early-onset cardiotoxicity**  N = 5/8 early onset cardiotoxicity with initiation of cardiac medication.  N = 3/8 early-onset cardiotoxicity with cardiology referral to without further intervention  Median age at diagnosis for early-onset cardiotoxicity group = 11 years(range 0.8 – 17.8), median follow-up time from EOT = 0 years (range 0 – 0.7), median total anthracycline dose 275 mg/m^2^ (range 100-457).  Mortality: Not reported  Risk factors assessed: No  Results of multivariate analyses: Not applicable | Selection bias:  High risk (368/435 (84.6%) of original cohort incuded)  Attrition bias:  Low risk (368/368 (100%) had at least one outcome assessment for echocardiography results)  Detection bias: Unclear risk, blinding of assessors of echocardiography not mentioned  Confounding: Not applicable  Reporting bias (study group): Well-defined (mean and range of cumulative dosage of anthracyclines and radiotherapy given)  Reporting bias (follow-up): Well defined (length of follow-up defined)  Reporting bias (outcome): Well-defined (method of detection and definition of abnormal outcome provided)  Risk estimation analyses: Not applicable  Funding of the trial: Not reported  Overlap with other included studies:  Not presumed |

| **What is the frequency of occurrence of acute and early-onset cardiotoxicity, as diagnosed by clinical, echocardiographic and biochemical parameters routinely used in clinical practice, in children with cancer treated with anthracyclines, mitoxantrone and/or radiotherapy involving the heart? What are the risk factors?** | | | | |
| --- | --- | --- | --- | --- |
| *Moussa et al.: Risk stratification and pattern of cardiotoxicity in pediatric Ewing sarcoma. Journal of the Egyptian National Cancer Institute 29 (2017) 53–56* | | | | |
| **Study design; treatment era; follow-up** | **Participants** | **Treatment** | **Diagnostic test; main outcomes** | **Risk of bias assessment** |
| Study design:  Retrospective cohort study (single center)  Treatment era:  July 2007 – December 2011  Follow-up:  Median follow-up of 20.3 months (range 4 – 49 months) | Type and number of participants:  N = 149 patients, ‘all patients with newly diagnosed Ewing sarcoma and treated at Children’s Cancer Hospital-Egypt’  Diagnosis:  Ewing sarcoma  Age at cancer diagnosis:  Median age 11 years (range 1-18)  Gender:  Male N = 88/149 (59.1%)  Female N = 61/149 (40.9%)  Controls:  Not applicable  Cardiovascular risk factors: Not reported  Prior cardiotoxic treatment:  Not reported  Prior cardiac dysfunction:  None (exclusion criteria mentions ‘congenital heart disease or cardiac dysfunction’)  Prior cardioprotective interventions:  Not reported | Anthracyclines:  Doxorubicin 75 mg/m^2^ over a 4-6 hours intravenous infusion every 6 weeks (maximum dose 375 mg/m2).  Median cumulative dose (doxorubicin) of 375 mg/m^2^ (range 150-375)  Patients who were scheduled to receive mediastinal or lung radiotherapy received a maximum doxorubicin dose of 300 mg/m^2^.  Mitoxantrone:  Not applicable  Radiotherapy involving the heart:  N = 15/149 (10.1%) received radiotherapy involving the heart  - N = 4/15 (26.7%) received 5580 cGy to the mediastinum  - N = 11/15 (73.3%) received whole-lung irradiation at a dose of 1500 cGy | Diagnostic test used for cardiotoxicity assessment:  Echocardiography  Timing of the diagnostic test:  Cardiac assessment was done at initial presentation; during treatment at weeks 12, 18 and 30; at the end of therapy and annually during follow-up.  Outcome definitions:  **New York Heart Association (NYHA) classification**: class I, cardiac disease, but no symptoms or limitation; class II, mild symptoms and slight limitation; class III, marked limitation  in activity owing to symptoms; and class IV, severe limitation, with  symptoms during rest.  Grading of **cardiac toxicity** based on Common Terminology Criteria for Adverse Events (CTCAE) version 4.0: grade 1, resting ejection fraction  (EF) 58–50% with <10% drop from baseline; grade 2, resting EF 49–40% with 10–19% drop from baseline; grade 3, resting EF 39–20% with ≥20% drop from baseline; grade 4, resting EF <20%; and grade 5, death due to cardiac toxicity.  **Normal left ventricle shortening fraction (LV SF)** defined as >28%  **Normal LV EF** defined as >58%  ‘The onset of developing cardiotoxicity was classified as acute (during protocol treatment) or chronic, and as early (within 1 year from the end of protocol treatment) or late (developed >1 year from the end of protocol treatment)’.  As these definitions slightly differ from our definitions, outcomes will be reported together  Outcome assessors blinded: Not reported  Occurrence of acute and early-onset cardiotoxicity (not separated): *Dichotomous results*  N = 31/149 (20.8%) developed acute or early-onset cardiotoxicity.  - N = 17/31 (54.8%) acute-onset cardiotoxicity  - N = 14/31 (45.2%) early-onset chronic cardiotoxicity  Grading of cardiotoxicity not separated for early- and late onset cardiotoxicity.  Continuous results not separated for early-onset and late onset cardiotoxicity.  Mortality:  N = 7/149 (4.7%) died from acute-onset cardiotoxicity  Risk factors assessed: No  Results of multivariate analyses:  Not applicable | Selection bias:  Unclear risk (N = 149 described as ‘all patients’, but not described whether any patients were excluded)  Attrition bias:  Unclear risk (no information about number of echocardiograms performed)  Detection bias:  Unclear risk (no information about blinding of assessors for echocardiography or clinical assessment provided)  Confounding: Not applicable  Reporting bias (study group): Well-defined (cumulative dose of anthracyclines provided)  Reporting bias (follow-up): Well-defined (length of follow-up mentioned)  Reporting bias (outcome): Well-defined (method of detection and definition of abnormal outcome provided)  Risk estimation analyses: Not applicable  Funding of the trial:  Not reported  Overlap with other included studies:  Not presumed |

| **What is the frequency of occurrence of acute and early-onset cardiotoxicity, as diagnosed by clinical, echocardiographic and biochemical parameters routinely used in clinical practice, in children with cancer treated with anthracyclines, mitoxantrone and/or chest-directed radiotherapy? What are the risk factors?** | | | | |
| --- | --- | --- | --- | --- |
| *Moyo et al.: Early doxorubicin cardiotoxicity in Malawian children admitted to Queen Elizabeth Cental Hospital, Malawi. Pediatric Blood & Cancer, 2021; 68e29003.* | | | | |
| **Study design; treatment era; follow-up** | **Participants** | **Treatment** | **Diagnostic test; main outcomes** | **Risk of bias assessment** |
| Study design:  Prospective cohort study (single center, Malawi)  Treatment era:  January 2016 to June 2019.  Follow-up:  From baseline up to 12 month follow up, not further specified. | Type and number of participants:  N = 202 children admitted and screened  N = 110 excluded from study (N=54 no doxorubicin, N=17 died before recruitment, N=17 doxorubicin before recruitment, N=11 no consent, N=6 low cardiac output, N=5 abandoned medical treatment)  **N = 92 Children aged 3 months to 18 years with cancer enrolled in study**  N = 77 Children available for follow-up at 1 month  N = 44 Children available for follow-up at 6 months  N = 22 Children available for follow-up at 12 months  Diagnosis:  N = 38/92 (41.3%) Burkitt lymphoma  N = 18/92 (19.6%) acute lymphoblastic leukemia (ALL)  N = 12/92 (13.0%) Wilms tumor  N = 24/92 (26.1%) Others  Age at cancer diagnosis:  Mean age 7.4 years ± 3.78 SD.  Gender:  Male N = 68/92 (73.9%)  Female N = 23 /92 (26.1%)  Controls:  Not applicable  Cardiovascular risk factors:  Not reported  Prior cardiotoxic treatment:  None (patients who received doxorubicin before recruitment excluded)  Prior cardiac dysfunction:  None (exclusion criteria mentions ‘Children with pre-existing cardiac conditions’)  Prior cardioprotective interventions:  Not reported | Anthracyclines:  Doxorubicin cumulative dose (mg/m^2^)  N = 33/92 (35.9%) <100  N = 48/92 (52.2%) 100-200  N = 8/92 (8.7%) >200-300  N = 3/92 (3.3%) 300  Infusion duration not reported.  Mitoxantrone:  Not reported  Radiotherapy involving the heart:  Not reported | Diagnostic test used for cardiotoxicity assessment:  Transthoracic echocardiogram  Timing of the diagnostic test:  Echocardiography: at baseline (24-48 hours before initiation chemotherapy), 1 month follow-up, 6 month follow-up and 12 month follow-up.  Outcome definitions:  Cardiotoxicity defined as a decline in left ventricular ejection fraction (LVEF) of ≥10% to a final value of <50%.  Acute cardiotoxicity defined as occurring after a single dose or course treatment.  Early-onset chronic defined as occurring within 1 year.  Outcome assessors blinded: Not reported  Occurrence of acute cardiotoxicity:  Not separated from early-onset cardiotoxicity  Occurrence of early-onset cardiotoxicity:  N = 0/92 (0%) developed cardiotoxicity (according to definition set) during their follow-up  N = 5/77 (6.5%, 95% CI 2.1-14.5) of children who had at least one follow-up experienced LVEF reduction of >10% (but not to a final value of <50%), all cumulative doxorubicin dose between 100 and 300 mg/m^2^.  (N=4 within the first month of follow-up, N=1 by 6 months of follow-up)  N = 1/77 (1.3%) showed a decline in LVEF to a final value of <50% at 1 month follow-up.  Mortality:  N = 9/92 (9.8%) patients died before follow-up at 1 months  N = 30/92 (32.6%) patients died before follow-up at 6 months  N = 36/92 (39.1%) patients died before follow-up at 12 months    Of above: N=31/36 (86.1%) deaths were disease-related, N=5/36 (13.9%) were due to sepsis, N = 0/36 (0%) were cardiovascular deaths.  Risk factors assessed:  ‘None of the baseline characteristics of age, sex, type of malignancy, nutrition status, and administered cumulative dose of doxorubicin were associated with the observed early LVEF changes.’  Results of multivariate analyses:  Not reported | Selection bias:  High risk (92/148, 62.2% of children that would possibly receive doxorubicin included)  Attrition bias:  Low risk for echocardiography follow-up at 1 month (77/83, 92.8% of patients alive), high risk for echocardiography follow-up at 6 months (44/62, 71.0% of patients alive) and at 12 months (22/56, 39.3% of patients alive).  Detection bias: Unknown risk (blinding of assessors for echocardiography not mentioned)  Confounding: Not applicable  Reporting bias (study group): Well-defined (information about cumulative doses provided)  Reporting bias (follow-up): Well-defined (length of follow-up mentioned))  Reporting bias (outcome): Well-defined (method of detection and definition of abnormal outcome provided)  Risk estimation analyses: Not applicable  Funding of the trial:  ‘Dominic Moyo received funding from the ELMA Philanthropy as part of the Paediatric and Child Health Specialist Training Programme’  Overlap with other included studies:  Not presumed |

| **What is the frequency of occurrence of acute and early-onset cardiotoxicity, as diagnosed by clinical, echocardiographic and biochemical parameters routinely used in clinical practice, in children with cancer treated with anthracyclines, mitoxantrone and/or radiotherapy involving the heart? What are the risk factors?** | | | | |
| --- | --- | --- | --- | --- |
| *Oztarhan et al.: The Value of Echocardiography Versus Cardiac Troponin I levels in the Early Detection of Anthracycline Cardiotoxicity in Childhood Acute Leukemia: Prospective Evaluation of a 7-Year-Long Clinical Follow-up. Pediatric Hematology and Oncology, 2011; 28:5, 380-394* | | | | |
| **Study design; treatment era; follow-up** | **Participants** | **Treatment** | **Diagnostic test; main outcomes** | **Risk of bias assessment** |
| Study design:  Single center prospective cohort study  Treatment era:  Phase 1 = March 2002 – February 2003  Phase 2 = September 2003 – April 2004  Phase 3 = January 2005 – June 2009  Follow-up:  From before to after the last cycle of chemotherapy (not further specified) | Type and number of participants:  N = 276 pediatric acute lymphoblastic leukemia (ALL) patients  Original cohort unclear.  **Phase 1, N = 25**  Mean age: 4.8 ± 3.0 year  N = 13/25 (52%) males  N = 12/25 (48%) females  N = 20/25 (80%) ALL-L1 N = 4/25 (16%) ALL-L2 N = 1/25 (4%) ALL-L3  **Phase 2, N = 35**  Mean age: 5.2 ± 2.3 years  N = 15/35 (42.9%) males  N = 20/35 (57.1%) females  N = 20/35 (57.1%) ALL-L1  N = 15/35 (42.9%) ALL-L2  **Phase 3, N=216**  Mean age: 5.85 ± 2.87 years  N = 102/216 (47.2%) males  N = 114/216 (52.8%) females  N = 180/216 (83.3%) ALL-L1  N = 36/216 (16.7%) ALL-L2  ‘There was no significant difference in study groups in terms of age, gender, and body surface area in the 3 phases of the study.’  Diagnosis:  ALL  Age at cancer diagnosis:  See above  Gender:  See above  Controls:  N = 15 healthy children  Mean age: 5.8 ± 2.9 years  N = 7/15 (46.7%) males  N = 8/15 (53.3%) females  Cardiovascular risk factors:  Not reported  Prior cardiotoxic treatment:  None (‘lacking past history of anthracycline-derived chemotherapeutics’)  Prior cardiac dysfunction:  None (‘lacking […] past history of cardiological disease’)  Prior cardioprotective interventions:  Not applicable | Anthracyclines:  Anthracycline at 30 to 350 mg/m^2^ in the first phase an 30 to 240 mg/m^2^ in later phases (Berlin-Frankfurt-Munich (BFM)-2000 protocol).  Doxorubicin and daunorubicin, 1-hour slow intravenous (IV) infusion.  ‘Dosages of anthracyclines were standardized as 1mg doxorubicin = 0.8mg daunorubicin.’  **Phase 1**  Group I (N = 15/25) 0 mg/m^2^; pretreatment values  Group II (N = 15/25) 30–60 mg/m^2^  Group III (N = 14/25) 90–120 mg/m^2^  Group IV (N = 10/25) 150–180 mg/m^2^  Group V (N = 8/25) >180 mg/m^2^  **Phase 2**  Group I (N = 18/35) 0 mg/m^2^; pretreatment values  Group II (N = 18/35) 30 mg/m^2^  Group III (N = 18/35) 60 mg/m^2^  Group IV (N = 18/35) 90 mg/m^2^  Group V (N = 18/35) 120 mg/m^2^  Group VI (N = 17/35) 150 mg/m^2^  Group VII (N = 17/35) 180 mg/m^2^  Group VIII (N = 17/35) 210 mg/m^2^  Group IX (N = 17/35) 240 mg/m^2^  **Phase 3**  Group I (N = 21/216) 0 mg/m^2^; pretreatment values  Group II (N = 28/216) 30 mg/m^2^  Group III (N = 26/216) 60 mg/m^2^  Group IV (N = 23/216) 90 mg/m^2^  Group V (N = 24/216) 120 mg/m^2^  Group VI (N = 25/216) 150 mg/m^2^  Group VII (N = 25/216) 180 mg/m^2^  Group VIII (N = 23/216) 210 mg/m^2^  Group IX (N = 21/216) 240 mg/m^2^  Anthracycline-dependent cardiac alterations were evaluated for 4 dose groups in phase 3,  N = 21/216 (9.7%), group I (0 mg/m^2^; pretreatment values)  N = 77/216 (35.6%), group II (30–90 mg/m^2^)  N = 74/216 (34.3%), group III (120–180 mg/m^2^)  N = 44/216 (20.4%), group IV (≥210 mg/m^2^)  Mitoxantrone:  Not reported  Radiotherapy involving the heart:  Not reported | Diagnostic test used for cardiotoxicity assessment:  Echocardiography and laboratory test (cardiac troponin I, cTnI, levels)  Timing of the diagnostic test:  Echocardiography: Before, during and after the last cycle of chemotherapy.  cTnI levels: prior to, 4 hours after and 24 hours after administration of chemotherapy  Outcome definitions:  Diastolic dysfunction = E/A levels <1  Left ventricle (LV) dysfunction defined as either fractional shortening (FS) below 30% of baseline or a decline of 15% or more from the baseline  Normal cTnI limits = 0 – 0.03 ng/mL)  Outcome assessors blinded:  Echocardiography: Yes (blinded to cumulative anthracycline dose and cTnI levels)  Laboratory tests: Not reported  Occurrence of acute and early-onset cardiotoxicity (not separated): *Continuous results for echocardiography*  **Phase 3**  Control (N=15), Mean E/A = 1.74±0.14 SD.  Group I (N=21/216), Mean E/A = 1.34±0.28 SD.  Group II (N=77/216), Mean E/A = 1.23±0.27 SD.  Group III (N=74/216), Mean E/A = 1.21±0.23 SD.  Group IV (N=44/216), Mean E/A 1.21±0.28 SD.  Control (N=15), Mean EF = 76.13±4.68 SD, P<0.05 compared to group I and IV.  Group I (N=21/216), Mean EF = 81.90±4.13 SD.  Group II (N=77/216), Mean EF = 81.11±5.51 SD.  Group III (N=74/216), Mean EF = 78.48±6.88 SD.  Group IV (N=44/216), Mean EF 75.97±6.69 SD, P<0.05 compared to group I and IV.  Control (N=15), Mean FS = 38.46±3.83 SD, P<0.05 compared to group I and IV.  Group I (N=21/216), Mean FS = 43.19±4.19 SD.  Group II (N=77/216), Mean FS = 43.19±5.28 SD.  Group III (N=74/216), Mean FS = 41.31±7.01 SD.  Group IV (N=44/216), Mean FS 40.15±6.11 SD.  *Continuous results for laboratory tests*  **Phase II**  Group I (N = 18/35) 0 mg/m^2^, cTnI (4-hour) mean 0.024±0.03 SD and cTnI (48-hour) mean 0.022±0.03 SD.  Group II (N = 18/35) 30 mg/m^2^, cTnI (4-hour) mean 0.027±0.05 SD and cTnI (48-hour) mean 0.025±0.04 SD.  Group III (N = 18/35) 60 mg/m^2^, cTnI (4-hour) mean 0.018±0.04 SD and cTnI (48-hour) mean 0.021±0.03 SD.  Group IV (N = 18/35) 90 mg/m^2^, cTnI (4-hour) mean 0.025±0.05 SD and cTnI (48-hour) mean 0.026±0.03 SD.  Group V (N = 18/35) 120 mg/m^2^, cTnI (4-hour) mean 0.035±0.03 SD and cTnI (48-hour) mean 0.024±0.03 SD.  Group VI (N = 17/35) 150 mg/m^2^, cTnI (4-hour) mean 0.023±0.03 SD and cTnI (48-hour) mean 0.022±0.02 SD.  Group VII (N = 17/35) 180 mg/m^2^, cTnI (4-hour) mean 0.025±0.03 SD and cTnI (48-hour) mean 0.022±0.03 SD.  Group VIII (N = 17/35) 210 mg/m^2^, cTnI (4-hour) mean 0.019±0.02 SD and cTnI (48-hour) mean 0.020±0.02 SD.  Group IX (N = 17/35) 240 mg/m^2^, cTnI (4-hour) mean 0.018±0.03 SD and cTnI (48-hour) mean 0.036±0.02 SD.  **Phase III**  Group I (N=21/216), 0mg/m^2^, cTnI (4-hours) mean 0.02±0.22 SD.  Group II (N=77/216), 30-90 mg/m^2^, cTnI (4-hours) mean 0.02±0.01 SD and cTnI (48-hour) mean 0.02±0.01 SD.  Group III (N=74/216), 120-180 mg/m^2^, cTnI (4-hours) mean 0.02±0.02 SD and cTnI (48-hour) mean 0.02±0.01 SD.  Group IV (N=44/216), ≥ 210 mg/m^2^, cTnI (4-hours) mean 0.04±0.14 SD and cTnI (48-hour) mean 0.07±0.27 SD.  *Dichotomous results*  **Phase I** = 13% incidence of heart failure  **Phase II** = 5% incidence of heart failure  **Phase III** = 5.5% incidence of heart failure  **Phase II,** N = 32/35 (91.4%) had diastolic dysfunction  **Phase III**, N = 59/216 (27.3%) had diastolic dysfunction  Mortality:  N = 2/35 (5.7%) died in Phase II due to congestive heart failure  N = 12/216 (5.6%) died in Phase III (N = 2 congestive heart failure, N = 2 sepsis, other unknown)  Risk factors assessed: No  Results of multivariate analyses: Not applicable | Selection bias: Unclear risk (original cohort unclear)  Attrition bias: High risk (outcome assessment does not exceed 77/216 = 35.6% (Phase III) for echocardiography and 18/35 = 51.4% (Phase II)/ 77/216 = 35.6% (Phase III) for lab results)  Detection bias:  Low risk for echocardiography (blinding of assessor). Low risk for laboratory tests (no information on blinding of outcome assessors provided, but as blinding is not relevant for outcomes diagnosed by laboratory tests we judged this outcome at low risk of detection bias)  Confounding: Not applicable  Reporting bias (study group): Well-defined (cumulative dosage of anthracyclines given)  Reporting bias (follow-up): Not well-defined (no mean of follow-up given)  Reporting bias (outcome): Well defined (method of detection and definition of abnormal outcome provided)  Risk estimation analyses: Not applicable  Funding of the trial: None reported  Overlap with other included studies:  Not presumed |

| **What is the frequency of occurrence of acute and early-onset cardiotoxicity, as diagnosed by clinical, echocardiographic and biochemical parameters routinely used in clinical practice, in children with cancer treated with anthracyclines, mitoxantrone and/or radiotherapy involving the heart? What are the risk factors?** | | | | |
| --- | --- | --- | --- | --- |
| *Radu et al.: Cardiotoxicity evaluation in pediatric patients with acute lymphoblastic leukemia – results of prospective study. Med Ultrason, 2019, Vol. 21, no. 4, 449-455* | | | | |
| **Study design; treatment era; follow-up** | **Participants** | **Treatment** | **Diagnostic test; main outcomes** | **Risk of bias assessment** |
| Study design:  Prospective non-randomized observational study  Treatment era:  February 2015 – November 2017  Follow-up:  From diagnosis to 1 year after diagnosis, not further specified | Type and number of participants:  N = 70 children diagnosed with acute lymphoblastic leukemia (ALL) during enrolment period,  N = 48 children with ALL included after applying discontinuation criteria  Diagnosis:  N = 39/48 (81.3%) B-cell ALL  N = 9/48 (18.8%) T-cell ALL  Age at cancer diagnosis:  Age groups:  1-5 years N = 24/48 (50%)  6-10 years N = 11/48 (22.9%)  >10 years N = 13/48 (27.1%)  Gender:  Male N = 29/48 (60.4%)  Female N = 19/48 (39.6%)  Controls:  Not applicable  Cardiovascular risk factors:  Not reported  Prior cardiotoxic treatment:  Not reported  Prior cardiac dysfunction:  Not reported  Prior cardioprotective interventions:  Not reported | Anthracyclines:  According to BFM ALL IC 2009 protocol, based on risk stratification, 8-10 doses of 30mg/m^2^ anthracyclines (AC), first dose on day 8 and last one 9 months after diagnosis.  N = 19/48 (39.6%) standard risk.  N = 21/48 (43.8%) intermediate risk  N = 8/48 (16.7%) high risk  Cumulative dose of AC  N = 7/48 (14.6%) <200 mg/m^2^  N = 25/48 (52.1%) 200-240 mg/m^2^  N = 16/48 (33.3%) ≥240 mg/m^2^  No infusion duration reported.  Mitoxantrone:  Not reported  Radiotherapy involving the heart:  Not reported | Diagnostic test used for cardiotoxicity assessment:  Laboratory tests: Troponin I (TnI).  Echocardiography  Timing of the diagnostic test:  Laboratory tests: At diagnosis (TnI1), one hour after first AC dose (TnI2), before the last AC dose (TnI3), one hour after last AC dose (TnI4) and one year after diagnosis (TnI5).  Echocardiography: At diagnosis, after all AC doses and 1 year after diagnosis.  Outcome definitions:  TnI normal range = 0-0.05  pg/dl  Outcome assessors blinded:  Laboratory tests: not stated. Echocardiography: blinded to patients status.  Occurrence of acute cardiotoxicity:  *Continuous results*  **TnI** (pg/dl) T1 median 0.01 (interquartile range (IQR) 0.01-0.01)  **TnI** (pg/dl) T2 median 0.01 (IQR 0.01-0.01)  Occurrence of early-onset cardiotoxicity:  *Continuous results*  **TnI** (pg/dl) T1 median 0.01 (IQR 0.01-0.01)  **TnI** (pg/dl) T3 median 0.015 (IQR 0.01-0.02)  **TnI** (pg/dl) T4 median 0.02 (IQR 0.01-0.03)  **TnI** (pg/dl) T5 median 0.01 (IQR 0.01-0.02) TnI increased after therapy compared to baseline (*P* <0.001).  Echocardiography (N = 46/48, 95.8%)  **LVEF** (%) at diagnosis median 63 (IQR 60.5-65)  **LVEF** (%) after all AC doses median 64.5 (IQR 60-67)  **LVEF** (%) one year after diagnosis median 62 (IQR 60-65)  *P* =0.833  **E/A** at diagnosis median 1.5 (IQR 1.2-1.9)  **E/A** after all AC doses median 1.4 (IQR 1.2-1.6)  **E/A** one year after diagnosis median 1.4 (IQR 1.2-1.9)  *P* =0.031  Mortality:  None within studied group (‘The discontinuation criteria from the study were death of any cause’)  Risk factors assessed: No  Results of multivariate analyses: Not applicable | Selection bias:  High risk (48/70 (68.6%) finalized the study protocol after discontinuation criteria)  Attrition bias:  Low risk for laboratory tests (all included patients had laboratory measurements). Low risk for echocardiography (46/48, 95.8% had an outcome for this parameter)  Detection bias:  Low risk for echocardiography (assessors were blinded to patient’s status). Low risk for laboratory tests (no information on blinding of outcome assessors provided, but as blinding is not relevant for outcomes diagnosed by laboratory tests we judged this outcome at low risk of detection bias)  Confounding: Not applicable  Reporting bias (study group): Not well-defined (no infusion duration mentioned, no mean of cumulative dose mentioned)  Reporting bias (follow-up): Not well defined (no mean of follow-up provided)  Reporting bias (outcome):  Not well-defined (method of detection and definition of abnormal outcome provided but not reported for laboratory test, no definition of abnormal outcome reported for echocardiography outcomes)  Risk estimation analyses: Not applicable  Funding of the trial: None reported  Overlap with other included studies:  Not presumed |

| **What is the frequency of occurrence of acute and early-onset cardiotoxicity, as diagnosed by clinical, echocardiographic and biochemical parameters routinely used in clinical practice, in children with cancer treated with anthracyclines, mitoxantrone and/or radiotherapy involving the heart? What are the risk factors?** | | | | |
| --- | --- | --- | --- | --- |
| *Sági et al.: Possible roles of genetic variations in chemotherapy related cardiotoxicity in pediatric acute lymphoblastic leukemia and osteosarcoma. BMC Cancer, 2018: 18:704* | | | | |
| **Study design; treatment era; follow-up** | **Participants** | **Treatment** | **Diagnostic test; main outcomes** | **Risk of bias assessment** |
| Study design:  Multi-center retrospective cohort study.  Treatment era:  1989 – 2015  Follow-up:  From diagnosis up to 15 years after diagnosis, not further specified. | Type and number of participants:  Original cohort N = 680 pediatric patients with acute lymphoblastic leukemia (ALL) or osteosarcoma (OSC).  N = 19 excluded (N = 7 Down syndrome, N = 12 previous cardiac problems/concomitant disease with potential cardiac complications)  N = 661 included patients  Diagnosis:  N = 622/661 (94.1%) ALL  N = 39/661 (5.9%) OSC  Age at cancer diagnosis:  Mean age all patients = 6.6 years ± 4.3 SD (median 5.3, range 0-18)  Mean age ALL patients = 6.39 years ± 4.3 SD (median 5.2, range 0-18)  Mean age OSC patients =  13.1 years ± 3.5 SD (median 13.2, range 5-18)  Gender:  All patients  Male N = 399/661 (60.4%)  Female N = 262/661 (39.6%)  ALL patients  Male N = 372/622 (59.8%)  Female N = 250/622 (40.2%)  OSC patients  Male N = 27/39 (69.2%)  Female N = 12/39 (30.8%)  Controls:  Not applicable  Cardiovascular risk factors:  Not reported  Prior cardiotoxic treatment:  Not reported  Prior cardiac dysfunction:  None (exclusion criteria mentions ‘previous cardiac problems or any concomitant disease with potential cardiac complications’)  Prior cardioprotective interventions:  Not reported | Anthracyclines:  ***All patients (N = 661)***  Anthracycline dose range 60-840mg/m^2^. N = 463/661 (70.0%) ≤240mg/m^2^  N = 196/661 (29.7%) >240mg/m^2^  *2 patients are missing/not described*  ***ALL patients (N = 622)***  Anthracycline dose range 60-840mg/m^2^. N = 457/622 (73.5%) ≤240mg/m^2^  N = 163/622 (26.2%) >240mg/m^2^  *2 patients are missing/not described*  N = 165/622 (26.5%) standard risk (cumulative anthracycline dose range 180-240mg/m^2^)  N = 355/622 (57.1%) intermediate risk (cumulative anthracycline dose 180-240mg/m^2^)  N = 100/622 (16.1%) high risk (cumulative anthracycline dose 240-380mg/m^2^).  Anthracyclines administered as doxorubicin or daunorubicin in 3 hours infusion i.v.  ***OSC patients (N = 39)***  Anthracycline dose range 180-360mg/m^2^ N = 6/39 (15.4%) ≤240mg/m^2^  N = 33/39 (84.6%) >240mg/m^2^  N = 3/39 (7.7%) standard risk (cumulative anthracycline dose 360 mg/m^2^)  N = 24/39 (61.5%) intermediate risk (cumulative anthracycline dose not described)  N = 12/39 (30.8%) high risk (cumulative anthracycline dose 180 mg/m^2^).  Infusion duration not reported.  Mitoxantrone:  Not reported  Radiotherapy involving the heart:  Not reported | Diagnostic test used for cardiotoxicity assessment:  Echocardiography  Timing of the diagnostic test:  Echocardiography: at diagnosis (1), acute phase during intensive chemotherapy phase (2), during oral maintenance therapy (3) and at several time points ranging from end of treatment to >15 years from diagnosis (4)-(8)  Outcome definitions:  Pathological fractional shortening (FS) defined as FS ≤ 28%  Outcome assessors blinded: Not reported  Occurrence of acute cardiotoxicity:  Not reported  Occurrence of early-onset cardiotoxicity:  *Continuous results*  **All patients** with echocardiography at diagnosis (N = 387/661), mean FS 41.4% ± 6.0 SD.  All patients with echocardiography <1 year from diagnosis (N = 280/661), mean FS 40.4% ± 6.1 SD.  N = 104 decreased FS (compared to individual value at diagnosis), N = 83 not decreased FS (total not matching up to 280 patients), OR 1.0  **ALL patients** with echocardiography at diagnosis (N = 358/622), mean FS 41.5% ± 6.1 SD.  ALL patients with echocardiography <1 year from diagnosis (N=275/622), mean FS 40.4% ± 6.1 SD.  **OSC patients** with echocardiography at diagnosis (N = 29/39), mean FS 39.6% ± 4.4 SD.  OSC patients with echocardiography <1 year from diagnosis (N = 5/39), mean FS 40.2% ± 5.3 SD.  Mortality:  Not reported  Risk factors assessed:  Effect of genetic polymorphisms on the left ventricular parameters assessed in multivariate analyses, but early and late outcomes not separated  Results of multivariate analyses: Not applicable | Selection bias:  Low risk (661/680, 97.2% or original cohort included)  Attrition bias:  High risk for echocardiography (only 387/661, 58.5% had an outcome for this result)  Detection bias: Unclear risk (blinding of assessors of echocardiography not described)  Confounding: Not applicable  Reporting bias (study group): Not well-defined (range of cumulative dose of anthracyclines given, but no mean of cumulative dose, infusion duration not known for all patients)  Reporting bias (follow-up): Not well-defined, no mean of length of follow-up provided  Reporting bias (outcome): Well-defined, method of detection and definition of abnormal outcome provided  Risk estimation analyses:  Not applicable  Funding of the trial:  This study was supported by National Research, Development and Innovation Office (NKFIH) Grants No. PD109200 (ÁF Semsei) and K115861 (DJ Erdélyi). The funders had no role in study design, data collection and analysis, decision to  publish, or preparation of the manuscript.  Overlap with other included studies:  Not presumed |

| **What is the frequency of occurrence of acute and early-onset cardiotoxicity, as diagnosed by clinical, echocardiographic and biochemical parameters routinely used in clinical practice, in children with cancer treated with anthracyclines, mitoxantrone and/or radiotherapy involving the heart? What are the risk factors?** | | | | |
| --- | --- | --- | --- | --- |
| *Samosir et al.: Risk Factors of Daunorubicine Induced Early Cardiotoxicity in Childhood Acute Lymphoblastic Leukemia: A Retrospective Study. Asian Pac J Cancer Prev, 2021; 22 (5): 1407 – 1412* | | | | |
| **Study design; treatment era; follow-up** | **Participants** | **Treatment** | **Diagnostic test; main outcomes** | **Risk of bias assessment** |
| Study design: Retrospective cohort study (single center)  Treatment era:  January 2014 to April 2019  Follow-up:  Follow-up during first year of anthracycline administration, not further specified. | Type and number of participants:  N = 495 new children aged under 18 years were diagnosed with acute lymphoblastic leukemia (ALL), however, ‘patients that were already treated with daunorubicine according to Indonesian ALL 2013 Protocol and had clear echocardiography data before and at any given time after daunorubicine administration were also included in this study’, therefore original cohort unclear.  N = 49 children met the inclusion criteria.  Diagnosis:  ALL  Age at cancer diagnosis:  Mean age of 9.18 years (range 2-16 years old)  Gender:  Male N = 31/49 (63.3%)  Female N = 18/49 (36.7%)  Controls: Not applicable  Cardiovascular risk factors:  Not reported  Prior cardiotoxic treatment:  Not reported  Prior cardiac dysfunction:  None (Exclusion criteria mentions ‘critical congenital heart defect, and LVEF <53% before chemotherapy’)  Prior cardioprotective interventions:  Not reported | Anthracyclines:  Median cumulative dose of daunorubicine = 143.69mg/m^2^.  ‘All infusion was given through 8 hours of intravenous drip diluted in 250mL NaCl 0.9%.’  Mitoxantrone:  Not reported  Radiotherapy involving the heart:  Not reported | Diagnostic test used for cardiotoxicity assessment:  Echocardiography  Timing of the diagnostic test:  Not reported (within first year of anthracycline administration)  Outcome definitions:  Early cardiotoxicity defined as a decline of left ventricle ejection fraction (LVEF) greater than 10% with a final LVEF <53% during the first year of anthracycline administration.  *However, in another paragraph, the article states:*  Early cardiotoxicity determined by the occurrence of decreasing LVEF >10% or below 50% from the baseline within the first year of daunorubicine chemotherapy.  Outcome assessors blinded: Not reported  Occurrence of acute and early-onset cardiotoxicity (not separated):  N = 5/49 (10.2%) patients developed early anthracycline cardiotoxicity  Mortality:  Not reported  Risk factors assessed: Yes.    ‘Bivariate analysis revealed that ≥4 years, risk group, cumulative dose of 120mg/m^2^ were potentially associated with early cardiotoxicity. The potential risk factors were then introduced to the multivariate analysis and multiple regression logistic.’  Results of multivariate analyses:  Risk factors for daunorubicine-induced early cardiotoxicity:  - Risk of children aged ≥4 years old was 1.128 times higher than children aged <4 years old (prevalence ratio (PR) 1.128; 95% CI 1.015 – 1.254; P<0.001)  - Risk of high-risk group was 1.135 times higher than standard-risk group (PR 1.135; 95% CI 1.016 – 1.269; P<0.001)  - Risk of cumulative dose above 120mg/m^2^ 1.161 times higher than lesser dose (PR 1.161; 95% CI 1.019 – 1.324; P=0.001 | Selection bias: Unclear risk (Original cohort described as 495 new patients with ALL, but unknown number of patients already treated according to protocol included)  Attrition bias:  Low risk (all included patients had outcome assessment since incomplete medical records were an exclusion criteria)  Detection bias: Unclear risk (blinding of assessors of echocardiography not stated)  Confounding:  High risk (not all important prognostic factors taken into account)  Reporting bias (study group): Well-defined (cumulative dosage of anthracyclines and infusion duration provided)  Reporting bias (follow-up): Not well-defined (no timeline of assessment provided, no mean of follow-up given)  Reporting bias (outcome): Well-defined (method of detection and definition of abnormal outcome provided)  Risk estimation analyses:  Well-defined (prevalence ratio provided)  Funding of the trial:  None reported  Overlap with other included studies:  Not presumed |

| **What is the frequency of occurrence of acute and early-onset cardiotoxicity, as diagnosed by clinical, echocardiographic and biochemical parameters routinely used in clinical practice, in children with cancer treated with anthracyclines, mitoxantrone and/or radiotherapy involving the heart? What are the risk factors?** | | | | |
| --- | --- | --- | --- | --- |
| *Schramm et al.: Daunorubicin during delayed intensification decreases the incidence of infectious complications – a randomized comparison in trial CoALL 08-09. Leuk Lymphoma, 2019; 60 (1): 60-68* | | | | |
| **Study design; treatment era; follow-up** | **Participants** | **Treatment** | **Diagnostic test; main outcomes** | **Risk of bias assessment** |
| Study design:  Multi-center randomized comparison trial  Treatment era:  1 October 2010 – 30 September 2016  Follow-up:  Not specified | Type and number of participants:  N = 489 patients with newly diagnosed B-precursor acute lymphoblastic leukemia (ALL) or T-ALL or T-lymphoblastic lymphoma (LBL).  N = 182 excluded  (N = 1 trisomy 21, N = 124 not eligible for randomization because low/high risk, N = 48 event before randomization (stem cell transplant, death, early relapse, severe treatment toxicity), N = 9 no informed consent)  **N = 307 patients randomized**  N = 153/307 (49.8%) randomized for doxorubicin (DOX) treatment  N = 154/307 (50.2%) randomized for daunorubicin (DNR) treatment  Diagnosis:  **DOX** (N=153)  N = 134/153 (87.6%) non-T-cell ALL  N = 8/153 (5.2%) T-ALL  N = 7/153 (4.6%) T-NHL  N = 2/153 (1.3%) B-immunoblastic lymphoma  N = 1/153 (0.7%) missing immunophenotype  N = 1/153 (0.7%) mixed lineage  **DNR** (N=154)  N = 137/154 (89.0%) non T-cell ALL  N = 7/154 (4.5%) T-ALL  N = 9/154 (5.8%) T-NHL  N = 1/154 (0.6%) missing immunophenotype  Age at cancer diagnosis:  **DOX**  Age <10 years N = 117/153 (76.5%)  Age ≥10 years N = 36/153 (23.5%)  **DNR**  Age <10 years N = 115/154 (74.7%)  Age ≥10 years N = 39/154 (25.3%)  Gender:  **DOX**  Male N = 82/153 (53.6%)  Female N = 71/153 (46.4%)  **DNR**  Male N = 91/154 (59.1%)  Female N = 63/154 (40.9%)  Controls:  Not applicable  Cardiovascular risk factors: Not reported  Prior cardiotoxic treatment:  Not reported  Prior cardiac dysfunction:  Not reported  Prior cardioprotective interventions:  Not reported | Anthracyclines:  *Only dosing in delayed intensification reported, not overall anthracycline dose.*  N = 153/307 (49.8%) doxorubicin, 24 hours infusion, 30mg/m^2^ per administration (low risk one block (cumulative dose 60mg/m^2^), high risk two blocks (cumulative dose 120mg/m^2^))  N = 154/307 (50.2%) daunorubicin, 24 hours infusion, 36 mg/m^2^ per administration (low risk one block (cumulative dose 72mg/m^2^), high risk two blocks (cumulative dose 144mg/m^2^))  No mean of cumulative dose given, no alterations in therapy described (i.e. dose reduction etc.)  Mitoxantrone:  Not reported  Radiotherapy involving the heart:  Not reported | Diagnostic test used for cardiotoxicity assessment:  Toxicity criteria (for cardiotoxicity echography and clinical evaluation)  Timing of the diagnostic test: For each 2-week cycle, not further described  Outcome definitions:  Toxicity based on the NCI Common Toxicity Criteria, version 2.0  Cardiac toxicity (function):  Grade 0 = normal  Grade 1 = asymptomatic, ejection fraction (EF) decreased (rest) ≥ 10% but <20% of initial value  Grade 2 = asymptomatic, but EF decreased (rest) below the lower EF standard value (activity) or EF decreased <20% of initial value  Grade 3 = mild congestive heart failure (CHF), therapeutically compensated  Grade 4 = severe / refractory CHF or requiring intubation  Cardiac toxicity (echocardiography, left ventricular shortening fraction (LV-SF))  Grade 0 = ≥30%  Grade 1 = ≥24% <30%  Grade 2 = >15% <20%  Grade 3 = ≤15%  Outcome assessors blinded: Not reported  Occurrence of acute and early-onset cardiotoxicity (not separated):  **DOX**  N = 114/117 (97.4%) grade 0 cardiotoxicity (cardiac function)  N = 2/117 (1.7%) grade 1 cardiotoxicity (cardiac function)  N = 1/117 (0.9%) grade 3 cardiotoxicity (cardiac function)  N = 82/85 (96.5%) grade 0 cardiotoxicity (left ventricular function)  N = 3/85 (3.5%) grade 1 cardiotoxicity (left ventricular function)  **DNR**  N = 110/112 (98.2%) grade 0 cardiotoxicity (cardiac function)  N = 1/112 (0.9%) grade 1 cardiotoxicity (cardiac function)  N = 1/112 (0.9%) grade 3 cardiotoxicity (cardiac function)  N = 90/90 (100%) grade 0 cardiotoxicity (left ventricular function)  Mortality:  Not reported  Risk factors assessed: No  Results of multivariate analyses: Not applicable | Selection bias:  High risk (307/489, 62.8% of original cohort included)  Attrition bias:  High risk (only 117/153, 76.5% and 85/153, 55.6% of DOX group had outcome assessment for cardiotoxicity; only 112/154, 72.7% and 90/154, 58.4% of DNR group had outcome assessment for cardiotoxicity)  Detection bias: Unclear risk, blinding of assessors not mentioned  Confounding: Not applicable  Reporting bias (study group): Not well-defined (no cumulative dose of anthracycline given, only per protocol dose in delayed intensification; no information about dose alterations given)  Reporting bias (follow-up): Not well-defined (no length of follow-up defined)  Reporting bias (outcome): Well-defined (method of detection and definition of abnormal outcome provided)  Risk estimation analyses: Not applicable  Funding of the trial:  None reported  Overlap with other included studies:  Not presumed |

| **What is the frequency of occurrence of acute and early-onset cardiotoxicity, as diagnosed by clinical, echocardiographic and biochemical parameters routinely used in clinical practice, in children with cancer treated with anthracyclines, mitoxantrone and/or radiotherapy involving the heart? What are the risk factors?** | | | | |
| --- | --- | --- | --- | --- |
| *Shaikh et al.: Anthracycline-induced cardiotoxicity: prospective cohort study from Pakistan. BMJ Open, 2013 ;3 :e003663.* | | | | |
| **Study design; treatment era; follow-up** | **Participants** | **Treatment** | **Diagnostic test; main outcomes** | **Risk of bias assessment** |
| Study design:  Prospective cohort study (single center)  Treatment era:  July 2010 – June 2012  Follow-up:  From baseline up to 1 year after therapy  ‘The length of follow-up was measured from the first exposure to anthracycline’ | Type and number of participants:  N = 311 (original cohort) children with anthracycline treatment.  N = 121 ‘not fit to inclusion criteria + not consented’  N = 190 patients enrolled  N = 80/190 (42.1%) excluded (‘poor follow not available for final echocardiography study/self treatment withdrawal/treatment migration’)  **N = 110 children with childhood malignancies**  Diagnosis:  N = 70/110 (63.6%) acute lymphoblastic leukaemia (ALL)  N = 12/110 (10.9%) acute myeloid leukaemia (AML)  N = 19/110 (17.3%) Hodgkin + non-Hodgkin  N = 4/110 Ewing sarcoma (3.6%)  N = 5/110 Wilms tumour (4.5%)  Age at cancer diagnosis:  Mean age 74 months ± 44 SD (median 62 months)  Gender:  Male N = 75/110 (68.2%)  Female N = 35/110 (31.8%)  Controls:  Not applicable  Cardiovascular risk factors:  Trisomy 21 N = 5/110 (4.5%)  - ALL N = 3/70 (4.3%)  - AML N = 2/12 (16.7%)  Prior cardiotoxic treatment:  None (exclusion criteria mentions ‘relapse of cancer’)  Prior cardiac dysfunction: None (exclusion criteria mentions ‘previously diagnosed with any structural heart disease or cardiomyopathy’)  Prior cardioprotective interventions:  Not applicable | Anthracyclines:  N = 59/110 (53.6%) doxorubicin  N = 35/110 (31.8%) combination doxorubicin and daunorubicine  N = 16/110 (14.5%) daunorubicin  Cumulative anthracycline dose (mg/m^2^)  <100, N = 40/110 (36.4%)  100-300, N = 55/110 (50%)  >300, N = 15/110 (13.6%)  Infusion duration not reported  Mitoxantrone:  Not reported  Radiotherapy involving the heart:  N = 24/110 (21.8%) received radiation (unclear where) | Diagnostic test used for cardiotoxicity assessment:  Echocardiography  Timing of the diagnostic test:  Baseline, 1 month and 1 year after chemotherapy.  Outcome definitions:  **Shortening fraction** (SF) normal range 29-38%  **Ejection fraction** (EF) normal >55%  **Tei index** normal range 0.4±0.09  Cardiac dysfunction, systolic dysfunction and diastolic dysfunction not clearly defined  Outcome assessors blinded:  Not reported  Occurrence of acute and early-onset cardiotoxicity (not separated):  **N = 15/110 (13.6%) cardiac dysfunction within 1 month**  - N = 10/15 diastolic dysfunction,  - N = 5/15 combined systolic and diastolic dysfunction  - N = 4/15 signs of cardiac failure  - N = 9/15 pericardial effusion  **N = 28/110 (25.5%) cardiac dysfunction during first year**  - N = 12/110 diastolic dysfunction  - N = 16/110 combined systolic and diastolic dysfunction  - N = 19/110 pericardial effusion  - N = 18/110 signs of cardiac failure  - N = 7/110 death due to cardiac dysfunctions  Continuous parameters  **EF** (%) baseline mean 69.9 ±4.3 SD.  EF (%) within a month mean 67.3 ± 5.3SD.  EF (%) within a year mean 62.6 ± 9.6 SD.  *P* <0.001  **FS** (%) baseline mean 36.6 ± 2.6SD.  FS (%) within a month mean 35.3 ± 2.8SD.  FS (%) within a year mean 32.9 ± 5.0SD.  *P* <0.001  **E/A ratio** baseline mean 1.6 ± 1.8SD.  E/A ratio within a month mean 1.38 ± 0.21SD.  E/A ratio within a year mean 1.3 ± 0.33SD  P<0.001  **Tei index** (Myocardial performance index, MPI) baseline mean 0.3 ± 0.05 SD.  Tei index (MPI) within a month mean 0.4 ± 0.05 SD.  Tei index (MPI) within a year mean 0.4 ± 0.07 SD.  *P* <0.001  **Relationship type of anthracycline and cardiac dysfunction**  *Doxorubicin (N = 59)*  - Cardiac dysfunction within a month N = 8/59 (13.6%) with mean cumulative dose 276mg/m^2^ ± 199SD (versus 107mg/m^2^ ± 73SD in no dysfunction patients, *P* <0.001).  - Cardiac dysfunction within a year N = 9/59 (15.3%) with mean cumulative dose 279mg/m^2^ ± 185SD (versus 104mg/m^2^ ± 69SD in no dysfunction patients, *P* <0.001).  *Daunorubicin (N = 16)*  - Cardiac dysfunction within a month N = 5/16 (31.3%) with mean cumulative dose 315mg/m^2^ ± 114SD (versus 273mg/m^2^ ± 93SD in no dysfunction patients, *P* = 0.440)  - Cardiac dysfunction within a year N = 10/16 (62.5%) with mean cumulative dose 310mg/m^2^ ± 85SD (versus 247mg/m^2^ ± 114SD in no dysfunction patients, *P* = 0.224)  *Combination (N = 35)*  - Cardiac dysfunction within a month N = 2/35 (5.7%) with mean cumulative dose 297mg/m^2^ ± 123SD (versus 158mg/m^2^ ± 69SD in no dysfunction patients, *P* = 0.011)  - Cardiac dysfunction within a year N = 9/35 (25.7%) with mean cumulative dose 228mg/m^2^ ± 81SD (versus 144mg/m^2^ ± 64SD in no dysfunction patients, *P* = 0.004)  Mortality:  N = 7/110 (6.4%) died duo to cardiac dysfunction within one year of therapy  Risk factors assessed: No  Results of multivariate analyses: Not applicable | Selection bias: High risk (110/311, 35.4% of original cohort included in study)  Attrition bias: Low risk (All included patients had an outcome for all parameters (echocardiography 1 month and 1 year after))  Detection bias: Unclear risk, blinding of assessors is not mentioned  Confounding: Not applicable  Reporting bias (study group): Well-defined, (cumulative dose of anthracyclines given)  Reporting bias (follow-up): Not well-defined (no mean of follow-up provided)  Reporting bias (outcome): Not well defined (method of detection provided but no definition of abnormal outcome provided)  Risk estimation analyses: Not applicable  Funding of the trial:  This research received no specific grant from any funding agency in  the public, commercial or not-for-profit sectors.  Overlap with other included studies:  Not presumed |

| **What is the frequency of occurrence of acute and early-onset cardiotoxicity, as diagnosed by clinical, echocardiographic and biochemical parameters routinely used in clinical practice, in children with cancer treated with anthracyclines, mitoxantrone and/or radiotherapy involving the heart? What are the risk factors?** | | | | |
| --- | --- | --- | --- | --- |
| *Stöhr et al.: Comparison of epirubicin and doxorubicin cardiotoxicity in children and adolescents treated within the German Cooperative Soft Tissue Sarcoma Study (CWS). J Cancer Res Clin Oncol (2006) 132: 35–40* | | | | |
| **Study design; treatment era; follow-up** | **Participants** | **Treatment** | **Diagnostic test; main outcomes** | **Risk of bias assessment** |
| Study design:  Prospective multicenter cohort study  Treatment era:  1 January 1998 – 21 December 2002.  Follow-up:  Total group (N = 172): Median follow-up 27.7 months (range 4.9 – 54.7)  Epirubicin group (N = 60): Median follow-up 35.3 months (range 7.9 – 48.1)  Doxorubicin group (N = 108): Median follow-up 23.3 months (range 4.9 – 54.7)  EPI + DOX group (N = 4): Median follow-up 40.2 months (range 21.8 – 50.0) | Type and number of participants:  N = 376 patients with soft tissue sarcoma within the high-risk group of the CWS-96 study  N = 167 excluded (unknown pre-treatment, secondary malignancy, death under therapy, progression of disease, unknown therapy, concomitant therapy with idarubicin)  N = 37 excluded (no information on late effects available)  Study group N = 172  Diagnosis:  Soft tissue sarcoma, not further specified  Age at cancer diagnosis:  Mean age total group: 8.3 years ± 5.5 SD.  Mean age epirubicin group: 7.2 years ± 5.5 SD.  Mean age doxorubicin group: 8.8 years ± 5.5 SD.  Mean age EPI + DOX group = 13.5 years ± 4.2 SD  Gender:  Total group:  Male N = 94/172 (54.7%)  Female N = 78/172 (45.3%)  Epirubicin group:  Male N = 36/60 (60.0%)  Female N = 24/60 (40.0%)  Doxorubicin group:  Male N = 56/108 (51.9%) Female N = 52/108 (48.1%)  EPI + DOX group:  Male N = 2/4 (50.0%)  Female N = 2/4 (50.0%)  Controls:  Not applicable  Cardiovascular risk factors:  Not reported  Prior cardiotoxic treatment:  Not reported  Prior cardiac dysfunction:  Not reported  Prior cardioprotective interventions:  Not reported | Anthracyclines:  Total group (N = 172): median cumulative epirubicin dose 450 mg/m^2^ (range 150-450); median doxorubicin dose 240mg/m^2^ (80-400)  N = 60/172 (34.9%) epirubicin (6h infusion, 150mg/m^2^ on 1 day per course, median cumulative dose 450mg/m^2^, range 150-450)  N = 108/172 (62.8%) doxorubicin (4h infusion, 40mg/m^2^ on 2 days per course, median cumulative dose 240mg/m^2^, range 80-400)  N = 4/172 (2.3%) with both epirubicin (median cumulative dose 225mg/m^2^, range 150-300) and doxorubicin (median cumulative dose 80mg/m^2^, range 80-160)  Mitoxantrone:  Not reported  Radiotherapy involving the heart:  Total group thoracic radiotherapy: N = 10/172 (5.8%), median 45 Gy (range 45-51)  Epirubicin group thoracic radiotherapy N = 0/60 (0%)  Doxorubicin group thoracic radiotherapy N = 10/108 (9.3%), median 45 Gy (range 45-51)  EPI + DOX group thoracic radiotherapy N = 0/4 (0%) | Diagnostic test used for cardiotoxicity assessment:  Echocardiography  Timing of the diagnostic test:  Before every anthracycline application during antineoplastic therapy and in yearly intervals after cessation of therapy  Outcome definitions:  Left ventricular function considered restricted if fractional shortening (FS) <29%.  For definition of cardiomyopathy at least 2 pathological results needed.  Subclinical cardiomyopathy defined as decreased FS and lack of clinical symptoms.  Clinical cardiomyopathy defined as additional clinical symptoms with or without cardiological therapy.  Outcome assessors blinded:  Not reported  Occurrence of acute and early-onset cardiotoxicity (not separated):  N = 3/172 (1.7%) developed cardiomyopathy during therapy.  - N = 1/3 clinical, N = 2/3 subclinical  - N = 1/3 doxorubicin group, N = 2/3 DOX+EPI group  **Fractional shortening**  *Only reported for 51 patients in which at least two examinations after therapy were available*  N = 5/51 (9.8%) had an FS <29% first six months after therapy  - N = 2/18 (11.1%) had FS <29% in epirubicin group  - N = 1/29 (3.4%) had FS <29% in doxorubicin group  *Continuous parameters*  Total group (N = 51) Mean FS 35.6% ± 4.9SD (range 26.5-47.0)  Doxorubicin group (N = 29) Mean FS 36.4 % ± 4.8SD (range 28.0 – 47.0) Epirubicin group (N = 18) Mean FS 34.9 % ± 4.5SD (range 28.0 – 44.0)  Mortality:  None (exclusion criteria mentions ‘death under therapy’)  Risk factors assessed:  Yes  Results of multivariate analyses:  Influence of type of anthracycline, thoracic irradiation, sex and age at time of diagnosis on FS assessed in multivariable model, ‘neither […] showed any influence’, but model not described | Selection bias: High risk (only 172/376 (45.7%) of original cohort included)  Attrition bias: High risk (only 125/172 (72.6%) patients had information on left ventricular function in the first year, FS only reported for 51/172 (29.7%))  Detection bias: Unclear risk (blinding of assessors is not mentioned)  Confounding: Not applicable  Reporting bias (study group): Well-defined (cumulative dose of anthracyclines and infusion duration given)  Reporting bias (follow-up): Not well defined (median of follow-up given, but no ending point defined)  Reporting bias (outcome):  Well defined (method of detection and definition of abnormal outcome provided)  Risk estimation analyses: Not applicable  Funding of the trial:  This work was  supported by the Deutsche Krebshilfe and the Madeleine  Schickedanz KinderKrebs-Stiftung  Overlap with other included studies:  Not presumed |

| **What is the frequency of occurrence of acute and early-onset cardiotoxicity, as diagnosed by clinical, echocardiographic and biochemical parameters routinely used in clinical practice, in children with cancer treated with anthracyclines, mitoxantrone and/or radiotherapy involving the heart? What are the risk factors?** | | | | |
| --- | --- | --- | --- | --- |
| *Tan et al.: Cardiotoxicity After Anthracycline Chemotherapy for Childhood Cancer in a Multiethnic Asian Population. Frontiers in Pediatrics, February 2022; volume 9.* | | | | |
| **Study design; treatment era; follow-up** | **Participants** | **Treatment** | **Diagnostic test; main outcomes** | **Risk of bias assessment** |
| Study design:  Single center retrospective cohort study.  Treatment era:  2005 – 2015  Follow-up:  Median follow-up 3.9 years (IQR 2.1 – 9.5) | Type and number of participants:  N = 458 patients with newly diagnosed cancer and receiving anthracyclines as part of their treatment protocol  Diagnosis:  N = 220/458 (48.0%) acute lymphoblastic leukemia (ALL)  N = 42/458 (9.2%) acute myeloid leukemia (AML)  N = 36/458 (7.9%) neuroblastoma  N = 44/458 (9.6%) osteosarcoma  N = 84/458 (18.3%) lymphoma  N = 17/458 (3.7%) Ewing sarcoma  N = 12/458 (2.6%) hepatoblastoma  N = 3/458 (0.7%) Others  Age at cancer diagnosis:  Median age 5.8 years (IQR 2.6 – 12.8 years)  N = 33/458 (7.2%) <1 year  N = 147/458 (32.1%) 1-4 years  N = 278/458 (60.7%) >4 years  Gender:  Male N = 282/458 (61.6%)  Female N = 176/458 (38.4%)  Controls:  Not applicable  Cardiovascular risk factors:  Not reported  Prior cardiotoxic treatment:  Not reported  Prior cardiac dysfunction:  Not reported  Prior cardioprotective interventions:  Not reported | Anthracyclines:  Median cumulative doxorubicin equivalent dose 200mg/m^2^ (IQR 120-300 mg/m2)  N = 310/458 (67.7%) cumulative doxorubicin equivalent dose <250mg/m^2^ (low dose)  N = 148/458 (32.3%) cumulative doxorubicin equivalent dose ≥250mg/m^2^ (high dose)  No infusion duration given.  ‘The cumulative dose of anthracycline was determined by conversion to doxorubicin isotoxic equivalents by multiplying the total anthracycline dose by 1 for doxorubicin and daunorubicine, 0.67 for epirubicin, 4 for mitoxantrone, 5 for idarubicin.’  Mitoxantrone:  Not reported how many children received mitoxantrone  Radiotherapy involving the heart:  N = 33/458 (7.2%) radiation involving the heart. | Diagnostic test used for cardiotoxicity assessment:  Echocardiography  Timing of the diagnostic test:  Not reported (‘patients were screened for cardiotoxicity at the discretion of the treating oncologist due to the lack of an institutional protocol during the study period’)  Outcome definitions:  Cardiotoxicity defined as left ventricular fractional shortening <28% on echocardiography.  Acute cardiotoxicity defined as onset during chemotherapy treatment.  Early cardiotoxicity defined as onset within 1 year of treatment completion.  Outcome assessors blinded:  Not reported  Occurrence of acute cardiotoxicity:  N = 3/458 (0.7%) developed acute cardiotoxicity  - N = 1/3 symptoms of heart failure  - N = 2/3 subclinical cardiotoxicity  Occurrence of early-onset cardiotoxicity:  N = 12/458 (2.6%) developed early cardiotoxicity  - N = 5/12 symptoms of heart failure  - N = 7/12 subclinical cardiotoxicity  Mortality:  N = 97/458 (21.2%) mortality during complete follow-up period  N = 15/32 (46.9%) patients in the cardiotoxicity group died during complete follow-up period.  Risk factors assessed: Univariate analysis was performed, however, early- and late-onset cardiotoxicity risk factors were not separated.  Results of multivariate analyses:  Only univariate analysis was performed. | Selection bias: Low risk (all patients in the original cohort included in the study group)  Attrition bias:  High risk (399/458 (87.1%) had echocardiograms performed during chemotherapy, of which 323/458, 70.1% had echocardiograms performed during the first year after completion of chemotherapy)  Detection bias: Unknown risk (blinding of assessors was not mentioned)  Confounding: Not applicable  Reporting bias (study group): Not well-defined (cumulative dose of anthracyclines given, but no specifics on what kind of anthracyclines and no specifics on infusion duration)  Reporting bias (follow-up): Well-defined (length of follow-up provided)  Reporting bias (outcome): Well-defined (method of detection and definition of abnormal outcome provided)  Risk estimation analyses: Not applicable  Funding of the trial: None reported  Overlap with other included studies:  Not presumed |

| **What is the frequency of occurrence of acute and early-onset cardiotoxicity, as diagnosed by clinical, echocardiographic and biochemical parameters routinely used in clinical practice, in children with cancer treated with anthracyclines, mitoxantrone and/or radiotherapy involving the heart? What are the risk factors?** | | | | |
| --- | --- | --- | --- | --- |
| *Tang et al.: The efficacy and safety of a homoharringtonine-based protocol for children with acute myeloid leukemia: A retrospective study in China. Pediatric Hematology and Oncology, 38:2, 97-107.* | | | | |
| **Study design; treatment era; follow-up** | **Participants** | **Treatment** | **Diagnostic test; main outcomes** | **Risk of bias assessment** |
| Study design:  Retrospective cohort study (single center)  Treatment era:  May 2009 to March 2015  Follow-up:  Median follow-up 62.5 months | Type and number of participants:  N = 102 newly diagnosed de novo acute myeloid leukemia (AML) patients aged 0-18 years  Original cohort unclear.  Diagnosis:  AML (acute promyelocytic leukemia excluded)  Age at cancer diagnosis:  Median age 4.75 years (range from 0.5 – 15.6 years)  N = 26/102 (25.5%) <2 years  N = 76/102 (74.5%) ≥2 years  Gender:  Male N = 57/102 (55.9%)  Female N = 45/102 (44.1%)  Controls: Not applicable  Cardiovascular risk factors: Not reported  Prior cardiotoxic treatment:  None (‘patients … who had already received treatment in other hospitals were excluded’)  Prior cardiac dysfunction: Not reported  Prior cardioprotective interventions:  Not reported | Anthracyclines:  Dose according to protocol, actual received cumulative dose not described.  Daunorubicine 40mg/m^2^ per day on days 1-3).  Cumulative dose of daunorubicine was 120mg/m^2^.  Infusion duration not reported.  Mitoxantrone:  None  Radiotherapy involving the heart:  Not reported | Diagnostic test used for cardiotoxicity assessment:  Not reported  Timing of the diagnostic test: Not reported  Outcome definitions: Not reported for cardiotoxicity  Outcome assessors blinded: Not reported for cardiotoxicity assessment  Occurrence of acute cardiotoxicity:  No separation between acute- of early-onset cardiotoxicity.  Occurrence of early-onset cardiotoxicity:  N = 0/102 (0%) reports of chemotherapy-related severe myocardial damage or impaired cardiac function  Mortality:  N = 34/102 (33.3%) died during follow-up.  Risk factors assessed: Yes, but not for cardiotoxicity (only prognostic factors for survival)  Results of multivariate analyses: Not applicable to our research question | Selection bias: Unclear risk (original cohort unclear)  Attrition bias: Unclear risk (no information provided about number of assessments for cardiotoxicity)  Detection bias: Unclear risk (no information provided about blinding of assessors for cardiotoxicity)  Confounding: Not applicable  Reporting bias (study group): Not well-defined (information on actual received cumulative anthracycline dose and infusion duration not reported)  Reporting bias (follow-up): Well-defined (length of follow-up provided)  Reporting bias (outcome): Not well-defined (no method of detection for cardiotoxicity provided, no definition of abnormal outcome provided)  Risk estimation analyses: Not applicable  Funding of the trial:  This work was partially supported by the National Natural Science Foundation of China (No. 81270623)  Overlap with other included studies:  Not presumed |

| **What is the frequency of occurrence of acute and early-onset cardiotoxicity, as diagnosed by clinical, echocardiographic and biochemical parameters routinely used in clinical practice, in children with cancer treated with anthracyclines, mitoxantrone and/or radiotherapy involving the heart? What are the risk factors?** | | | | |
| --- | --- | --- | --- | --- |
| *Tantawy et al.: Radionuclide Ventriculography Detects Early Anthracycline Cardiotoxity in Children With Hodgkin Lymphoma. J Pediatr Hematol Oncol, 2011; Volume 33 (Number 4): e132-e137* | | | | |
| **Study design; treatment era; follow-up** | **Participants** | **Treatment** | **Diagnostic test; main outcomes** | **Risk of bias assessment** |
| Study design:  Single center cohort study  Treatment era:  January 1, 2005 – December 21, 2008.  Follow-up:  Not specified (during therapy) | Type and number of participants:  N = 41 patients with Hodgkin disease (HD)  N = 2 excluded (pericardial effusion and emergency mediastinal radiotherapy)  N = 10 Group A: early during therapy, up until completion of the second ABVD cycle.  N = 29 Group B: late during therapy, after completing at least 6 ABVD cycles.  Diagnosis:  Hodgkin disease  Age at cancer diagnosis:  Group A: Mean age 8.9 years ± 3.4SD (range 4-14)  Group B: Mean age 8.4 years ± 3.3SD (range 4-14)  Gender:  Group A  Male N = 5/10 (50.0%)  Female N = 5/10 (50.0%)  Group B  Male N = 17/29 (58.6%) Female N = 12/29 (41.4%)  Controls:  Not applicable  Cardiovascular risk factors:  Not reported  Prior cardiotoxic treatment:  Inclusion criteria mentions ‘no radiotherapy before the study’  Prior cardiac dysfunction:  None (inclusion criteria mentions ‘no earlier cardiac disease and normal echocardiography before the initiation of ABVD therapy, no cardiac symptoms at the time of evaluation’)  Prior cardioprotective interventions:  Not reported | Anthracyclines:  Doxorubicin 25mg/m^2^, intravenously, day 0 and 14 of each cycle)  **Group A** = Total cumulative dose of doxorubicin mean 75mg/m^2^ ± 27.3SD (range 50-100)  **Group B** = Total cumulative dose of doxorubicin mean 328 mg/m2 ± 64SD (range 210-485)  Infusion duration not reported  Mitoxantrone:  Not reported  Radiotherapy involving the heart:  No (assessments were done before radiotherapy) | Diagnostic test used for cardiotoxicity assessment:  Echocardiography  Timing of the diagnostic test:  Group A: early during therapy, up until second ABVD cycle  Group B: late during therapy, after at least 6 ABVD cycles  Outcome definitions:  Impaired left ventricular (LV) function defined as left ventricular ejection fraction (LVEF) <50%  Outcome assessors blinded:  Not reported  Occurrence of acute and early-onset cardiotoxicity (not separated):  N = 0/39 (0%) developed clinical heart failure  N = 1/10 (10%) in group A had impaired LV function by echocardiography (LVEF <50%)  N = 11/29 (37.9%) in group B had impaired LV function by echocardiography (LVEF <50%)  *Continuous results*  **FS (%)** group A mean 41.5 ± 8.8SD, group B mean 40 ± 4SD, *P* = 0.562  **EF (%)** group A mean 58.7 ± 7.3SD, group B mean 52 ± 4.4 SD, *P* = 0.028  **E/A ratio** group A mean 1.7 ±0.4SD, group B mean 1.7 ±0.5SD, *P* = 0.907  Mortality: Not reported  Risk factors assessed: No  Results of multivariate analyses: Not reported | Selection bias:  Low risk (39/41, 95.1% of original cohort included)  Attrition bias: Low risk (all included patients were assessed for echocardiography outcome)  Detection bias: Unclear risk (blinding of assessors not mentioned)  Confounding: Not applicable  Reporting bias (study group): Well-defined (cumulative dose and of anthracyclines provided)  Reporting bias (follow-up): Not well-defined (no length of follow-up specified)  Reporting bias (outcome):  Well-defined (method of detection provided and definition of abnormal outcome (partially) provided)  Risk estimation analyses: Not applicable  Funding of the trial: Not reported  Overlap with other included studies:  Not presumed |

| **What is the frequency of occurrence of acute and early-onset cardiotoxicity, as diagnosed by clinical, echocardiographic and biochemical parameters routinely used in clinical practice, in children with cancer treated with anthracyclines, mitoxantrone and/or radiotherapy involving the heart? What are the risk factors?** | | | | |
| --- | --- | --- | --- | --- |
| *Temming et al.: Prevalence and Predictors of Anthracycline Cardiotoxicity in Children Treated for Acute Myeloid Leukaemia: Retrospective Cohort Study in a Single Centre in the United Kingdom. Pediatr Blood Cancer 2011;56:625–630* | | | | |
| **Study design; treatment era; follow-up** | **Participants** | **Treatment** | **Diagnostic test; main outcomes** | **Risk of bias assessment** |
| Study design:  Retrospective cohort study (single center)  Treatment era:  November 1987 – September 2004  Follow-up:  Median follow-up time 7.28 years (range 0.00 – 21.69), starting and end point not specified | Type and number of participants:  N = 158 patients diagnosed with acute myeloid leukemia (AML)  N = 30/158 excluded  - N = 15/30 myeloid leukemia of Down syndrome  - N = 3/30 modified treatment for secondary AML/myelodysplastic syndrome  - N = 6/30 main treatment at other centers  - N = 2/30 diagnosis other than AML  - N = 4/30 complete loss of any clinicial information  N = 128 children included for survival analysis  N = 4/128 died of leukemia before start of treatment  **N = 124 children with AML** included for retrospective analysis of cardiotoxicity  Diagnosis:  AML  Age at cancer diagnosis:  Total group: median age 2.9 years (range 0.1 – 12.9)  Gender:  Male N = 73/128 (57.0%)  Female N = 55/128 (43.0%)  Controls: Not applicable  Cardiovascular risk factors:  Not reported  Prior cardiotoxic treatment:  Not reported  Prior cardiac dysfunction:  Not reported  Prior cardioprotective interventions:  Not reported | Anthracyclines:  Dose according to protocol, actual received cumulative dose not described.  **AML 10, N = 60/128 (46.9%)**: Cumulative anthracycline dose 300mg/m^2^ (daunorubicine (DNR)) and 50mg/m^2^ mitoxantrone. Using dose equivalence ratio 1:5 for mitoxantrone/DNR total exposure was 550mg/m^2^ DNR equivalent.  **AML 12, N = 68/128 (53.1%):** Either 300mg/m^2^ DNR plus 50mg/m^2^ Mitoxantrone OR 122mg/m^2^ Mitoxantrone alone (610mg/m^2^ DNR equivalent)  Mitoxantrone and DNR were administered over 1 hour  Mitoxantrone:  See above  Radiotherapy involving the heart:  N = 8/128 (6.3%) had total body irradiation as conditioning for stem cell transplant | Diagnostic test used for cardiotoxicity assessment:  Echocardiography  Timing of the diagnostic test:  Prior to chemotherapy, at the end of treatment, 1 year after the end of treatment and 5 years after the end of treatment.  Outcome definitions:  Subclinical cardiotoxicity defined as shortening fraction (FS) <28%.  Clinical cardiomyopathy defined as clinical features of heart failure in the absence of other known causes.  Early onset = onset during treatment or within 1 year after the end of first line therapy for AML.  Outcome assessors blinded:  Not reported  Occurrence of acute and early-onset cardiotoxicity (not separated):  Data on early cardiotoxicity were available for 95/124 (76.6%); 27/124 had no documented SF during first line treatment or 1 year after and 2/124 died during the first course of therapy.  Median time of onset of early cardiotoxicity was 0.77 years (range 0.32 – 1.89) after start of treatment.  **First line treatment only** *(N = 67, 49 without and 18 with SCT)*  N = 4/67 (6.0%) children developed early subclinical cardiotoxicity with reduced SF of 23-27%.  N = 3/67 (4.5%) children developed clinical signs of cardiomyopathy within the first year after the end of treatment (N = 3/3 required ongoing therapy for cardiomyopathy and N = 2/3 died of cardiac failure)  **Second line treatment after AML relapse** *(N = 28, 10 without and 19 with SCT)*  N = 2/28 (7.1%) developed subclinical cardiotoxicity (N = 1/2 died of AML and N = 1/2 recovered SF 10 years after treatment)  N = 4/28 (14.3%) developed clinical cardiomyopathy (N = 3/4 remained on medication and N = 1/4 died of heart failure with resistant AML)  Mortality:  N = 2/67 (3.0%) died of cardiac failure in ‘first line treatment only’-group  N = 1/28 (3.6%) died of heart failure with resistant AML in ‘second line treatment after AML relapse’-group  Overall mortality  N = 49/128 (38.3%) died (N = 38/49 from AML, N = 11/49 treatment related toxicity, including 2/11 patients who died of cardiomyopathy)  Risk factors assessed: Yes, but only for late-onset cardiotoxicity  Results of multivariate analyses: Not applicable for our research question (only for late-onset cardiotoxicity) | Selection bias: High risk (only 128/158 (81.0%) of original cohort were included in survival analysis and 124/158 (78.5%) in cardiotoxicity analysis)  Attrition bias: High risk (only 66/124 (53.2%) of included patients had an outcome for early-onset cardiotoxicity)  Detection bias: Unclear risk (blinding of assessors was not mentioned)  Confounding: Not applicable  Reporting bias (study group): Not well-defined (no mean of cumulative dose of anthracyclines given, no information about dose reduction)  Reporting bias (follow-up): Well-defined (length of follow-up provided)  Reporting bias (outcome): Well-defined (method of detection and definition of abnormal outcome provided)  Risk estimation analyses: Not applicable  Funding of the trial: Not reported  Overlap with other included studies:  Not presumed |

| **What is the frequency of occurrence of acute and early-onset cardiotoxicity, as diagnosed by clinical, echocardiographic and biochemical parameters routinely used in clinical practice, in children with cancer treated with anthracyclines, mitoxantrone and/or radiotherapy involving the heart? What are the risk factors?** | | | | |
| --- | --- | --- | --- | --- |
| *Tringale et al.: Vital organ sparing with proton therapy for pediatric Hodgkin lymphoma: Toxicity and outcomes in 50 patients. Radiotherapy and Oncology, 2022; 168: 46-52.* | | | | |
| **Study design; treatment era; follow-up** | **Participants** | **Treatment** | **Diagnostic test; main outcomes** | **Risk of bias assessment** |
| Study design:  Single center retrospective cohort study  Treatment era:  2012 – 2018  Follow-up:  Median follow-up 5.3 years (range 2-8.4) | Type and number of participants:  N = 50 patients with Hodgkin lymphoma (HL) aged 11-21 years treated with proton therapy (PT)  Original cohort unclear  Diagnosis:  Hodgkin lymphoma  N = 28/50 (56%) high risk  N = 19/50 (38%) intermediate risk  N = 3/50 (6%) favorable risk  N = 2/50 (4%) relapsed/refractory.  Age at cancer diagnosis:  Age at cancer diagnosis not reported  Median age at initiation of PT was 17 years (range 11-21 years)  N = 3/50 (6.0%) <12 years  N = 34/50 (68.0%) 12-18 years  N = 13/50 (26.0%) 19-21 years  Gender:  Male N = 23/50 (46.0%)  Female N = 27/50 (54.0%)  Controls: Not applicable  Cardiovascular risk factors: Not reported  Prior cardiotoxic treatment:  N = 1/50 had received prior radiation (8.5 months prior to proton therapy, a male patient presented with a large pericardial effusion causing hemodynamic instability and ICU admission, and emergently received 9 Gy photons delivered in 3 fractions with conventional anterior/posterior radiation at an outside hospital)  Prior cardiac dysfunction: Not reported  Prior cardioprotective interventions:  Not reported | Anthracyclines:  The majority of patients received combinations of ABVE-PC (N = 34/50, 68.0%), ABVD (N = 15/50, 30.0%), or BEACOPP (N = 9/50, 18.0%).  No cumulative dosage of anthracyclines given, no information of infusion duration provided.  Mitoxantrone:  Not reported  Radiotherapy involving the heart:  N = 47/50 (94.0%) received PT to the mediastinum.  Median mean heart dose 4.3 Gy (RBE)(range 0-19.1 Gy).  Median heart V5Gy 23.8% (range 0-91.8%). | Diagnostic test used for cardiotoxicity assessment:  Echocardiography  Timing of the diagnostic test:  Follow-up at 1-2 months following completion of radiation treatment and every 6 months thereafter.  Outcome definitions:  Toxicities were graded according to the Common Terminology Criteria for Adverse Events (CTCAE) version 5.0.  Acute and subacute toxicities defined as occurring within or beyond 3 months of radiation treatment.  Late toxicities defined as occurring 1 year or more after RT.  Outcome assessors blinded: Not reported  Occurrence of acute cardiotoxicity:  N = 0/50 (0%) had acute toxicity (cardiotoxicity) correlating the CTCAE definition.  Occurrence of early-onset cardiotoxicity:  N = 1/50 (2.0%) had grade 2 toxicity left-ventricular strain (ongoing, diagnosed as anthracycline-induced cardiomyopathy)  *This patient had 4 cycles of ABVE-PC and 2 cycles of DECA (cumulatively exceeding 200mg/m^2^ doxorubicin)*  Mortality:  None died during follow-up  Risk factors assessed: No  Results of multivariate analyses: Not applicable | Selection bias: Unclear risk (original cohort unclear)  Attrition bias: Unclear risk (no information provided on how many patients were assessed for cardiotoxicity at the certain time points)  Detection bias: Unclear risk (no information provided on blinding of assessors of cardiotoxicity)  Confounding: Not applicable  Reporting bias (study group): Not well-defined (detailed information on radiotherapy dose, no information on cumulative anthracycline dosage or infusion duration)  Reporting bias (follow-up): Well-defined (length of follow-up provided)  Reporting bias (outcome): Well-defined (method of detection and definition of abnormal outcome provided)  Risk estimation analyses: Not applicable  Funding of the trial:  Grant funding from the NIH (#TL1-TR-002386).  Overlap with other included studies:  Not presumed |

| **What is the frequency of occurrence of acute and early-onset cardiotoxicity, as diagnosed by clinical, echocardiographic and biochemical parameters routinely used in clinical practice, in children with cancer treated with anthracyclines, mitoxantrone and/or radiotherapy involving the heart? What are the risk factors?** | | | | |
| --- | --- | --- | --- | --- |
| *Van Dalen et al.: Clinical heart failure in a cohort of children treated with anthracyclines: A long-term follow-up study. European Journal of Cancer, 2006: 42: 3191 - 3198* | | | | |
| **Study design; treatment era; follow-up** | **Participants** | **Treatment** | **Diagnostic test; main outcomes** | **Risk of bias assessment** |
| Study design:  Retrospective cohort study (single center)  Treatment era:  1^st^ January 1976 – 31^st^  December 2000  Follow-up:  Mean follow-up time after first dose of anthracycline 8.5 years (median 7.1 years, range 0.01-28.4) | Type and number of participants:  N = 831 patients treated with anthracyclines eligible  N = 830 patients included (N = 1 no data available)  Diagnosis:  N = 169/830 (20.4%) acute lymphoblastic leukemia (ALL)  N = 76/830 (9.2%) acute myeloid leukemia (AML)  N = 78/830 (9.4%) Hodgkin’s disease  N = 170/830 (20.5%) non-Hodgkin’s disease  N = 108/830 (13.0%) osteosarcoma  N = 73/830 (8.8%) Ewing’s sarcoma  N = 45/830 (5.4%) rhabdomyosarcoma  N = 54/830 (6.5%) Wilms’ tumor  N = 17/830 (2.0%) hepatoblastoma  N = 40/830 (4.8%) Other  Age at cancer diagnosis:  Mean age at the first dose of anthracycline therapy 8.8 years (median 8.7 years, range 0.1-18.0)  N = 76/830 (9.2%) <2 year  N = 257/830 (30.9%) 2-6 years  N = 224/830 (27.0%) 7-11 years  N = 251/830 (30.2%) 12-16 years  N = 22/830 (2.7%) >16 years  Gender:  Male N = 476/830 (57.3%)  Female N = 354/830 (42.7%)  Controls:  Not applicable  Cardiovascular risk factors: Not reported  Prior cardiotoxic treatment:  Not reported  Prior cardiac dysfunction:  Not reported  Prior cardioprotective interventions:  Not reported | Anthracyclines:  N = 830, mean cumulative dose of anthracyclines 288mg/m^2^ (median 280mg/m^2^, range 15-900)  **Cumulative dose of anthracyclines**  N = 101/830 (12.2%) <150mg/m^2^  N = 318/830 (38.3%) 150-299mg/m^2^  N = 242/830 (29.2%) 300-449mg/m^2^  N = 135/830 (16.3%) 450-600mg/m^2^  N = 15/830 (1.8%) >600mg/m^2^  N = 19/830 (2.3%) unknown  **Anthracycline specifics**  N = 435/830 (52.4%) only doxorubicin  N = 66/830 (8.0%) only daunorubicin  N = 152/830 (18.3%) only epirubicin  N = 1/830 (0.1%) only idarubicin  N = 176/830 (21.2%) combination of doxorubicin, daunorubicine, epirubicin and/or idarubicin.  ‘Different durations of anthracycline infusion were used, both bolus and continuous (uptil 48h). The daily anthracycline dose varied between 13 and 150mg/m^2^ and the maximal peak dose varied between 15 and 180mg/m^2^.’  Mitoxantrone:  N = 34/830 (4.1%) received mitoxantrone, mean cumulative dose 21.8mg/m^2^ (median 12, range 12-108)  N = 29/34 (85.3%) received <40mg/m^2^  N = 5/34 (14.7%) received ≥40mg/m^2^  N = 3/830 (0.4%) had unknown status for mitoxantrone  Radiotherapy involving the heart:  N = 176/830 (21.2%) received radiotherapy involving the heart  N = 1/830 (0.1%) had unknown status about radiotherapy of the heart  N = 653/830 (78.7%) had not received radiotherapy of the heart  N = 47/830 (5.7%) received dexrazoxane  N = 1/830 (0.1%) had unknown status for dexrazoxane  N = 782/830 (94.2%) had not received dexrazoxane | Diagnostic test used for cardiotoxicity assessment:  Clinical assessment  Timing of the diagnostic test:  Not specified  Outcome definitions:  A-CHF (anthracycline induced clinical heart failure) defined as congestive heart failure, not attributable to other known causes.  Congestive heart failure defined as the presence of following clinical signs: dyspnea, pulmonary oedema, peripheral oedema and/or exercise intolerance, which were treated with anticongestive therapy.  Early A-CHF defined as during anthracycline therapy or within the first year after the end of treatment.  Outcome assessors blinded: For diagnosis A-CHF: yes (cardiologist was unaware of cumulative anthracycline dose)  Occurrence of acute and early-onset cardiotoxicity (not separated):  N = 16/830 (1.9%) cases of early A-CHF  *N = 15/830 using our definition of early-onset cardiotoxicity, i.e. within one year after start of therapy.*  N = 3/15 (20.0%) died  N = 1/15 (6.7%) received dexrazoxane  N = 2/15 (13.3%) had radiotherapy of the heart  Mortality:  Early- and late onset mortality: N = 297/830 (35.8%) died (N = 287 due to tumour-related causes, N = 4 other causes, N = 6 cardiac deaths)  Risk factors assessed: Yes, but only for early- and late onset cardiotoxicity combined  Results of multivariate analyses: Not applicable for our research question | Selection bias: Low risk (830/831 (99.9%) of original cohort included)  Attrition bias: Low risk (clinical status obtained up to 2002 for 795/830 (95.8%) of cohort, for the other 35 patients data of last known follow-up date used)  Detection bias: Low risk (assessor (cardiologist) for A-CHF was blinded for anthracycline status)  Confounding: Not applicable  Reporting bias (study group): Well-defined (detailed information about cumulative doses and infusion duration for anthracyclines and mitoxantrone)  Reporting bias (follow-up): Well-defined (length of follow-up provided as well as starting point of follow-up)  Reporting bias (outcome): Well defined (method of detection and definition of abnormal outcome provided)  Risk estimation analyses: Not applicable  Funding of the trial:  This study was supported by the Foundation of Paediatric Cancer Research (SKK), Amsterdam, the Netherlands, and the  Jacques H de Jong Foundation, Nieuwegein, the Netherlands.  Overlap with other included studies:  Possible overlap with Kremer 2002, but different outcomes reported |

| **What is the frequency of occurrence of acute and early-onset cardiotoxicity, as diagnosed by clinical, echocardiographic and biochemical parameters routinely used in clinical practice, in children with cancer treated with anthracyclines, mitoxantrone and/or radiotherapy involving the heart? What are the risk factors?** | | | | |
| --- | --- | --- | --- | --- |
| *Yu et al.: Anthracycline Induced Cardiac Disorders in Childhood Acute Lymphoblastic Leukemia: A Single-Centre, Retrospective, Observational Study. Frontiers in Pharmacology, 2021; volume 12.* | | | | |
| **Study design; treatment era; follow-up** | **Participants** | **Treatment** | **Diagnostic test; main outcomes** | **Risk of bias assessment** |
| Study design:  Single center retrospective observational study  Treatment era:  January 1, 2015 to December 31, 2018  Follow-up:  Not reported | Type and number of participants:  N = 204 patients with newly diagnosed acute lymphoblastic leukemia (ALL).  N = 33/204 (16.2%) were excluded for lack of information about cardiac parameters, congenital heart disease or abnormal baseline ECG/echocardiogram.  **N = 171 eligible patients**  Diagnosis:  ALL  N = 91/171 (53.2%) low risk  N = 68/171 (39.8%) intermediate risk  N = 12/171 (7.0%) high risk  Age at cancer diagnosis:  Median age 5.07 ± 3.19 years (IQR 3-7, range 7 months – 14 years)  N = 16/171 (9.4%) 0-1 year  N = 134/171 (78.4%) 2-10 years  N = 21/171 (12.3%) ≥10 years  Gender:  Male N = 108/171 (63.2%)  Female N = 63/171 (36.8%)  Controls:  Not applicable  Cardiovascular risk factors:  Not reported  Prior cardiotoxic treatment: Not reported  Prior cardiac dysfunction:  None (exclusion criteria mentions ‘pre-existing cardiovascular diseases or abnormal baseline electrocardiogram (ECG) or echocardiography’)  Prior cardioprotective interventions:  Not reported | Anthracyclines:  N = 84/171 (49.1%) cumulative daunorubicin dosage ≤75mg/m^2^  N = 87/171 (50.9%) cumulative daunorubicin dosage >75mg/m^2^  N = 91/171 (53.2%) low risk patients:  daunorubicin 25mg/m^2^ on days 5 and 12 in induction and daunorubicin 25mg/m^2^ in reinduction treatment.  N = 80/171 (46.8%) intermediate/high risk patients: daunorubicin 25mg/m^2^ on days 5 and 12 in induction and  daunorubicin 25mg/m^2^ in 5 cycles at continuation treatment.  Infusion duration not reported.  Mitoxantrone:  Not reported  Radiotherapy involving the heart:  Not reported | Diagnostic test used for cardiotoxicity assessment:  Echocardiogram  Timing of the diagnostic test:  Before start of induction, consolidation, continuation, reinduction, and maintenance courses.  Outcome definitions:  Cardiac disorders were defined according to the Common Terminology Criteria for Adverse Events (CTCAE) Version 5.0.  Early cardiotoxicity is defined as onset within one year of chemotherapy.  Sub-acute and acute cardiotoxicity occur between the time of chemotherapy initiation to up to two weeks after completion of therapy.  Outcome assessors blinded: Not reported  Occurrence of acute cardiotoxicity:  Not separated from early-onset cardiotoxicity  Occurrence of early-onset cardiotoxicity:  Total of N = 78/171 (45.6%) developed cardiac disorders  N = 4/171 (2.3%) suffered from clinical cardiotoxicity after administration of daunorubicin.  N = 74/171 (43.3%) developed subclinical cardiac disorders by regular cardiac examinations (echo/ECG)  N = 31/171 (18.1%) showed echo abnormalities.  N = 14/31 (45.2%) small amount of pericardial effusion.  N = 11/31 (35.5%) LV hypertrophy  N = 5/31 (16.1%) widened pulmonary artery  N = 5/31 (16.1%) valve disease  Mortality:  Not reported  Risk factors assessed: Yes, but only in binary logistic regression  Results of multivariate analyses:  Not applicable | Selection bias: High risk (171/204 (83.8%) of original cohort included)  Attrition bias:  Unclear risk (no information provided on how many eligible patients were assessed for the assessments such as echocardiography)  Detection bias: Unclear risk (no information provided on blinding of assessors)  Confounding: Not applicable  Reporting bias (study group): Not well-defined (cumulative dosage of anthracycline provided in dichotomous matter, no information about infusion duration)  Reporting bias (follow-up): Not well-defined (no information about length of follow-up provided)  Reporting bias (outcome):  Well-defined (method of detection and definition of abnormal outcome provided)  Risk estimation analyses: Not applicable  Funding of the trial:  Funded by Natural Science Foundation of Hubei Province (No. WJ2019Q032 and No. 2019CFB495) and National Key R&D Program of China (No. 2019FC1316203)  Overlap with other included studies:  Not presumed |
