# Supplementary material for: Acute and early-onset cardiotoxicity in children and adolescents with cancer: a systematic review
Source: BMC Cancer. 2023 Sep 14;23:866. doi: 10.1186/s12885-023-11353-9 (PMC10500898; doi:10.1186/s12885-023-11353-9)
Supplement: Supplementary file 4 — Additional file 4. Overview of oncologic diagnoses. [file 12885_2023_11353_MOESM4_ESM.docx]

**Additional file 4: Overview of oncologic diagnoses**

| Diagnosis | Number of subjects | Percentage of all subjects |
| --- | --- | --- |
| Hematologic malignancies | 5992 | 76.9 |
| Acute lymphoblastic leukemia | 2736 | 35.1 |
| Acute myeloid leukemia | 2348 | 30.1 |
| Non-Hodgkin lymphoma | 436 | 5.6 |
| Hodgkin lymphoma | 234 | 3.0 |
| Hematologic malignancies (other/not specified) | 238 | 3.1 |
| Solid malignancies | 1646 | 21.1 |
| Osteosarcoma | 447 | 5.7 |
| Ewing sarcoma | 370 | 4.7 |
| Soft tissue sarcoma | 291 | 3.7 |
| Hepatoblastoma | 260 | 3.3 |
| Neuroblastoma | 141 | 1.8 |
| Wilms tumor | 107 | 1.4 |
| Solid malignancies (other/not specified) | 30 | 0.4 |
| Oncologic diagnosis not specified | 159 | 2.0 |
| All subjects | 7797 | 100 |
