# Supplementary material for: Acute and early-onset cardiotoxicity in children and adolescents with cancer: a systematic review
Source: BMC Cancer. 2023 Sep 14;23:866. doi: 10.1186/s12885-023-11353-9 (PMC10500898; doi:10.1186/s12885-023-11353-9)
Supplement: Supplementary file 5 — Additional file 5. Risk of bias in included studies. [file 12885_2023_11353_MOESM5_ESM.docx]

**Additional file 5: Risk of bias in included studies**

|  | Internal validity | | | | | | External validity | | | |
| --- | --- | --- | --- | --- | --- | --- | --- | --- | --- | --- |
|  | Study group: selection bias | Follow-up: attrition bias | Outcome: detection bias | | | Risk estimation: confounding | Study group: reporting bias | Follow-up: reporting bias | Outcome: reporting bias | Risk estimation: analysis |
|  |  |  | Echocardiography | Biomarkers | Clinical assessment |  |  |  |  |  |
| Agha 2016 | ● | ● | ● | ● | ● | ● | ● | ● | ● | ● |
| Al-Biltagi 2012 | ● | ● | ● | ● | ● | ● | ● | ● | ● | ● |
| Asselin 2016 | ● | ● | ● | ● | ● | ● | ● | ● | ● | ● |
| Berrak 2001 | ● | ● | ● | ● | ● | ● | ● | ● | ● | ● |
| Brown 2013 | ● | ● | ● | ● | ● | ● | ● | ● | ● | ● |
| Burke 2021 | ● | ● | ● | ● | ● | ● | ● | ● | ● | ● |
| Chen 2009 | ● | ● | ● | ● | ● | ● | ● | ● | ● | ● |
| Cheung 2020 | ● | ● | ● | ● | ● | ● | ● | ● | ● | ● |
| Choi 2010 | ● | ● | ● | ● | ● | ● | ● | ● | ● | ● |
| Creutzig 2007 | ● | ● | ● | ● | ● | ● | ● | ● | ● | ● |
| De Matos Neto 2006 | ● | ● | ● | ● | ● | ● | ● | ● | ● | ● |
| El Amrousy 2022 | ● | ● | ● | ● | ● | ● | ● | ● | ● | ● |
| El-Shitany 2012 | ● | ● | ● | ● | ● | ● | ● | ● | ● | ● |
| Erkus 2007 | ● | ● | ● | ● | ● | ● | ● | ● | ● | ● |
| Fukumi 2002 | ● | ● | ● | ● | ● | ● | ● | ● | ● | ● |
| Getz 2019 | ● | ● | ● | ● | ● | ● | ● | ● | ● | ● |
| Gupta 2018 | ● | ● | ● | ● | ● | ● | ● | ● | ● | ● |
| Hagag 2019 | ● | ● | ● | ● | ● | ● | ● | ● | ● | ● |
| Hu 2018 (1) | ● | ● | ● | ● | ● | ● | ● | ● | ● | ● |
| Hu 2018 (2) | ● | ● | ● | ● | ● | ● | ● | ● | ● | ● |
| Ishii 2000 | ● | ● | ● | ● | ● | ● | ● | ● | ● | ● |
| Kang 2012 | ● | ● | ● | ● | ● | ● | ● | ● | ● | ● |
| Katzenstein 2022 | ● | ● | ● | ● | ● | ● | ● | ● | ● | ● |
| Khairat 2019 | ● | ● | ● | ● | ● | ● | ● | ● | ● | ● |
| Kremer 2002 | ● | ● | ● | ● | ● | ● | ● | ● | ● | ● |
| Krischke 2016 | ● | ● | ● | ● | ● | ● | ● | ● | ● | ● |
| Linares Ballesteros 2021 | ● | ● | ● | ● | ● | ● | ● | ● | ● | ● |
| Mavinkurve-Groothuis 2013 | ● | ● | ● | ● | ● | ● | ● | ● | ● | ● |
| Moke 2018 | ● | ● | ● | ● | ● | ● | ● | ● | ● | ● |
| Moussa 2017 | ● | ● | ● | ● | ● | ● | ● | ● | ● | ● |
| Moyo 2021 | ● | ● | ● | ● | ● | ● | ● | ● | ● | ● |
| Oztarhan 2011 | ● | ● | ● | ● | ● | ● | ● | ● | ● | ● |
| Radu 2019 | ● | ● | ● | ● | ● | ● | ● | ● | ● | ● |
| Sági 2018 | ● | ● | ● | ● | ● | ● | ● | ● | ● | ● |
| Samosir 2021 | ● | ● | ● | ● | ● | ● | ● | ● | ● | ● |
| Schramm 2019 | ● | ● | ● | ● | ● | ● | ● | ● | ● | ● |
| Shaikh 2013 | ● | ● | ● | ● | ● | ● | ● | ● | ● | ● |
| Stöhr 2006 | ● | ● | ● | ● | ● | ● | ● | ● | ● | ● |
| Tan 2021 | ● | ● | ● | ● | ● | ● | ● | ● | ● | ● |
| Tang 2020 | ● | ● | ● | ● | ● | ● | ● | ● | ● | ● |
| Tantawy 2011 | ● | ● | ● | ● | ● | ● | ● | ● | ● | ● |
| Temming 2011 | ● | ● | ● | ● | ● | ● | ● | ● | ● | ● |
| Tringale 2022 | ● | ● | ● | ● | ● | ● | ● | ● | ● | ● |
| Van Dalen 2006 | ● | ● | ● | ● | ● | ● | ● | ● | ● | ● |
| Yu 2021 | ● | ● | ● | ● | ● | ● | ● | ● | ● | ● |

Legend: ●, low risk (internal validity items)/well-defined (external validity items); ●, high risk (internal validity items)/not well-defined (external validity items); ●, unclear risk; ●, risk differed for different outcomes and/or different time points (see text for explanation); ●, not applicable
